# Coordinated Optimization of Visual Cortical Maps (I) Symmetry-based Analysis

**DOI:** 10.1371/journal.pcbi.1002466

**Published:** 2012-11-08

**Authors:** Lars Reichl, Dominik Heide, Siegrid Löwel, Justin C. Crowley, Matthias Kaschube, Fred Wolf

**Affiliations:** 1 Max-Planck-Institute for Dynamics and Self-Organization, Göttingen, Germany; 2 Bernstein Center for Computational Neuroscience, Göttingen, Germany; 3 Bernstein Focus Neurotechnology, Göttingen, Germany; 4 Faculty of Physics, Georg-August University, Göttingen, Germany; 5 Frankfurt Institute of Advanced Studies, Frankfurt, Germany; 6 School of Biology, Georg-August University, Göttingen, Germany; 7 Carnegie Mellon University, Department of Biological Sciences, Pittsburgh, Pennsylvania, United States of America; 8 Physics Department and Lewis-Sigler Institute, Princeton University, Princeton, New Jersey, United States of America; 9 Kavli Institute for Theoretical Physics, University of California, Santa Barbara, California, United States of America; Indiana University, United States of America

## Abstract

In the primary visual cortex of primates and carnivores, functional architecture can be characterized by maps of various stimulus features such as orientation preference (OP), ocular dominance (OD), and spatial frequency. It is a long-standing question in theoretical neuroscience whether the observed maps should be interpreted as optima of a specific energy functional that summarizes the design principles of cortical functional architecture. A rigorous evaluation of this optimization hypothesis is particularly demanded by recent evidence that the functional architecture of orientation columns precisely follows species invariant quantitative laws. Because it would be desirable to infer the form of such an optimization principle from the biological data, the optimization approach to explain cortical functional architecture raises the following questions: i) What are the genuine ground states of candidate energy functionals and how can they be calculated with precision and rigor? ii) How do differences in candidate optimization principles impact on the predicted map structure and conversely what can be learned about a hypothetical underlying optimization principle from observations on map structure? iii) Is there a way to analyze the coordinated organization of cortical maps predicted by optimization principles in general? To answer these questions we developed a general dynamical systems approach to the combined optimization of visual cortical maps of OP and another scalar feature such as OD or spatial frequency preference. From basic symmetry assumptions we obtain a comprehensive phenomenological classification of possible inter-map coupling energies and examine representative examples. We show that each individual coupling energy leads to a different class of OP solutions with different correlations among the maps such that inferences about the optimization principle from map layout appear viable. We systematically assess whether quantitative laws resembling experimental observations can result from the coordinated optimization of orientation columns with other feature maps.

## Author Summary

Neurons in the visual cortex form spatial representations or maps of several stimulus features. How are different spatial representations of visual information coordinated in the brain? In this paper, we study the hypothesis that the coordinated organization of several visual cortical maps can be explained by joint optimization. Previous attempts to explain the spatial layout of functional maps in the visual cortex proposed specific optimization principles ad hoc. Here, we systematically analyze how optimization principles in a general class of models impact on the spatial layout of visual cortical maps. For each considered optimization principle we identify the corresponding optima and analyze their spatial layout. This directly demonstrates that by studying map layout and geometric inter-map correlations one can substantially constrain the underlying optimization principle. In particular, we study whether such optimization principles can lead to spatially complex patterns and to geometric correlations among cortical maps as observed in imaging experiments.

## Introduction

Neurons in the primary visual cortex are selective to a multidimensional set of visual stimulus features, including visual field position, contour orientation, ocular dominance, direction of motion, and spatial frequency [1], [2]. In many mammals, these response properties form spatially complex, two-dimensional patterns called visual cortical maps [3]–[25]. The functional advantage of a two dimensional mapping of stimulus selectivities is currently unknown [26]–[28]. What determines the precise spatial organization of these maps? It is a plausible hypothesis that natural selection should shape visual cortical maps to build efficient representations of visual information improving the ‘fitness’ of the organism. Cortical maps are therefore often viewed as optima of some cost function. For instance, it has been proposed that cortical maps optimize the cortical wiring length [29], [30] or represent an optimal compromise between stimulus coverage and map continuity [31]–[44]. If map structure was largely genetically determined, map structure might be optimized through genetic variation and Darwinian selection on an evolutionary timescale. Optimization may, however, also occur during the ontogenetic maturation of the individual organism for instance by the activity-dependent refinement of neuronal circuits. If such an activity-dependent refinement of cortical architecture realizes an optimization strategy its outcome should be interpreted as the convergence towards a ground state of a specific energy functional. This hypothesized optimized functional, however, remains currently unknown. As several different functional maps coexist in the visual cortex candidate energy functionals are expected to reflect the multiple response properties of neurons in the visual cortex. In fact, consistent with the idea of joint optimization of different feature maps cortical maps are not independent of each other [8], [10], [19], [23], [42], [45]–[48]. Various studies proposed a coordinated optimization of different feature maps [31], [33], [34], [37], [38], [40]–[42], [44], [49]–[51]. Coordinated optimization appears consistent with the observed distinct spatial relationships between different maps such as the tendency of iso-orientation lines to intersect OD borders perpendicularly or the preferential positioning of orientation pinwheels at locations of maximal eye dominance [8], [10], [19], [23], [42], [45], [47]. Specifically these geometric correlations have thus been proposed to indicate the optimization of a cost function given by a compromise between stimulus coverage and continuity [33], [35], [38], [40], [42], [44], a conclusion that was questioned by Carreira-Perpinan and Goodhill [52].

Visual cortical maps are often spatially complex patterns that contain defect structures such as point singularities (pinwheels) [6], [12], [53], [54], [55] or line discontinuities (fractures) [13], [56] and that never exactly repeat [3]–[10], [12]–[25], [57]. It is conceivable that this spatial complexity arises from geometric frustration due to a coordinated optimization of multiple feature maps in which not all inter-map interactions can be simultaneously satisfied [51], [58]–[61]. In many optimization models, however, the resulting map layout is spatially not complex or lacks some of the basic features such as topological defects [29], [51], [58], [62], [63]. In other studies coordinated optimization was reported to preserve defects that would otherwise decay [51], [58]. An attempt to rigorously study the hypothesis that the structure of cortical maps is explained by an optimization process thus raises a number of questions: i) What are the genuine ground states of candidate energy functionals and how can they be calculated with precision and rigor? ii) How do differences in candidate optimization principles impact on the predicted map structure and conversely what can be learned about an hypothetical underlying optimization principle from observations on map structure? iii) Is there a way to analyze the coordinated organization of cortical maps predicted by optimization principles in general? If theoretical neuroscience was able to answer these questions with greater confidence, the interpretation and explanation of visual cortical architecture could build on a more solid foundation than currently available. To start laying such a foundation, we examined how symmetry principles in general constrain the form of optimization models and developed a formalism for analyzing map optimization independent of the specific energy functional assumed.

Minima of a given energy functional can be found by gradient descent which is naturally represented by a dynamical system describing a formal time evolution of the maps. Response properties in visual cortical maps are arranged in repetitive modules of a typical spatial length called hypercolumn. Optimization models that reproduce this typical length scale are therefore effectively pattern forming systems with a so-called ‘cellular’ or finite wavelength instability, see [64]–[66]. In the theory of pattern formation, it is well understood that symmetries play a crucial role [64]–[66]. Some symmetries are widely considered biologically plausible for cortical maps, for instance the invariance under spatial translations and rotations or a global shift of orientation preference [51], [63], [67]–[71]. In this paper we argue that such symmetries and an approach that utilizes the analogy between map optimization and pattern forming systems can open up a novel and systematic approach to the coordinated optimization of visual cortical representations.

A recent study found strong evidence for a common design in the functional architecture of orientation columns [3]. Three species, galagos, ferrets, and tree shrews, widely separated in evolution of modern mammals, share an apparently universal set of quantitative properties. The average pinwheel density as well as the spatial organization of pinwheels within orientation hypercolumns, expressed in the statistics of nearest neighbors as well as the local variability of the pinwheel densities in cortical subregions ranging from 1 to 30 hypercolumns, are found to be virtually identical in the analyzed species. However, these quantities are different from random maps. Intriguingly, the average pinwheel density was found to be statistical indistinguishable from the mathematical constant 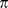 up to a precision of 2%. Such apparently universal laws can be reproduced in relatively simple self-organization models if long-range neuronal interactions are dominant 3,70–72. As pointed out by Kaschube and coworkers, these findings pose strong constraints on models of cortical functional architecture [3]. Many models exhibiting pinwheel annihilation [51], [58] or pinwheel crystallization [62], [63], [73] were found to violate the experimentally observed layout rules. In [3] it was shown that the common design is correctly predicted in models that describe long-range interactions within the OP map but no coupling to other maps. Alternatively, however, it is conceivable that they result from geometric frustration due to inter-map interactions and joint optimization. In the current study we therefore in particular examined whether the coordinated optimization of the OP map and another feature map can reproduce the quantitative laws defining the common design.

The presentation of our results is organized as follows. First we introduce a formalism to model the coordinated optimization of complex and real valued scalar fields. Complex valued fields can represent for instance orientation preference (OP) or direction preference maps [14], [24]. Real valued fields may represent for instance ocular dominance (OD) [1], spatial frequency maps [20], [45] or ON-OFF segregation [74]. We construct several optimization models such that an independent optimization of each map in isolation results in a regular OP stripe pattern and, depending on the relative representations of the two eyes, OD patterns with a regular hexagonal or stripe layout. A model-free, symmetry-based analysis of potential optimization principles that couple the real and complex valued fields provides a comprehensive classification and parametrization of conceivable coordinated optimization models and identifies representative forms of coupling energies. For analytical treatment of the optimization problem we adapt a perturbation method from pattern formation theory called weakly nonlinear analysis [64]–[66], [75]–[78]. This method is applicable to models in which the spatial pattern of columns branches off continuously from an unselective homogeneous state. It reduces the dimensionality of the system and leads to amplitude equations as an approximate description of the system near the symmetry breaking transition at which the homogeneous state becomes unstable. We identify a limit in which inter-map interactions that are formally always bidirectional become effectively unidirectional. In this limit, one can neglect the backreaction of the complex map on the layout of the co-evolving scalar feature map. We show how to treat low and higher order versions of inter-map coupling energies which enter at different order in the perturbative expansion.

Second we apply the derived formalism by calculating optima of two representative low order examples of coordinated optimization models and examine how they impact on the resulting map layout. Two higher order optimization models are analyzed in Text S1. For concreteness and motivated by recent topical interest [3], [79], [80], we illustrate the coordinated optimization of visual cortical maps for the widely studied example of a complex OP map and a real feature map such as the OD map. OP maps are characterized by pinwheels, regions in which columns preferring all possible orientations are organized around a common center in a radial fashion [53], [55], [81], [82]. In particular, we address the problem of pinwheel stability in OP maps [51], [71] and calculate the pinwheel densities predicted by different models. As shown previously, many theoretical models of visual cortical development and optimization fail to predict OP maps possessing stable pinwheels [29], [51], [58], [62]. We show that in case of the low order energies, a strong inter-map coupling will typically lead to OP map suppression, causing the orientation selectivity of all neurons to vanish. For all considered optimization models, we identify stationary solutions of the resulting dynamics and mathematically demonstrate their stability. We further calculate phase diagrams as a function of the inter-map coupling strength and the amount of overrepresentation of certain stimuli of the co-evolving scalar feature map. We show that the optimization of any of the analyzed coupling energies can lead to spatially relatively complex patterns. Moreover, in case of OP maps, these patterns are typically pinwheel-rich. The phase diagrams, however, differ for each considered coupling energy, in particular leading to coupling energy specific ground states. We therefore thoroughly analyze the spatial layout of energetic ground states and in particular their geometric inter-map relationships. We find that none of the examined models reproduces the experimentally observed pinwheel density and spatially aperiodic arrangements. Our analysis identifies a seemingly general condition for interaction induced pinwheel-rich OP optima namely a substantial bias in the response properties of the co-evolving scalar feature map.

## Results

### Modeling the coordinated optimization of multiple maps

We model the response properties of neuronal populations in the visual cortex by two-dimensional scalar order parameter fields which are either complex valued or real valued [53], [83]. A complex valued field 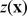 can for instance describe OP or direction preference of a neuron located at position 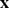. A real valued field 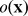 can describe for instance OD or the spatial frequency preference. Although we introduce a model for the coordinated optimization of general real and complex valued order parameter fields we consider 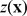 as the field of OP and 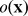 as the field of OD throughout this article. In this case, the pattern of preferred stimulus orientation 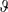 is obtained by
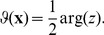
(1)The modulus 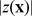 is a measure of the selectivity at cortical location 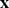.

OP maps are characterized by so-called *pinwheels*, regions in which columns preferring all possible orientations are organized around a common center in a radial fashion. The centers of pinwheels are point discontinuities of the field 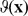 where the mean orientation preference of nearby columns changes by 90 degrees. Pinwheels can be characterized by a topological charge 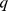 which indicates in particular whether the orientation preference increases clockwise or counterclockwise around the pinwheel center,
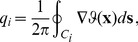
(2)where 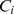 is a closed curve around a single pinwheel center at 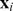. Since 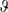 is a cyclic variable in the interval 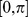 and up to isolated points is a continuous function of 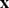, 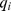 can only have values
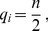
(3)where 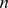 is an integer number [84]. If its absolute value 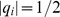, each orientation is represented only once in the vicinity of a pinwheel center. In experiments, only pinwheels with a topological charge of 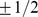 are observed, which are simple zeros of the field 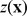.

OD maps can be described by a real valued two-dimensional field 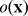, where 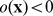 indicates ipsilateral eye dominance and 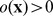 contralateral eye dominance of the neuron located at position 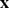. The magnitude indicates the strength of the eye dominance and thus the zeros of the field correspond to the borders of OD.

In this article, we view visual cortical maps as optima of some energy functional 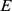. The time evolution of these maps can be described by the gradient descent of this energy functional. The field dynamics thus takes the form
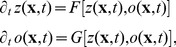
(4)where 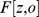 and 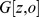 are nonlinear operators given by 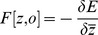, 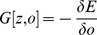. The system then relaxes towards the minima of the energy 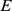. The convergence of this dynamics towards an attractor is assumed to represent the process of maturation and optimization of the cortical circuitry. Various biologically detailed models have been cast to this form [35], [51], [85].

All visual cortical maps are arranged in repetitive patterns of a typical wavelength 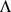. We splitted the energy functional 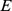 into a part that ensures the emergence of such a typical wavelength for each map and into a part which describes the coupling among different maps. A well studied model reproducing the emergence of a typical wavelength by a pattern forming instability is of the Swift-Hohenberg type [65], [86]. Many other pattern forming systems occurring in different physical, chemical, and biological contexts (see for instance [75]–[78]) have been cast into a dynamics of this type. Its dynamics in case of the OP map is of the form
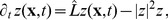
(5)with the linear Swift-Hohenberg operator
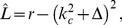
(6)

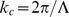
, and 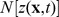 a nonlinear operator. The energy functional of this dynamics is given by
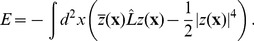
(7)In Fourier representation, 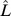 is diagonal with the spectrum
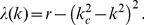
(8)The spectrum exhibits a maximum at 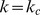. For 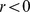, all modes are damped since 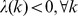 and only the homogeneous state 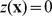 is stable. This is no longer the case for 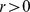 when modes on the *critical circle* 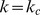 acquire a positive growth rate and now start to grow, resulting in patterns with a typical wavelength 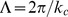. Thus, this model exhibits a supercritical bifurcation where the homogeneous state looses its stability and spatial modulations start to grow.

The coupled dynamics we considered is of the form
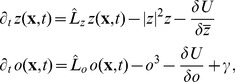
(9)where 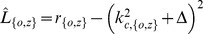, and 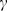 is a constant. To account for the species differences in the wavelengths of the pattern we chose two typical wavelengths 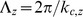 and 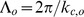. The dynamics of 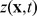 and 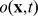 is coupled by interaction terms which can be derived from a coupling energy 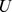. In the uncoupled case this dynamics leads to pinwheel free OP stripe patterns.

### Symmetries constrain inter-map coupling energies

How many inter-map coupling energies 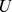 exist? Using a phenomenological approach the inclusion and exclusion of various terms has to be strictly justified. We did this by symmetry considerations. The constant 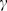 breaks the inversion symmetry 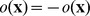 of inputs from the ipsilateral (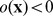) or contralateral (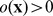) eye. Such an inversion symmetry breaking could also arise from quadratic terms such as 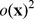. In the methods section we detail how a constant shift in the field 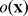 can eliminate the constant term and generate such a quadratic term. Including either a shift or a quadratic term thus already represents the most general case. The inter-map coupling energy 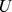 was assumed to be invariant under this inversion. Otherwise orientation selective neurons would, for an equal representation of the two eyes, develop different layouts to inputs from the left or the right eye. The primary visual cortex shows no anatomical indication that there are any prominent regions or directions parallel to the cortical layers [67]. Besides invariance under translations 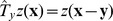 and rotations 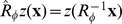 of both maps we required that the dynamics should be invariant under orientation shifts 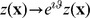. Note, that the assumption of shift symmetry is an idealization that uncouples the OP map from the map of visual space. Bressloff and coworkers have presented arguments that Euclidean symmetry that couples spatial locations to orientation shift represents a more plausible symmetry for visual cortical dynamics [68], [87], see also [88]. The existence of orientation shift symmetry, however, is not an all or none question. Recent evidence in fact indicates that shift symmetry is only weakly broken in the spatial organization of orientation maps [89], [90]. A general coupling energy term can be expressed by integral operators which can be written as a Volterra series
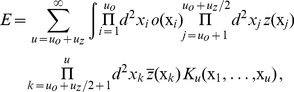
(10)with an 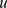-th. order integral kernel 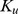. Inversion symmetry and orientation shift symmetry require 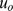 to be even and that the number of fields 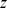 equals the number of fields 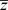. The lowest order term, mediating an interaction between the fields 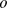 and 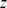 is given by 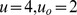 i.e.
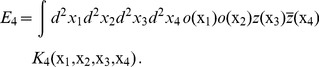
(11)Next, we rewrite Eq. (11) as an integral over an energy density 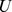. We use the invariance under translations to introduce new coordinates
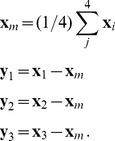
(12)This leads to
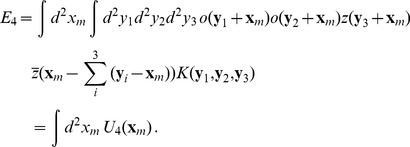
(13)The kernel 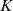 may contain local and non-local contributions. Map interactions were assumed to be local. For local interactions the integral kernel is independent of the locations 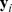. We expanded both fields in a Taylor series around 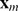


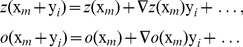
(14)For a local energy density we could truncate this expansion at the first order in the derivatives. The energy density can thus be written
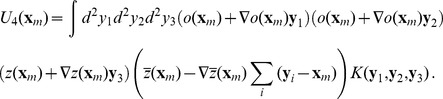
(15)Due to rotation symmetry this energy density should be invariant under a simultaneous rotation of both fields. From all possible combinations of Eq. (15) only those are invariant in which the gradients of the fields appear as scalar products. The energy density can thus be written as
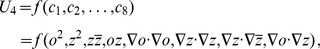
(16)where we suppress the argument 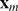. All combinations 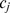 can also enter via their complex conjugate. The general expression for 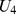 is therefore
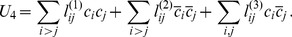
(17)From all possible combinations we selected those which are invariant under orientation shifts and eye inversions. This leads to
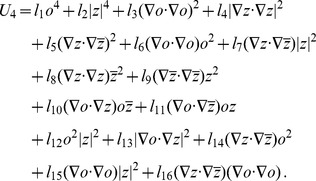
(18)The energy densities with prefactor 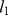 to 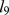 do not mediate a coupling between OD and OP fields and can be absorbed into the single field energy functionals. The densities with prefactors 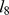 and 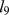 (also with 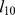 and 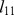) are complex and can occur only together with 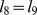 (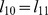) to be real. These energy densities, however, are not bounded from below as their real and imaginary parts can have arbitrary positive and negative values. The lowest order terms which are real and positive definite are thus given by
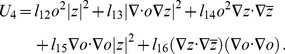
(19)The next higher order energy terms are given by
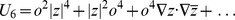
(20)Here the fields 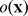 and 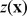 enter with an unequal power. In the corresponding field equations these interaction terms enter either in the linear part or in the cubic nonlinearity. We will show in this article that interaction terms that enter in the linear part of the dynamics can lead to a suppression of the pattern and possibly to an instability of the pattern solution. Therefore we considered also higher order interaction terms.

These higher order terms contain combinations of terms in Eq. (19) and are given by
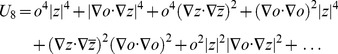
(21)As we will show below examples of coupling energies
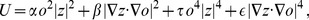
(22)form a representative set that can be expected to reproduce experimentally observed map relationships. For this choice of energy the corresponding interaction terms in the dynamics Eq. (9) are given by
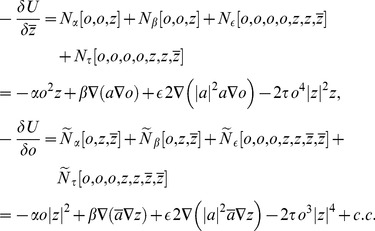
(23)with 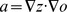 and 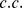 denoting the complex conjugate. In general, all coupling energies in 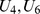, and 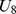 can occur in the dynamics and we restrict to those energies which are expected to reproduce the observed geometric relationships between OP and OD maps. It is important to note that with this restriction we did not miss any essential parts of the model. When using weakly nonlinear analysis the general form of the near threshold dynamics is insensitive to the used type of coupling energy and we therefore expect similar results also for the remaining coupling energies.

**Figure 1:**
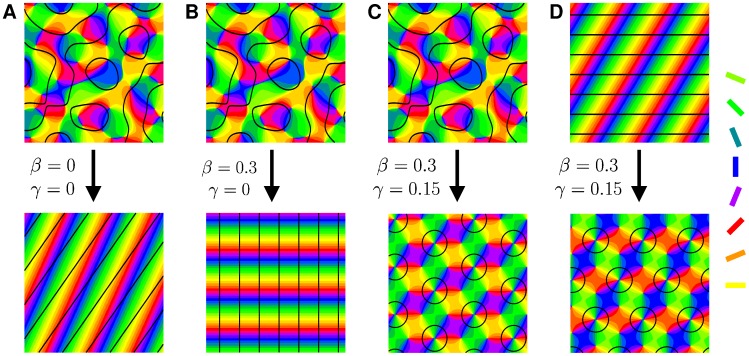
Pinwheel annihilation, preservation, and generation in numerical simulations for different strengths of inter-map coupling and OD bias 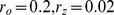 **.** Color code of OP map with zero contours of OD map superimposed. **A** 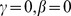 **B** 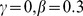 **C** and **D** 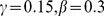. Initial conditions identical in **A**–**C**, 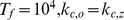.

Numerical simulations of the dynamics Eq. (9), see [63], [91], with the coupling energy Eq. (22) and 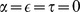 are shown in Fig. 1. The initial conditions and final states are shown for different bias terms 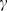 and inter-map coupling strengths 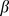. We observed that for a substantial contralateral bias and above a critical inter-map coupling pinwheels are preserved from random initial conditions or are generated if the initial condition is pinwheel free. Without a contralateral bias the final states were pinwheel free stripe solutions irrespective of the strength of the inter-map coupling.

### Calculating ground states by coupled amplitude equations

We studied Eq. (9) with the low order inter-map coupling energies in Eq. (22) using weakly nonlinear analysis. We therefore rewrite Eq. (9) as
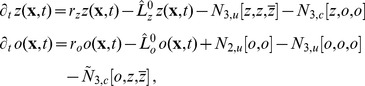
(24)where we shifted both linear operators as 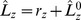, 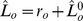. The constant term 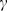 in Eq. (9) is replaced by a quadratic interaction term 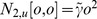 with 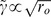, see Methods. The uncoupled nonlinearities are given by 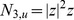, 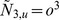 while 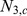 and 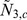 are the nonlinearities of the low order inter-map coupling energy Eq. (23). We study Eq. (24) close to the pattern forming bifurcation where 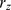 and 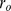 are small. We therefore expand both control parameters in powers of the small expansion parameter 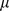


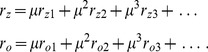
(25)Close to the bifurcation the fields are small and thus nonlinearities are weak. We therefore expand both fields as
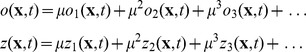
(26)We further introduced a common slow timescale 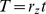 and insert the expansions in Eq. (24) and get
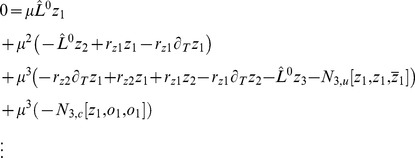
(27)and
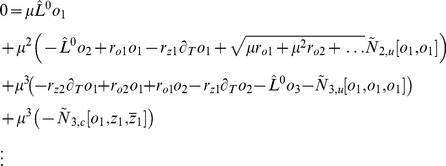
(28)We consider amplitude equations up to third order as this is the order where the nonlinearity of the low order inter-map coupling energy enters first. For Eq. (27) and Eq. (28) to be fulfilled each individual order in 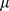 has to be zero. At linear order in  we get the two homogeneous equations
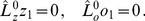
(29)Thus 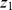 and 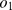 are elements of the kernel of 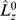 and 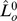. Both kernels contain linear combinations of modes with a wavevector on the corresponding critical circle i.e.
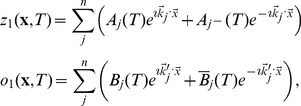
(30)with the complex amplitudes 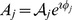, 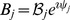 and 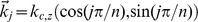, 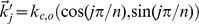. In view of the hexagonal or stripe layout of the OD pattern shown in Fig. 1, 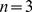 is an appropriate choice. In the following sections we assume 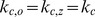 i.e. the Fourier components of the emerging pattern are located on a common circle. To account for species differences we also analyzed models with detuned OP and OD wavelengths in part (II) of this study.

At second order in 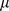 we get
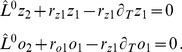
(31)As 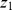 and 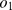 are elements of the kernel 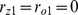. At third order, when applying the solvability condition (see Methods), we get
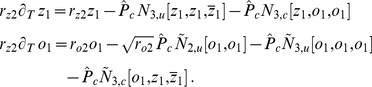
(32)We insert the leading order fields Eq. (30) and obtain the amplitude equations
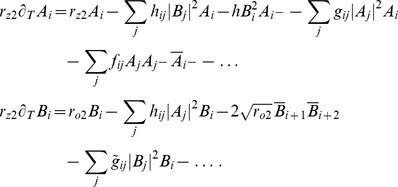
(33)For simplicity we have written only the simplest inter-map coupling terms. Depending on the configuration of active modes additional contributions may enter the amplitude equations. In addition, for the product-type coupling energy, there are coupling terms which contain the constant 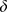, see Methods and Eq. (40). The coupling coefficients are given by
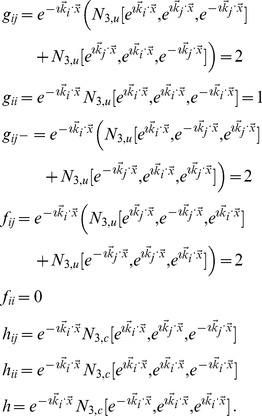
(34)From Eq. (33) we see that inter-map coupling has two effects on the modes of the OP pattern. First, inter-map coupling shifts the bifurcation point from 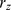 to 
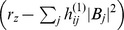
. This can cause a potential destabilization of pattern solutions for large inter-map coupling strength. Second, inter-map coupling introduces additional resonant interactions that for instance couple the modes 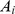 and their opposite modes 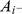. In case of 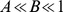 the inter-map coupling terms in dynamics of the modes 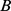 are small. In this limit the dynamics of the modes 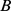 decouples from the modes 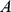 and we can use the uncoupled OD dynamics, see Methods. When we scale back to the fast time variable and set 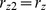, 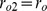 we obtain
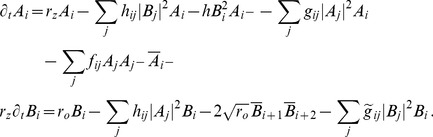
(35)The amplitude equations are truncated at third order. If pattern formation takes place somewhat further above threshold fifth order, seventh order, or even higher order corrections are expected to become significant and can induce quantitative modifications of the low order solutions. If third order approximate solutions exhibit degeneracies or marginal stability, higher orders of perturbation theory will qualitatively change the solutions. However, none of the solutions found in the studied models was only marginally stable. This suggests that the obtained solutions are in general structurally stable. A derivation of amplitude equation with higher order inter-map coupling energies is presented in Text S1.

### Interpretation of coupling energies

Using symmetry considerations we derived inter-map coupling energies up to eighth order in the fields, see Eq. (19), Eq.(20), and Eq.(21). Which of these various optimization principles could reproduce realistic inter-map relationships such as a uniform coverage of all stimulus features? We identified two types of optimization principles that can be expected to reproduce realistic inter-map relationships and good stimulus coverage. First, product-type coupling energies of the form 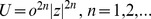. These energies favor configurations in which regions of high gradients avoid each other and thus leading to high coverage. Second, gradient-type coupling energies of the form 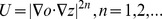. In experimentally obtained maps, iso-orientation lines show the tendency to intersect the OD borders perpendicularly. Perpendicular intersection angles lead to high coverage as large changes of the field 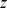 in one direction lead to small changes of the field 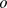 in that direction. To see that the gradient-type coupling energy favors perpendicular intersection angles we decompose the complex field 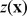 into the selectivity 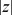 and the preferred orientation 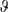. We obtain
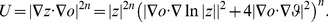
(36)If the orientation selectivity is locally homogeneous, i.e. 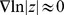, then the energy is minimized if the direction of the iso-orientation lines (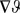) is perpendicular to the OD borders. In our symmetry-based analysis we further identified terms that are expected to lead to the opposite behavior for instance mixture terms such as 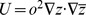.

Pinwheels are prominent features in OP maps. We therefore also analyze how different optimization principles impact on the pinwheel positions with respect to the co-evolving feature maps. The product-type coupling energies are expected to favor pinwheels at OD extrema. Pinwheels are zeros of 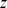 and are thus expected to reduce this energy term. They will reduce energy the most when 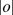 is maximal which should repel pinwheels from OD borders, where 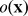 is zero. Also the gradient-type coupling energy is expected to couple the OD pattern with the position of pinwheels. To see this we decompose the field 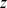 into its real and imaginary part
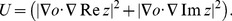
(37)At pinwheel centers the zero contours of 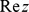 and 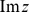 cross. Since there 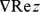 and 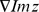 are almost constant and not parallel the energy can be minimized only if 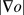 is small at the pinwheel centers, i.e. the extrema or saddle-points of 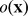.

From the previous considerations we assume all coupling coefficients of the energies to be positive. A negative coupling coefficient can be saturated by higher order inter-map coupling terms. In the following, we discuss the impact of the low order inter-map coupling energies on the resulting optima of the system using the derived amplitude equations. The corresponding analysis for higher order inter-map coupling energies is provided in Text S1.

### Optima of particular optimization principles: Low order coupling terms

As indicated by numerical simulations and weakly nonlinear analysis of the uncoupled OD dynamics, see Methods, we discussed the influence of the OD stripe, hexagon, and constant solutions on the OP map using the coupled amplitude equations derived in the previous section. A potential backreaction onto the dynamics of the OD map can be neglected if the modes 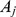 of the OP map are much smaller than the modes 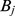 of the OD map. This can be achieved if 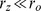. We first give a brief description of the uncoupled OP solutions. Next, we study the impact of the low order coupling energies in Eq. (22) on these solutions. We demonstrate that these energies can lead to a complete suppression of orientation selectivity. In the uncoupled case there are for 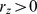 two stable stationary solutions to the amplitude equations Eq. (35), namely OP stripes
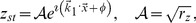
(38)and OP rhombic solutions
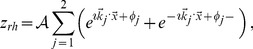
(39)with 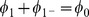, 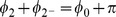, 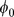 an arbitrary phase, and 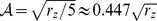. In the uncoupled case the angle 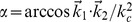 between the Fourier modes is arbitrary. The stripe solutions are pinwheel free while the pinwheel density for the rhombic solutions varies as 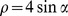 and thus 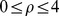. For the rhombic solutions pinwheels are located on a regular lattice. We therefore refer to these and other pinwheel rich solutions which are spatially periodic as pinwheel crystals (PWC). In particular, we refer to pinwheel crystals with as rhombic spatial layout as rPWC solutions and pinwheel crystals with a hexagonal layout as hPWC solutions. Without inter-map coupling, the potential of the two solutions reads 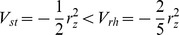, thus the stripe solutions are always energetically preferred compared to rhombic solutions.

In the following we study three scenarios in which inter-map coupling can lead to pinwheel stabilization. First, a deformation of the OP stripe solution can lead to the creation of pinwheels in this solution. Second, inter-map coupling can energetically prefer the (deformed) OP rhombic solutions compared to the stripe solutions. Finally, inter-map coupling can lead to the stabilization of new PWC solutions.

For the low order interaction terms the amplitude equations are given by 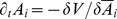, 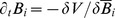 with the potential
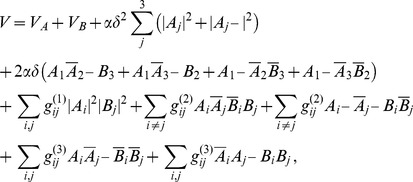
(40)with the uncoupled contributions
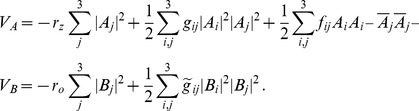
(41)The coupling coefficients read 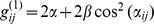, 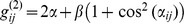, 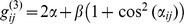, 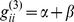, where 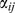 is the angle between the wavevector 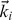 and 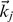.

### Product-type energy 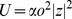


We first studied the impact of the low order product-type coupling energy. Here, the constant 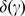 enters explicitly in the amplitude equations, see Eq. (40) and Eq. (68).

#### Stationary solutions and their stability

**Figure 2:**
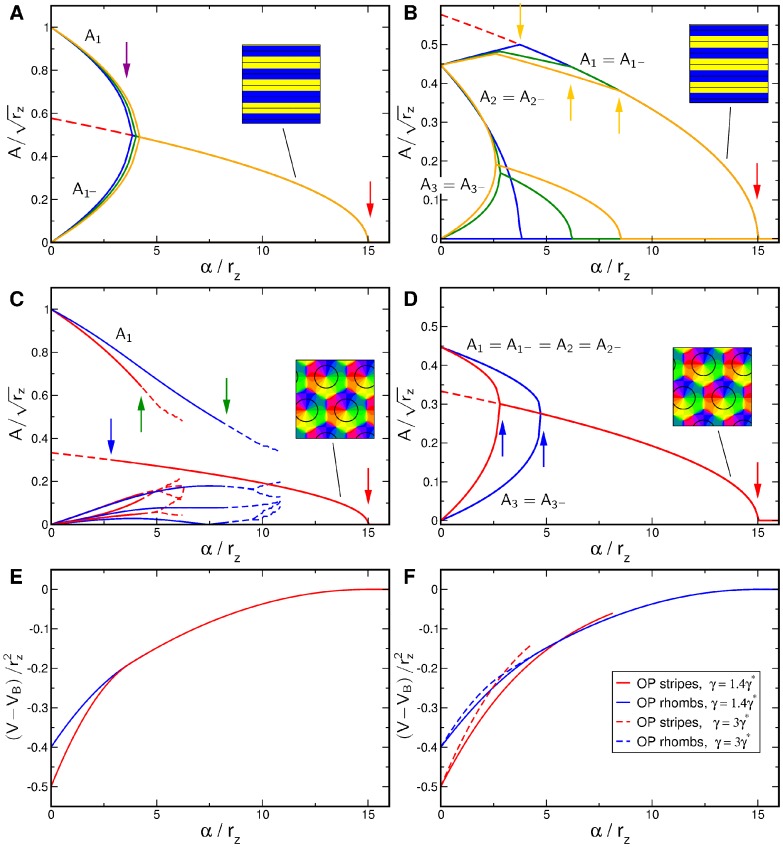
Stationary amplitudes with coupling energy 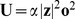 **,** 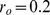 **.** Solid (dashed) lines: stable (unstable) solutions to Eq. (42) (OD stripes) and Eq. (51) (OD hexagons). **A,B** OD stripes, 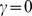 (blue), 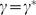 (green), 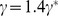 (orange). **C,D** OD hexagons, 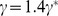 (blue), 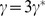 (red). **A,C** Transition from OP stripe solutions, **B,D** Transition from OP rhombic solutions. **E** Potential for OP stripes (red) and OP rhombs (blue) interacting with OD stripes, 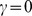. **F** Potential for OP stripes and OP rhombs interacting with OD hexagons. Arrows indicate corresponding lines in the phase diagram, Fig. (3).

In the case of OD stripes, see Methods, with 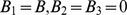 we get the following amplitude equations
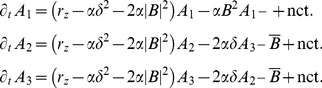
(42)where nct. refers to non inter-map coupling terms 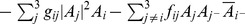, resulting from the potential 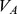, see Eq. (41). The equations for the modes 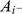 are given by interchanging the modes 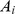 and 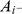 as well as interchanging the modes 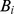 and 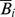. The OP stripe solution in case of inter-map coupling is given by
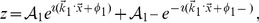
(43)with 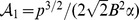, 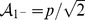, and 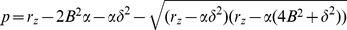 and the phase relation 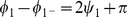. In the uncoupled case (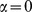) they reduce to 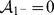 and 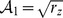. With increasing inter-map coupling the amplitude 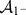 grows and the solutions are transformed, reducing the representation of all but two preferred orientations. The parameter dependence of this solution is shown in Fig. 2**A** for different values of the bias 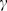. Both amplitudes become identical at 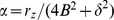 with
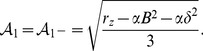
(44)This pattern solution finally vanishes at
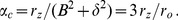
(45)This existence border is thus independent of the OD bias 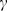. Above this coupling strength only the trivial solution 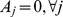 is stable.

In addition to the OP stripe patterns there exist rhombic OP solutions, see Fig. 2**B**. These rhombic solutions are pinwheel rich with a pinwheel density of 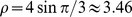 but are energetically not preferred compared to the stripe solutions, see Fig. 2**E**. The rhombic solutions in the uncoupled case 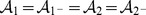, 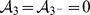 are transformed by inter-map coupling. The phase relations are given by
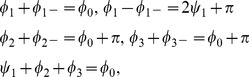
(46)where 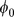 is an arbitrary phase. Stationary amplitudes are given by
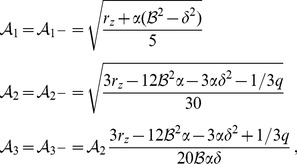
(47)with 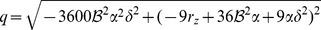. With increasing inter-map coupling strength 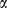 the amplitudes 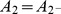 are suppressed, see Fig. (2)**B**. In addition, for nonzero bias 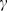, there is an increase of the amplitudes 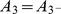. The amplitudes 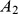 and 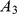 collapse at 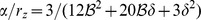. A further increase of the inter-map coupling strength leads to a suppression of these amplitudes and finally to the OP stripe pattern where 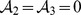.

In the case the OD map is a constant, Eq. (91), the amplitude equations simplify to
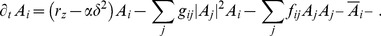
(48)Thus inter-map coupling in this case only renormalizes the bifurcation parameter and the energetic ground state is thus a stripe pattern with an inter-map coupling dependent reduction of the amplitudes
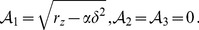
(49)Therefore at 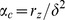 the stripe pattern ceases to exist and the only stable solution is the trivial one i.e. 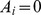. In addition, there is the rhombic solution with the stationary amplitudes
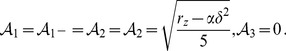
(50)In the case of OD hexagons 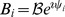, Eq. (90), the amplitude equations read
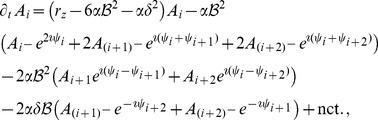
(51)where the indices are cyclic i.e. 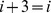. These amplitude equations have stripe-like solutions as well as solutions with a rhombic layout of the form 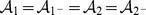, 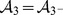. For both solutions the stationary phases depend on the inter-map coupling strength 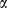. In contrast to the case of OD stripes and OD constant solutions the amplitude equations (51) have an additional type of PWC solution which have uniform amplitudes, 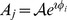. The dynamics of their phases is given by
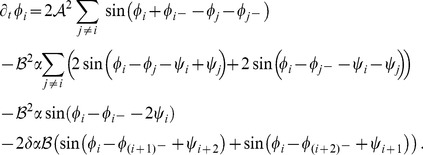
(52)When solving the amplitude equations numerically we observe that the phase relations vary with the inter-map coupling strength for non-uniform solutions. But for the uniform solution the phase relations are independent of the inter-map coupling strength. The phases of the uniform solution are determined up to a free phase 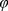 which results from the orientation shift symmetry 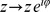 of Eq. (9). We therefore choose 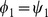. As an ansatz for the uniform solutions we use
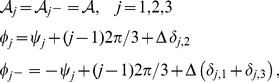
(53)where 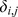 is the Kronecker delta and 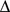 a constant parameter. Note, that 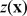 cannot become real since 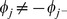. The equation for the uniform amplitudes is then given by
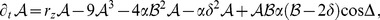
(54)while the phase dynamics reads
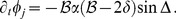
(55)The stationarity condition is fulfilled for an arbitrary 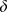 only if 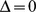 or 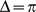. The corresponding amplitudes are given by solving the stationarity condition for the real part and read
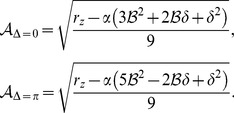
(56)We calculate the stability properties of all solutions by linear stability analysis considering perturbations of the amplitudes 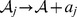, 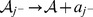 and of the phases 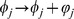, 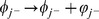. This leads to a perturbation matrix 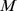. Amplitude and phase perturbations in general do not decouple. We calculated the eigenvalues of the perturbation 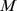 matrix numerically and checked the results by direct numerical simulation of the amplitude equations. In case of the uniform solutions Eq. (53) the perturbation matrix 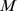 is explicitly stated in Text S3.

The stability of the 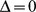 and 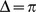 uniform solutions depends on the coupling strength 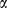 and on the sign of 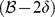. As the solution of 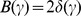 is given by 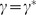 in the stability range of OD hexagons there is only one possible stable uniform solution, the 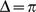 uniform solution. This solution ceases to exist at 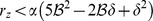. This existence border is in fact independent of the bias 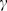 and given by
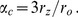
(57)Thus the limit 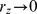 makes the uniform solution unstable for smaller and smaller coupling strengths.

#### Bifurcation diagram

For increasing inter-map coupling strength the amplitudes of the OP solutions are shown in Fig. 2. In case of inter-map coupling strength dependent stationary phases, stationary solutions are calculated numerically using a Newton method and initial conditions close to these solutions. We followed the unstable solutions (dashed lines in Fig. 2) until this method did not converge anymore. Not shown are solutions which are unstable in general. The case of OP solutions when interacting with OD stripes is shown in Fig. 2**A,B**. In case of OP stripes inter-map coupling suppresses the amplitude 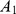 of the stripe pattern while increasing the amplitude of the opposite mode 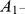. This transformation reduces the representation of all but two preferred orientations. When both amplitudes collapse the resulting OP map is selective only to two orthogonal orientations namely 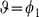 and 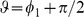. We refer to these unrealistic solutions as *orientation scotoma solutions*. The phase relations ensure that OD borders that run parallel to the OP stripes are located at the OP maxima and minima i.e. in the center of the orientation scotoma stripes. With increasing inter-map coupling, this orientation scotoma pattern is suppressed until finally all amplitudes are zero and only the homogeneous solution is stable. In case of OP rhombs inter-map coupling makes the rhombic pattern more stripe-like by reducing the amplitude 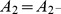. The mode 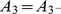 which is zero in the uncoupled case increases and finally collapses with the mode 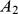. Increasing inter-map coupling more suppresses all but the two modes 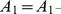, leading again to the orientation scotoma stripe pattern.

The parameter dependence of OP solutions when interacting with OD hexagons is shown in Fig. 2**C,D**. OP stripe solutions became above a critical inter-map coupling strength unstable against PWC solutions. This critical coupling strength strongly depended on the OD bias. OP rhombic solutions also became unstable against PWC but for a lower coupling strength than the OP stripes. Thus there is at intermediate coupling strength a bistability between stripe-like solutions and PWC solutions. The potential of the OP stripe and OP rhombic solutions is shown in Fig. 2**E,F**. Stripes are energetically preferred in the uncoupled case as well as for small inter-map coupling strength for which they are stable.

To summarize, stripe solutions were deformed but no pinwheels were created for this solution. The rhombic solutions were energetically not preferred for low inter-map coupling whereas for intermediate inter-map coupling these solutions lose pinwheels and became stripe solutions. Instead, additional pinwheel rich solutions with a crystal layout became stable for intermediate inter-map coupling. For large inter-map coupling orientation selectivity was completely suppressed.

#### Phase diagram

**Figure 3:**
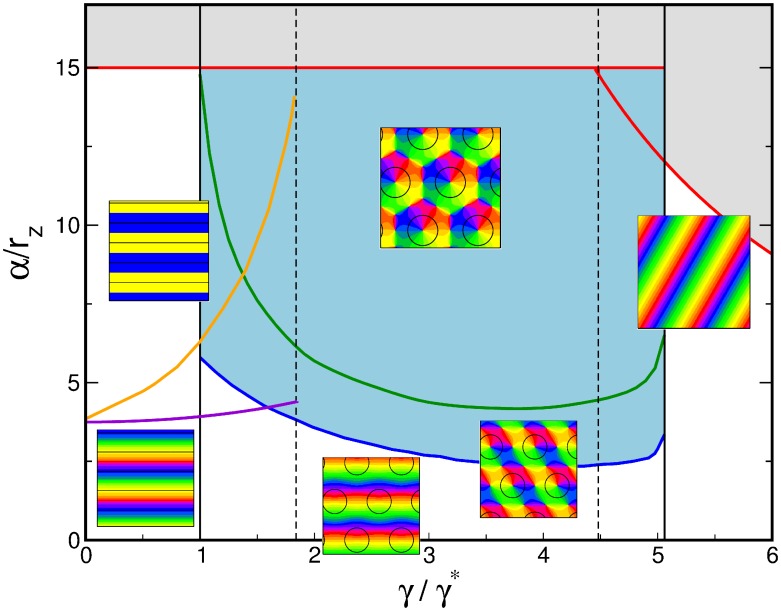
Phase diagram with the coupling energy 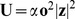, 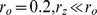. Vertical lines: stability range of OD stripes, hexagons, and constant solution. Magenta (orange) line: transition of stripes (rhombs) to the orientation scotoma solution. Blue line: stability border for the 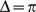 uniform solution (hPWC). Green line: stability line of stripe-like solutions. Red line: pattern solutions ceases to exist, see Eq. (45) and Eq. (57). Blue region: stability region of hPWC, gray region: No pattern solution exists.

The phase diagram as a function of the OD bias 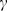 and the inter-map coupling strength 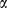 for this coupling energy is shown in Fig. 3. When rescaling the inter-map coupling strength as 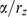 the phase diagram is independent of the bifurcation parameter of the OP map 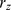. Thus for fixed 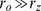 the phase diagram depends only on two control parameters 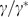 and 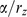. The phase diagram contains the stability borders of the uncoupled OD solutions 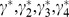. They correspond to vertical lines, as they are independent of the inter-map coupling in the limit 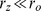. At 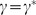 hexagons become stable. Stripe solutions become unstable at 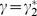. At 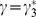 the homogeneous solution becomes stable while at 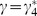 hexagons lose their stability. In the units 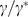 the borders 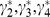 vary slightly with 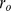 , see Methods, and are drawn here for 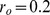. Colored lines correspond to the stability and existence borders of OP solutions. In the region of stable OD stripes the OP stripes run parallel to the OD stripes. With increasing inter-map coupling strength the orientation preference of all but two orthogonal orientations is suppressed. In the region of stable OD hexagons stripe-like OP solutions dominate for low inter-map coupling strength. Above a critical bias dependent coupling strength the 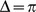 uniform solution becomes stable (blue line). There is a region of bistability between stripe-like and uniform solutions until the stripe-like solutions lose their stability (orange line). OP rhombic solutions lose their stability when the uniform solution becomes stable. Thus there is no bistability between OP rhombs and OP uniform solutions. As in the case of OD stripes the uniform solution becomes unstable at 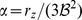. Also in the case of OD hexagons the inter-map coupling leads to a transition towards the trivial solution where there is no OP pattern at all. In case of the OD constant solution the OP map is a stripe solution. Pinwheel rich solutions thus occur only in the region of stable OD hexagons. In the following we discuss the properties of these solutions.

#### Interaction induced pinwheel crystals

**Figure 4:**
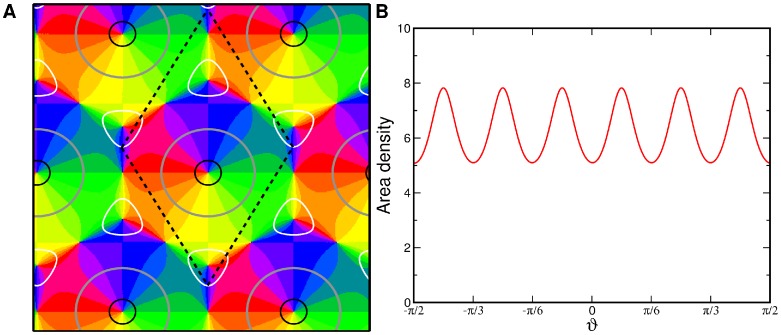
Ipsi-center pinwheel crystal. **A** OP map, superimposed are the OD borders (gray), 90% ipsilateral eye dominance (black), and 90% contralateral eye dominance (white), 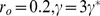. Dashed lines mark the unit cell of the regular pattern. **B** Distribution of preferred orientations.

The uniform solution Eq. (53) with 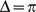 is illustrated in Fig. 4. For all stationary solutions the positions of the pinwheels are fixed by the OD map and there are no translational degrees of freedom. The unit cell (dashed line) contains 6 pinwheels which leads to a pinwheel density of 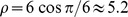. Two of them are located at OD maxima (contra center) while one is located at an OD minimum (ipsi center). The remaining three pinwheels are located at OD saddle-points. Therefore, all pinwheels are located where the gradient of the OD map is zero. The pinwheel in the center of the OP hexagon is at the ipsilateral OD peak. Because these pinwheels organize most of the map while the others essentially only match one OP hexagon to its neighbors we refer to this pinwheel crystal as the *Ipsi-center pinwheel crystal*. The iso-orientation lines intersect the OD borders (gray) exactly with a right angle. The intersection angles are, within the stability range of OD hexagons, independent of the bias 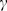. The remarkable property of perfect intersection angles cannot be deduced directly from the coupling energy term. The solution is symmetric under a combined rotation by 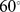 and an orientation shift by 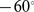. The symmetry of the pattern is reflected by the distribution of preferred orientations, see Fig. 4**B**. Although the pattern is selective to all orientations the six orientations 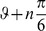, 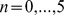 are slightly overrepresented.

To summarize, the low order product-type inter-map coupling leads in case of OD hexagons to a transition from pinwheel free stripe solutions towards pinwheel crystals. The design of the PWC is an example of an orientation hypercolumn dominated by one pinwheel. With increasing inter-map coupling the PWC solution is suppressed until only the homogeneous solution is stable. In case of OD stripes or the constant solution the OP solutions are pinwheel free stripe pattern.

### Gradient-type energy 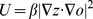


When using a gradient-type inter-map coupling energy the interaction terms are independent of the OD shift 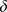. In this case, the coupling strength can be rescaled as 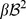 and is therefore independent of the bias 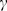. The bias in this case only determines the stability of OD stripes, hexagons or the constant solution.

#### Stationary solutions and their stability

A coupling to OD stripes is easy to analyze in the case of a gradient-type inter-map coupling. The energetically preferred solutions are OP stripes with the direction perpendicular to the OD stripes for which 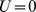. This configuration corresponds to the Hubel and Wiesel Ice-cube model [1]. In addition there are rPWC solutions with the stationary amplitudes 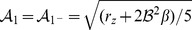, 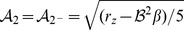, 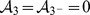, and the stationary phases as in Eq. (46). Increasing inter-map coupling strength 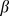 leads to an increase of the amplitudes 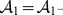 while decreasing the amplitudes 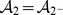 thus making the rhombic solution more stripe-like.

In the case the OD map is a constant, Eq. (91), the gradient-type inter-map coupling leaves the OP dynamics unaffected. The stationary states are therefore OP stripes with an arbitrary direction and rPWC solutions as in the uncoupled case.

In the case of OD hexagons the amplitude equations read
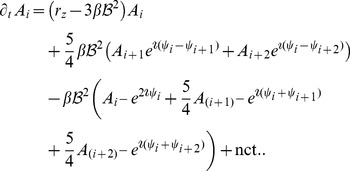
(58)Using 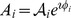 we obtain the phase equations
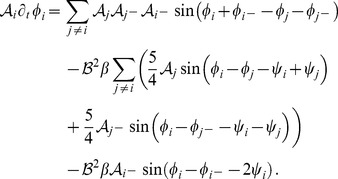
(59)These amplitude equations have stripe-like and rhombic solutions with inter-map coupling dependent phase relations. Besides stripe-like and rhombic solutions these amplitude equations also have uniform solutions. Again we find that the ansatz Eq. (53) can satisfy the stationarity condition. The phase dynamics in this case reads
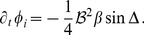
(60)As in the case of the product-type inter-map coupling energy stationary solutions are 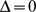 and 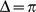 with the stationary amplitudes
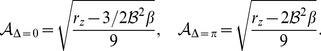
(61)We studied the stability properties of both stationary solutions by linear stability analysis where amplitude and phase perturbations in general do not decouple. The stability matrix of the uniform solutions is given in Text S3. The eigenvalues are calculated numerically. It turned out that the 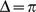 solution is unstable for 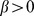 while the 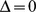 solution becomes stable for 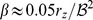. The 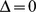 solution loses its stability above
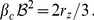
(62)From thereon only the homogeneous solution 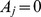 is stable.

#### Bifurcation diagram

**Figure 5:**
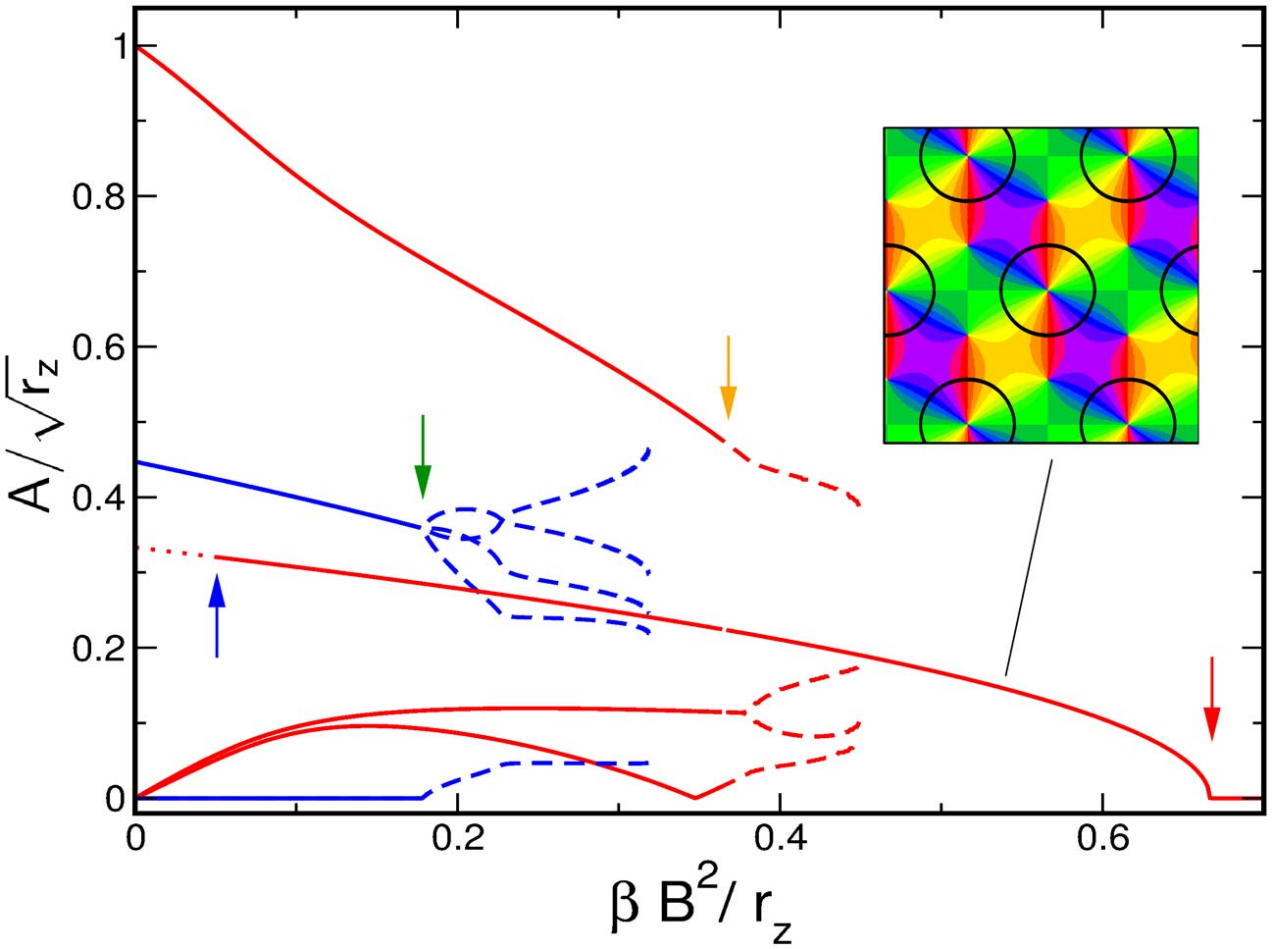
Stationary amplitudes with 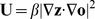 and OD hexagons. Solid (dashed) lines: Stable (unstable) solutions to Eq. (58). Transition from OP stripes towards the uniform solution (red), transition from OP rhombs towards the uniform solution (blue). Arrows indicate corresponding lines in the phase diagram, Fig. (6).

The course of the stationary amplitudes when interacting with OD hexagons is shown in Fig. 5. In case of inter-map coupling strength dependent stationary phases, stationary solutions are calculated numerically using a Newton method and initial conditions close to these solutions. We followed the unstable solutions (dashed lines in Fig. 5) until this method did not converge anymore. Not shown are solutions which are unstable in general. The OP rhombic solution is almost unchanged by inter-map coupling but above a critical coupling strength the rhombs decay into a stripe-like solution. The amplitude of the OP stripe solution is suppressed by inter-map coupling and finally becomes unstable against the 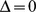 uniform solution. Thus for large inter-map coupling only the uniform solution is stable. A further increase in the inter-map coupling suppresses the amplitude of this uniform solution until finally only the homogeneous solution is stable.

#### Phase diagram

**Figure 6:**
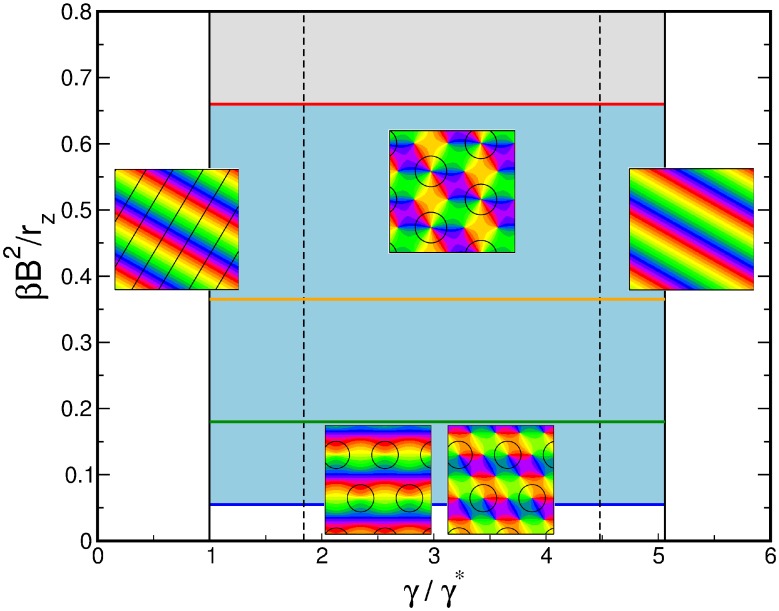
Phase diagram with the coupling energy 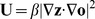, 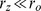. Vertical black lines: stability range of OD stripes, hexagons, and constant solution. Blue line: stability border for the 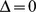 uniform solution. Green line: rhombic solutions become unstable. Orange line: stripe-like solutions become unstable. Red line: pattern solutions cease to exist, see Eq. (61). Gray region: No pattern solution exists.

The phase diagram of this coupling energy is shown in Fig. 6. We rescaled the inter-map coupling strength as 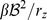, where 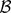 is the stationary amplitude of the OD hexagons. The stability borders are then independent of the OD bias in the OD solutions and further independent of the bifurcation parameter 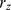. This simplifies the analysis since the OP solutions and their stability depend on 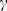 only indirect via the amplitudes 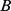. In case of OD stripes or OD constant solution there is no pinwheel crystallization. Instead the OP solutions are pinwheel free stripes. In case of OD hexagons hPWCs become stable above 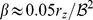 (blue line). Rhombic OP patterns become unstable at 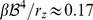 and decay into a stripe-like solution (green line). At 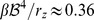 these stripe-like solutions become unstable (orange line). Thus above 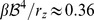 the hPWC is the only stable solution. At 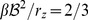 the pattern solution ceases to exist (red line).

#### Interaction induced pinwheel crystals

**Figure 7:**
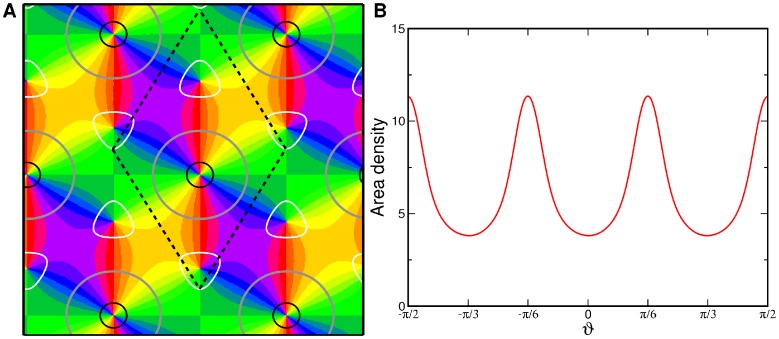
The Braitenberg pinwheel crystal, 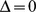 uniform solution of Eq. (53). **A** OP map, superimposed are the OD borders (gray), 90% ipsilateral eye dominance (black), and 90% contralateral eye dominance (white), 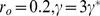. Dashed lines mark the unit cell of the regular pattern. **B** Distribution of preferred orientations.

The uniform solution Eq. (53), 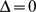 is illustrated in Fig. 7. This PWC contains only three pinwheels per unit cell leading to a pinwheel density of 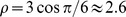. Two of the three pinwheels are located at maxima of the OD map (contra peak) while the remaining pinwheel is located at the minimum (ipsi peak) of the OD map. A remarkable property of this solution is that the pinwheel located at the OD minimum, carries a topological charge of 1 such that each orientation is represented twice around this pinwheel. Pinwheels of this kind have not yet been observed in experimentally recorded OP maps. This kind of uniform solution corresponds to the structural pinwheel model by Braitenberg [92]. We therefore refer to this solution as the *Braitenberg pinwheel crystal*.

The iso-orientation lines are again perfectly perpendicular to OD borders and this is independent of the bias 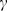. The solution is symmetric under a combined rotation by 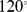 and an orientation shift by 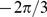. Further it is symmetric under a rotation by 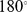. The pattern is selective to all orientations but the distribution of represented orientations is not uniform. The three orientations 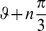, 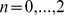 are overrepresented, see Fig. 7**B**.

Overall this OP map is dominated by uniform regions around hyperbolic points. In contrast to the ipsi center PWC all pinwheels in this OP map organize a roughly similar fraction of the cortical surface.

## Discussion

### Summary of results

In this study we presented a symmetry-based analysis of models formalizing that visual cortical architecture is shaped by the coordinated optimization of different functional maps. In particular, we focused on the question of whether and how different optimization principles specifically impact on the spatial layout of functional columns in the primary visual cortex. We identified different representative candidate optimization principles. We developed a dynamical systems approach for analyzing the simultaneous optimization of interacting maps and examined how their layout is influenced by coordinated optimization. In particular, we found that inter-map coupling can stabilize pinwheel-rich layouts even if pinwheels are intrinsically unstable in the weak coupling limit. We calculated and analyzed the stability properties of solutions forming spatially regular layouts with pinwheels arranged in a crystalline array. We analyzed the structure of these pinwheel crystals in terms of their stability properties, spatial layout, and geometric inter-map relationships. For all models, we calculated phase diagrams showing the stability of the pinwheel crystals depending on the OD bias and the inter-map coupling strength. Although differing in detail and exhibiting distinct pinwheel crystal phases for strong coupling, the phase diagrams exhibited many commonalities in their structure. These include the general fact that the hexagonal PWC phase is preceded by a phase of rhombic PWCs and that the range of OD biases over which pinwheel crystallization occurs is confined to the stability region of OD patch solutions.

### Comparison to previous work

Our analytical calculations of attractor and ground states close a fundamental gap in the theory of visual cortical architecture and its development. They rigorously establish that models of interacting OP and OD maps in principle offer a solution to the problem of pinwheel stability [51], [71]. This problem and other aspects of the influence of OD segregation on OP maps have previously been studied in a series of models such as elastic net models [34], [37], [41], [42], [51], [59], self-organizing map models [38], [40], [44], [49], [50], spin-like Hamiltonian models [58], [93], spectral filter models [94], correlation based models [95], [96], and evolving field models [97]. Several of these simulation studies found a higher number of pinwheels per hypercolumn if the OP map is influenced by strong OD segregation compared to the OP layout in isolation or the influence of weak OD segregation [51], [93], [97]. In such models, large gradients of OP and OD avoid each other [40], [49]. As a result, pinwheel centers tend to be located at centers of OD columns as seen in experiments [19], [45], [46], [48], [95]. By this mechanism, pinwheels are spatially trapped and pinwheel annihilation can be reduced [51]. Moreover, many models appear capable of reproducing realistic geometric inter-map relationships such as perpendicular intersection angles between OD borders and iso-orientation lines [59], [94], [95]. Tanaka et al. reported from numerical simulations that the relative positioning of orientation pinwheels and OD columns was dependent on model parameters [95]. Informative as they were, almost all of these previous studies entirely relied on simulation methodologies that do not easily permit to assess the progress and convergence of solutions. Whether the reported patterns were attractors or just snapshots of transient states and whether the solutions would further develop towards pinwheel-free solutions or other states thus remained unclear. Moreover, in almost all previous models, a continuous variation of the inter-map coupling strength was not possible which makes it hard to disentangle the contribution of inter-map interactions from intrinsic mechanisms. The only prior simulation study of a coordinated optimization model that tracked the number of pinwheels during the optimization process did not provide evidence that pinwheel annihilation could be stopped but only reported a modest reduction in annihilation efficiency [51]. From this perspective, the prior evidence for coordination induced pinwheel stabilization appears relatively limited. Our analytical results leave no room to doubt that map interactions can stabilize an intrinsically unstable pinwheel dynamics. They also reveal that interaction of orientation preference with a stripe pattern of OD is per se not capable of stabilizing pinwheels.

### The mathematical structure of interaction models

Independent of its predictions, our study clarifies the general mathematical structure of interaction dominated optimization models. To the best of our knowledge our study for the first time describes an analytical approach for examining the solutions of coordinated optimization models for OP and OD maps. Our symmetry-based phenomenological analysis of conceivable coupling terms provides a general classification and parametrization of biologically plausible coupling terms. To achieve this we mapped the optimization problem to a dynamical systems problem which allows for a perturbation expansion of fixed points, local minima, and optima. Using weakly nonlinear analysis, we derived amplitude equations as an approximate description near the symmetry breaking transition. We identified a limit in which inter-map coupling becomes effectively unidirectional enabling the use of the uncoupled OD patterns. We studied fixed points and calculated their stability properties for different types of inter-map coupling energies. This analysis revealed a fundamental difference between high and low order coupling energies. For the low order versions of these energies, a strong inter-map coupling typically leads to OP map suppression, causing the orientation selectivity of all neurons to vanish. In contrast, the higher order variants of the coupling energies do generally not cause map suppression but only influence pattern selection, see Text S1. We did not consider an interaction with the retinotopic map. Experimental results on geometric relationships between the retinotopic map and the OP map are ambiguous. In case of ferret visual cortex high gradient regions of both maps avoid each other [42]. In case of cat, however, high gradient regions overlap [18]. Such positive correlations cannot be easily treated with dimension reduction models, see [98]. It is noteworthy that our phenomenological analysis identified coupling terms that could induce an attraction of high gradient regions. Such terms contain the gradient of only one field and can thus be considered as a mixture of the gradient and the product-type energy.

### Conditions for pinwheel stabilization

Our results indicate that a patchy layout of a second visual map interacting with the OP map is important for the effectiveness of pinwheel stabilization by inter-map coupling. Such a patchy layout can be easily induced by an asymmetry in the representation of the corresponding stimulus feature such as eye dominance or spatial frequency preference. In spatial frequency maps, for instance, low spatial frequency patches tend to form islands in a sea of high spatial frequency preference [45]. Also in cat visual cortex the observed OD layout is patchy [99]–[103]. In our model, the patchy layout results from the overall dominance of one eye. In this case, OD domains form a system of hexagonal patches rather than stripes enabling the capture and stabilization of pinwheels by inter-map coupling. The results from all previous models did not support the view that OD stripes are capable of stabilizing pinwheels [51], [93], [97]. Our analysis shows that OD stripes are indeed not able to stabilize pinwheels, a result that appears to be independent of the specific type of map interaction. In line with this, several other theoretical studies, using numerical simulations [51], [93], [97], indicated that more banded OD patterns lead to less pinwheel rich OP maps. For instance, in simulations using an elastic net model, the average pinwheel density of OP maps interacting with a patchy OD layout was reported substantially higher (about 2.5 pinwheels per hypercolumn) than for OP maps interacting with a more stripe-like OD layout (about 2 pinwheels per hypercolumn) [51].

### Experimental evidence for pinwheel stabilization by inter-map coupling

**Figure 8:**
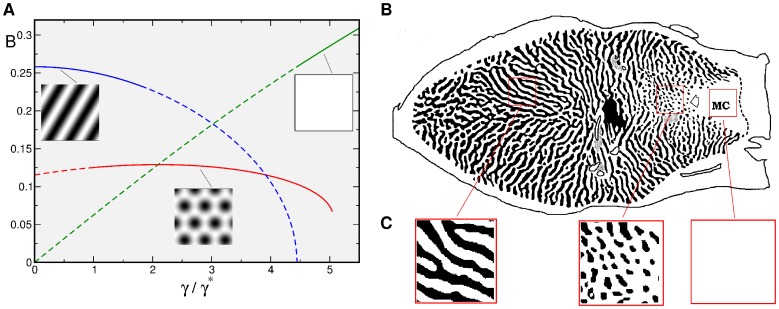
OD patterns. **A** Stationary amplitudes of the OD dynamics. The course of 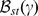 Eq. (89) (blue), 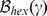 Eq. (90) (red), and of 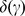 Eq. (68) (green) for 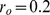. The solutions are plotted in solid lines within their stability ranges. **B** OD map of macaque monkey. Adapted from [106]. **C** Details of **B** with stripe-like, patchy, and homogeneous layout.

Several lines of biological evidence appear to support the picture of interaction induced pinwheel stabilization. Supporting the notion that pinwheels might be stabilized by the interaction with patchy OD columns, visual cortex is indeed dominated by one eye in early postnatal development and has a pronounced patchy layout of OD domains [104]–[106]. Further support for the potential relevance of this picture comes from experiments in which the OD map was artificially removed resulting apparently in a significantly smoother OP map [44]. In this context it is noteworthy that macaque visual cortex appears to exhibit all three fundamental solutions of our model for OD maps: stripes, hexagons, and a monocular solution, which are stable depending on the OD bias. In the visual cortex of macaque monkeys, all three types of patterns are found near the transition to the monocular segment, see [106] and Fig. (8). Here, OD domains form bands in the binocular region and a system of ipsilateral eye patches at the transition zone to the monocular region where the contralateral eye gradually becomes more dominant. If pinwheel stability depends on a geometric coupling to the system of OD columns one predicts systematic differences in pinwheel density between these three zones of macaque primary visual cortex. Because OD columns in the binocular region of macaque visual cortex are predominantly arranged in systems of OD stripes our analysis also indicates that pinwheels in these regions are either stabilized by other patchy columnar systems or intrinsically stable.

### The geometry of interaction induced pinwheel crystals

One important general observation from our results is that map organization was often not inferable by simple qualitative considerations on the energy functional. The organization of interaction induced hexagonal pinwheel crystals reveals that the relation between coupling energy and resulting map structure is quite complex and often counter intuitive. We analyzed the stationary patterns with respect to intersection angles and pinwheel positions. In all models, intersection angles of iso-orientation lines and OD borders have a tendency towards perpendicular angles whether the energy term mathematically depends on this angle, as for the gradient-type energies, or not, as for the product-type energies. Intersection angle statistics thus are not a very sensitive indicator of the type of interaction optimized. Mathematically, these phenomena result from the complex interplay between the single map energies and the interaction energies. In case of the low order gradient-type inter-map coupling energy all pinwheels are located at OD extrema, as expected from the used coupling energy. For other analyzed coupling energies, however, the remaining pinwheels are located either at OD saddle-points (low order product-type energy) or near OD borders (higher order gradient-type energy), in contrast to the expection that OD extrema should be energetically preferred. Remarkably, such correlations, which are expected from the gradient-type coupling energies, occur also in the case of the product-type energies. Remarkably, in case of product type energies pinwheels are located at OD saddle-points. which is not expected per se and presumably result from the periodic layout of OP and OD maps. Correlations between pinwheels and OD saddle-points have not yet been studied quantitatively in experiments and may thus provide valuable information on the principles shaping cortical functional architecture.

### How informative is map structure?

Our results demonstrate that, although distinct types of coupling energies can leave distinguishing signatures in the structure of maps shaped by interaction (as the OP map in our example), drawing precise conclusions about the coordinated optimization principle from observed map structures is not possible for the analyzed models. In the past numerous studies have attempted to identify signatures of coordinated optimization in the layout of visual cortical maps and to infer the validity of specific optimization models from aspects of their coordinated geometry [34], [37], [38], [40]–[42], [44], [49]–[51], [58], [59], [93]. It was, however, never clarified theoretically in which respect and to which degree map layout and geometrical factors of inter-map relations are informative with respect to an underlying optimization principle. Because our analysis provides complete information of the detailed relation between map geometry and optimization principle for the different models our results enable to critically assess whether different choices of energy functionals specifically impact on the predicted map structure and conversely what can be learned about the underlying optimization principle from observations of map structures.

We examined the impact of different interaction energies on the structure of local minima and ground states of models for the coordinated optimization of a complex and a real scalar feature map such as OP and OD maps. The models were constructed such that in the absence of interactions, the maps reorganized into simple stripe or blob pattern. In particular, the complex scalar map without interactions would form a periodic stripe pattern without any phase singularity. In all models, increasing the strength of interactions could eventually stabilize qualitatively different, more complex, and biologically more realistic patterns containing pinwheels that can become the energetic ground states for strong enough inter-map interactions. The way in which this happens provides fundamental insights into the relationships between map structure and energy functionals in optimization models for visual cortical functional architecture.

Our results demonstrate that the structure of maps shaped by inter-map interactions is in principle informative about the type of coupling energy. The organization of the complex scalar map that optimizes the joined energy functional was in general different for all different types of coupling terms examined. We identified a class of hPWC solutions which become stable for large inter-map coupling. This class depends on a single parameter which is specific to the used inter-map coupling energy. Furthermore, as shown in Text S1, pinwheel positions in rPWCs, tracked while increasing inter-map coupling strength, were different for different coupling terms examined and thus could in principle serve as a trace of the underlying optimization principle. This demonstrates that, although pinwheel stabilization is not restricted to a particular choice of the interaction term, each analyzed phase diagram is specific to the used coupling energy. In particular, in the strong coupling regime substantial information can be obtained from a detailed inspection of solutions.

In the case of the product-type coupling energies, the resulting phase diagrams are relatively complex as stationary solutions and stability borders depend on the magnitude of the OD bias. Here, even quantitative values of model parameters can in principle be constrained by analysis of the map layout. In contrast, for the gradient-type coupling energies, the bias dependence can be absorbed into the coupling strength and only selects the stationary OD pattern. This leads to relatively simple phase diagrams. For these models map layout is thus uninformative of quantitative model parameters. We identified several biologically very implausible OP patterns. In the case of the product-type energies, we found orientation scotoma solutions which are selective to only two preferred orientations. In the case of the low order gradient-type energy, we found OP patterns containing pinwheels with a topological charge of 1 which have not yet been observed in experiments. If the relevant terms in the coupling energy could be determined by other means, the parameter regions in which these patterns occur could be used to constrain model parameters by theoretical bounds.

The information provided by map structure overall appears qualitative rather than quantitative. In both low order inter-map coupling energies (and the gradient-type higher order coupling energy, see Text S1), hPWC patterns resulting from strong interactions were fixed, not exhibiting any substantial dependence on the precise choice of interaction coefficient. In principle, the spatial organization of stimulus preferences in a map is an infinite dimensional object that could sensitively depend in distinct ways to a large number of model parameters. It is thus not a trivial property that this structure often gives essentially no information about the value of coupling constants in our models. The situation, however, is reversed when considering the structure of rPWCs. These solutions exist and are stable although energetically not favored in the absence of inter-map interactions. Some of their pinwheel positions continuously depend on the strength of inter-map interactions. These solutions and their parameter dependence nevertheless are also largely uninformative about the nature of the interaction energy. This results from the fact that rPWCs are fundamentally uncoupled system solutions that are only modified by the inter-map interaction. As pointed out before, preferentially orthogonal intersection angles between iso-orientation lines and OD borders appear to be a general feature of coordinated optimization models in the strong coupling regime. Although the detailed form of the intersection angle histogram is solution and thus model specific, our analysis does not corroborate attempts to use this feature to support specific optimization principles, see also [52], [107], [108]. The stabilization of pinwheel crystals for strong inter-map coupling appears to be universal and provides per se no specific information about the underlying optimization principle. In fact, the general structure of the amplitude equations is universal and only the coupling coefficients change when changing the coupling energy. It is thus expected that also for other coupling energies, respecting the proposed set of symmetries, PWC solutions can become stable for large enough inter-map coupling.

### Conclusions

Our analysis conclusively demonstrates that OD segregation can stabilize pinwheels and induce pinwheel-rich optima in models for the coordinated optimization of OP and OD maps when pinwheels are intrinsically unstable in the uncoupled dynamics of the OP map. This allows to systematically assess the possibility that inter-map coupling might be the mechanism of pinwheel stabilization in the visual cortex. The analytical approach developed here is independent of details of specific optimization principles and thus allowed to systematically analyze how different optimization principles impact on map layout. Moreover, our analysis clarifies to which extend the observation of the layout in physiological maps can provide information about optimization principles shaping visual cortical organization.

The common design observed in experimental OP maps [3] is, however, not reproduced by the optima of the analyzed optimization principles. Whether this is a consequence of the applied weakly nonlinear analysis or of the low number of optimized feature maps or should be considered a generic feature of coordinated optimization models will be examined in part (II) of this study [91]. In part (II) we complement our analytical studies by numerical simulations of the full field dynamics. Such simulations allow to study the rearrangement of maps during the optimization process, to study the timescales on which optimization is expected to take place, and to lift many of the mathematical assumptions employed by the above analysis. In particular, we concentrate on the higher order inter-map coupling energies for which the derived amplitude equations involved several simplifying conditions, see Text S1.

## Methods

### Intersection angles

We studied the intersection angles between iso-orientation lines and OD borders. The intersection angle of an OD border with an iso-orientation contour 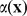 is given by
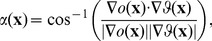
(63)where 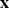 denotes the position of the OD zero-contour lines. A continuous expression for the OP gradient is given by 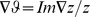. We calculated the frequency of intersection angles in the range 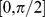. In this way those parts of the maps are emphasized from which the most significant information about the intersection angles can be obtained [19]. These are the regions where the OP gradient is high and thus every intersection angle receives a statistical weight according to 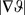. For an alternative method see [10].

### The transition from OD stripes to OD blobs

We studied how the emerging OD map depends on the overall eye dominance. To this end we mapped the uncoupled OD dynamics to a Swift-Hohenberg equation containing a quadratic interaction term instead of a constant bias. This allowed for the use of weakly nonlinear analysis to derive amplitude equations as an approximate description of the shifted OD dynamics near the bifurcation point. We identified the stationary solutions and studied their stability properties. Finally, we derived expressions for the fraction of contralateral eye dominance for the stable solutions.

#### Mapping to a dynamics with a quadratic term

Here we describe how to map the Swift-Hohenberg equation
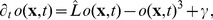
(64)to one with a quadratic interaction term. To eliminate the constant term we shift the field by a constant amount 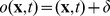. This changes the linear and nonlinear terms as
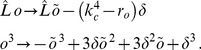
(65)We define the new parameters 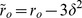 and 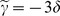. This leads to the new dynamics
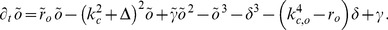
(66)The condition that the constant part is zero is thus given by
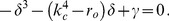
(67)For 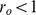 the real solution to Eq. (67) is given by
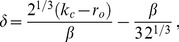
(68)with 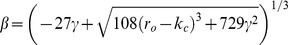. For small 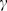 this formula is approximated as
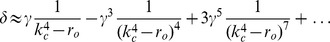
(69)The uncoupled OD dynamics we consider in the following is therefore given by
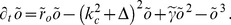
(70)This equation has been extensively studied in pattern formation literature [77].

#### Amplitude equations for OD patterns

We studied Eq. (70) using weakly nonlinear analysis. This method leads to amplitude equation as an approximate description of the full field dynamics Eq. (70) near the bifurcation point 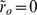. We summarize the derivation of the amplitude equations for the OD dynamics which is of the form
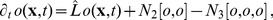
(71)with the linear operator 
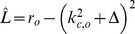
. In this section we use for simplicity the variables 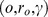 instead of 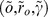. The derivation is performed for general quadratic and cubic nonlinearities but are specified later according to Eq. (9) as 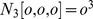 and 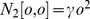. For the calculations in the following, it is useful to separate 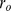 from the linear operator
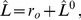
(72)therefore the largest eigenvalue of 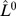 is zero. The amplitude of the field 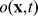 is assumed to be small near the onset 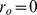 and thus the nonlinearities are small. We therefore expand both the field 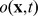 and the control parameter 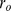 in powers of a small expansion parameter 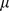 as
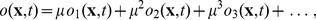
(73)and
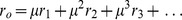
(74)The dynamics at the critical point 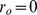 becomes arbitrarily slow since the intrinsic timescale 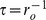 diverges at the critical point. To compensate we introduce a rescaled time scale 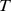 as
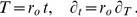
(75)In order for all terms in Eq. (71) to be of the same order the quadratic interaction term 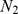 must be small. We therefore rescale 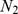 as 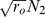. This preserves the nature of the bifurcation compared to the case 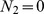.

We insert the expansion Eq. (73) and Eq. (74) in the dynamics Eq. (71) and get
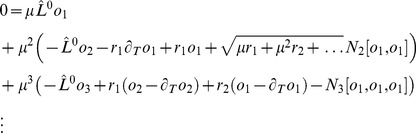
(76)We sort and collect all terms in order of their power in 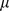. The equation can be fulfilled for 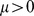 only if each of these terms is zero. We therefore solve the equation order by order. In the leading order we get the homogeneous equation
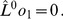
(77)Thus 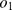 is an element of the kernel of 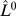. The kernel contains linear combinations of modes with wavevector 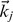 on the critical circle 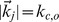. At this level any of such wavevectors is possible. We choose
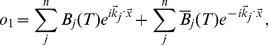
(78)where the wavevectors are chosen to be equally spaced 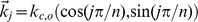 and the complex amplitudes 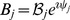. The homogeneous equation leaves the amplitudes 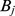 undetermined. These amplitudes are fixed by the higher order equations. Besides the leading order homogeneous equation we get inhomogeneous equations of the form
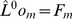
(79)To solve this inhomogeneous equation we first apply a solvability condition. We thus apply the *Fredholm Alternative theorem* to Eq. (79). Since the operator 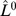 is self-adjoint 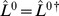, the equation is solvable if and only if 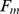 is orthogonal to the kernel of 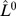 i.e.
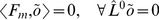
(80)The orthogonality to the kernel can be expressed by a projection operator 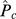 onto the kernel and the condition 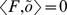 can be rewritten as 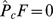.

At second order we get
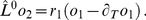
(81)Applying the solvability condition Eq. (80) we see that this equation can be fulfilled only for 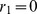. At third order we get
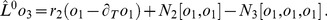
(82)The parameter 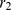 sets the scale in which 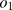 is measured and we can set 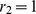. We apply the solvability condition and get
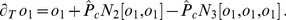
(83)We insert our ansatz Eq. (78) which leads to the amplitude equations at third order
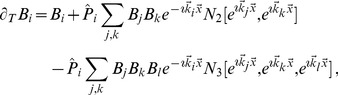
(84)where 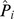 is the projection operator onto the subspace 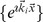 of the kernel. 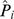 picks out all combinations of the modes which have their wavevector equal to 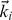. In our case the three active modes form a so called triad resonance 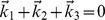. The quadratic coupling terms which are resonant to the mode 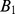 are therefore given by
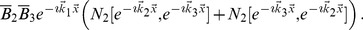
(85)Resonant contributions from the cubic nonlinearity result from terms of the form 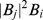. Their coupling coefficients are given by
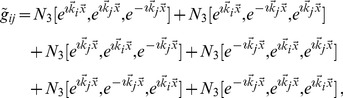
(86)and
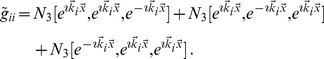
(87)When specifying the nonlinearities Eq. (9) the coupling coefficients are given by 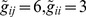. Finally, the amplitude equations (here in the shifted variables (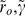) are given by
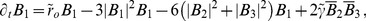
(88)where we scaled back to the original time variable 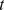. Equations for 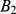 and 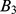 are given by cyclic permutation of the indices.

#### Stationary solutions

The amplitude equations (88) have three types of stationary solutions, namely OD stripes
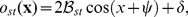
(89)with 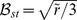, hexagons
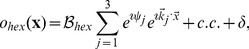
(90)with the resonance condition 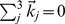 and 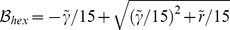. Finally, there is a homogeneous solution with spatially constant eye dominance
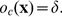
(91)The spatial average of all solutions is 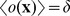. The course of 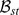, 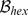, and of 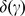 is shown in Fig. 8.

#### Linear stability analysis for OD patterns

We decomposed the amplitude equations (88) into the real and imaginary parts. From the imaginary part we get the phase equation
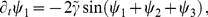
(92)and equations for 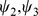 by cyclic permutation of the indices. The stationary phases are given by 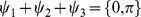. The phase equation can be derived from the potential 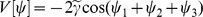. We see that the solution 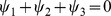 is stable for 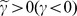 and the solution 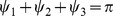 is stable for 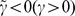.

**Figure 9:**
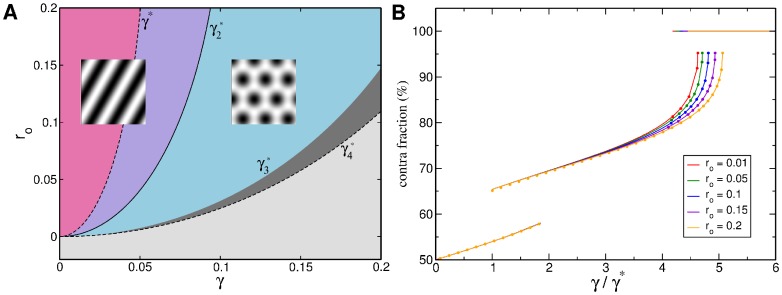
The uncoupled OD dynamics. **A** Phase diagram of the OD model Eq. (64). Dashed lines: stability border of hexagon solutions, solid line: stability border of stripe solution, gray regions: stability region of constant solution **B** Percentage of neurons dominated by the contralateral eye plotted for the three stationary solutions. Circles: numerically obtained values, solid lines: 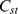 and 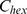.

We calculate the stability borders of the OD stripe, hexagon, and constant solution in the uncoupled case. This treatment follows [77]. In case of stripes the three modes of the amplitude equations are perturbed as
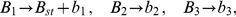
(93)assuming small perturbations 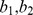, and 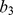. This leads to the linear equations 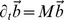 with the stability matrix
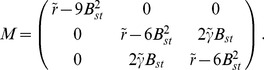
(94)The corresponding eigenvalues are given by
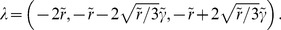
(95)This leads to the two borders for the stripe stability
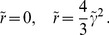
(96)In terms of the original variables 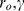 the borders are given by (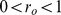)
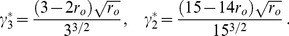
(97)To derive the stability borders for the hexagon solution 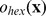 we perturb the amplitudes as
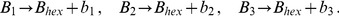
(98)The stability matrix is then given by
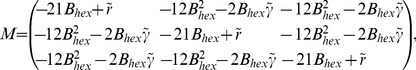
(99)and the corresponding eigenvalues are given by
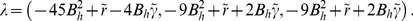
(100)The stability borders for the hexagon solution are given by
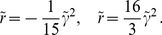
(101)In terms of the original variables we finally get
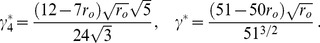
(102)The phase diagram of this model is depicted in Fig. 9**A**. It shows the stability borders 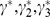, and 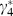 for the three solutions obtained by linear stability analysis. Without a bias term the OD map is either constant, for 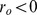, or has a stripe layout, for 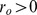. For positive 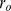 and increasing bias term there are two transition regions, first a transition region from stripes to hexagons and second a transition region from hexagons to the constant solution.

The spatial layout of the OD hexagons consists of hexagonal arrays of ipsilateral eye dominance blobs in a sea of contralateral eye dominance, see Fig. 9**A**.

#### Contralateral eye fraction

To compare the obtained solutions with physiological OD maps we quantified the fraction of neurons selective to the contralateral eye inputs. For stripe and hexagon solutions we thus calculated the fraction of contralateral eye dominated territory 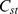 and 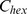. In case of stripes this is a purely one-dimensional problem. The zeros of the field are given by
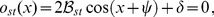
(103)with the solution
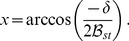
(104)As the field has a periodicity of 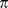 the area fraction is given by
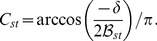
(105)In case of hexagons we observe that the territory of negative 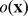 values is approximately a circular area. We obtain the fraction of negative 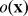 values by relating this area to the area of the whole hexagonal lattice. In case of hexagons the field is given by
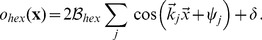
(106)As an approximation we project the field onto the 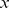-axis and choose for simplicity 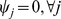. The field has its maximum at the origin 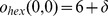. The projection leads to
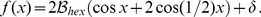
(107)The zeros 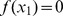 are located at
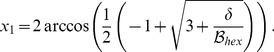
(108)The circular area of positive 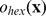 values is now given by 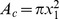. The periodicity of the hexagonal pattern is given by 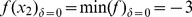. This leads to 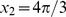. The area of the hexagon is therefore given by 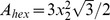. The contra fraction is finally given by
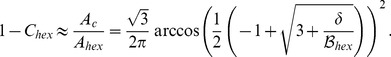
(109)The course of the fractions 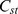 and 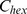 is shown in Fig. 9**B**. At the border 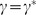, where hexagons become stable 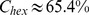. At the border 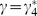, where hexagons loose stability 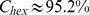. Both quantities are independent of 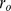. We confirmed our results by direct numerical calculation of the fraction of positive 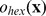 pixel values. Deviations from the result Eq. (109) are small. For 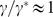 the zeros of Eq. (107) are not that well approximated with a circular shape and the projection described above leads to the small deviations which decrease with increasing bias 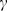.

## Supporting Information

Figure S1
**Stationary amplitudes with coupling energy** 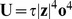 **.** Solid (dashed) lines: stable (unstable) solutions. **A,B** OD stripes, 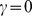 (blue), 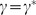 (green), 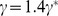 (red). **C,D** OD hexagons, 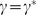 (blue), 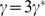 (red). **A,C** Transition from OP stripe solutions, **B,D** Transition from OP rhombic solutions. **E** Potential, Eq. (16), of OP stripes and OP rhombs interacting with OD stripes. **F** Potential, Eq. (16), of OP stripes, OP rhombs, and hPWC interacting with OD hexagons. Arrows indicate corresponding lines in the phase diagram, Fig. (S2).(TIF)

Figure S2
**A Phase diagram with coupling energy** 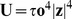 **,** 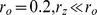 **.** Vertical black lines: stability range of OD stripes, hexagons, and constant solutions. Magenta (orange) line: Stability border of orientation scotoma stripes. Green solid line: Stability border of rhombic solutions. Red solid line: Stability border of PWC solutions, red dashed line: 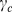, **B** Course of Eq. (27), dashed line: 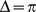. **C** Stability border between Eq. (27) solution and the 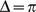 solution as a function of 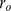 (vertical red line in **A**).(TIF)

Figure S3
**Bias dependent pinwheel crystals**, Eq. (27) **A** 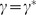, **B** 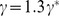, **C** 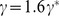, **D** 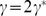 **.** OP map, superimposed are the OD borders (gray), 90% ipsilateral eye dominance (black), and 90% contralateral eye dominance (white), 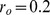. Dashed lines mark the unit cell of the regular pattern. **E,F** Distribution of orientation preference. **G** Intersection angles between iso-orientation lines and OD borders.(TIF)

Figure S4
**Stationary amplitudes with coupling energy** 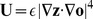, **A** Solid (dashed) lines: Stable (unstable) solutions. Blue: rPWC, green: distorted rPWC, red: hPWC. Black lines: stripe-like solutions. **B** Potential, Eq. (16), of OP stripes (black), OP rhombs (blue), and hPWC solutions (red). Arrows indicate corresponding lines in the phase diagram, Fig. (S5).(TIF)

Figure S5
**Phase diagram with coupling energy** 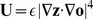, for 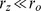 **.** Vertical lines: stability range of OD hexagons, green line: transition from rPWC to distorted rPWC, red line: stability border of hPWC, blue line: stability border of distorted rPWC. Above orange line: hPWC corresponds to ground state of energy.(TIF)

Figure S6
**Rhombic pinwheel crystals.** **A** OP map with superimposed OD borders (gray), 90% ipsilateral eye dominance (black), and 90% contralateral eye dominance (white), 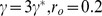. **B** Selectivity 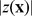, white: high selectivity, black: low selectivity.(TIF)

Figure S7
**Contra-center pinwheel crystals.** **A,B** OP map, superimposed are the OD borders (gray), 90% ipsilateral eye dominance (black), and 90% contralateral eye dominance (white), 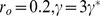. **A** 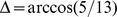, **B** 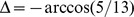. **C** Distribution of orientation preference. **D** OP map with superimposed OD map for three different values (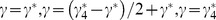) of the OD bias. **E** Selectivity 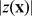, white: high selectivity, black: low selectivity. **F** Distribution of intersection angles.(TIF)

Figure S8
**Inter-map coupling strength dependent pinwheel positions.** OD map, superimposed pinwheel positions (points) for different inter-map coupling strengths, 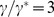. Numbers label pinwheels within the unit cell (dashed lines). Blue (green, red) points: pinwheel positions for rPWC (distorted rPWC, hPWC) solutions. **A** 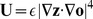, using stationary amplitudes from Fig. (S4)(a). Positions of distorted rPWCs move continuously (pinwheel 1,3,4). **B** 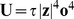, using stationary amplitudes from Fig. (S1). **D** Positions of rPWCs move continuously (pinwheel 5,6).(TIF)

Text S1 **Derivation of higher order amplitude equations and the analysis of optima for the higher order gradient-type and product-type inter-map coupling energies.**
(PDF)

Text S2
**Amplitude equations for the OP dynamics in case of the high order inter-map coupling energies** 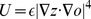 **and** 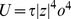 **.**
(PDF)

Text S3 **Stability matrices for the low order inter-map coupling energies.**
(PDF)
