# Supplementary figures and images for: Coordinated Optimization of Visual Cortical Maps (I) Symmetry-based Analysis
Source: PLoS Comput Biol. 2012 Nov 8;8(11):e1002466. doi: 10.1371/journal.pcbi.1002466 (PMC3493482; doi:10.1371/journal.pcbi.1002466)

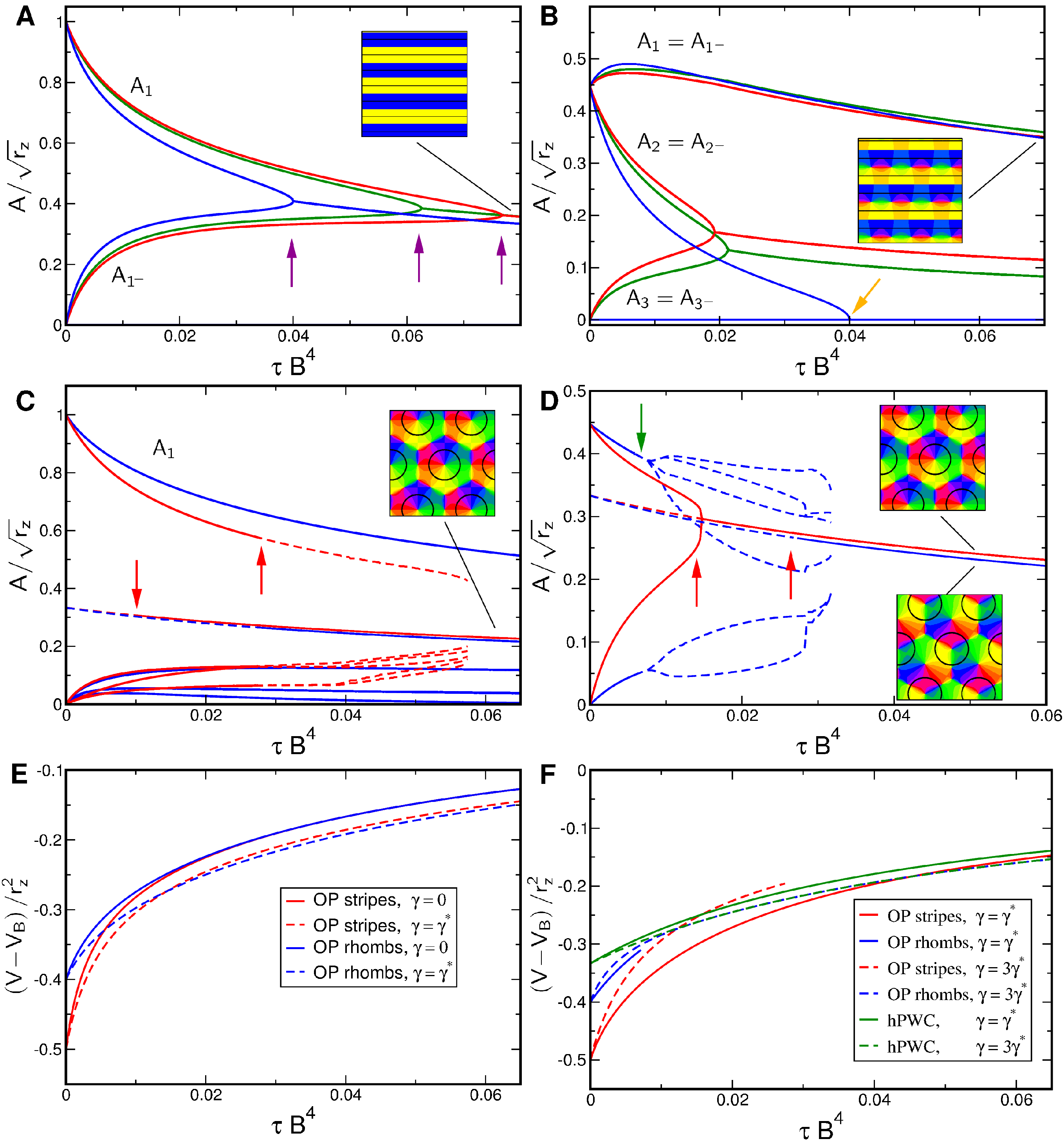

Supplement: Figure S1 — Stationary amplitudes with coupling energy . Solid (dashed) lines: stable (unstable) solutions. A,B OD stripes, (blue), (green), (red). C,D OD hexagons, (blue), (red). A,C Transition from OP stripe solutions, B,D Transition from OP rhombic solutions. E Potential, Eq. (16), of OP stripes and OP rhombs interacting with OD stripes. F Potential, Eq. (16), of OP stripes, OP rhombs, and hPWC interacting with OD hexagons. Arrows indicate corresponding lines in the phase diagram, Fig. (S2). (TIF) [file pcbi.1002466.s001.tif]

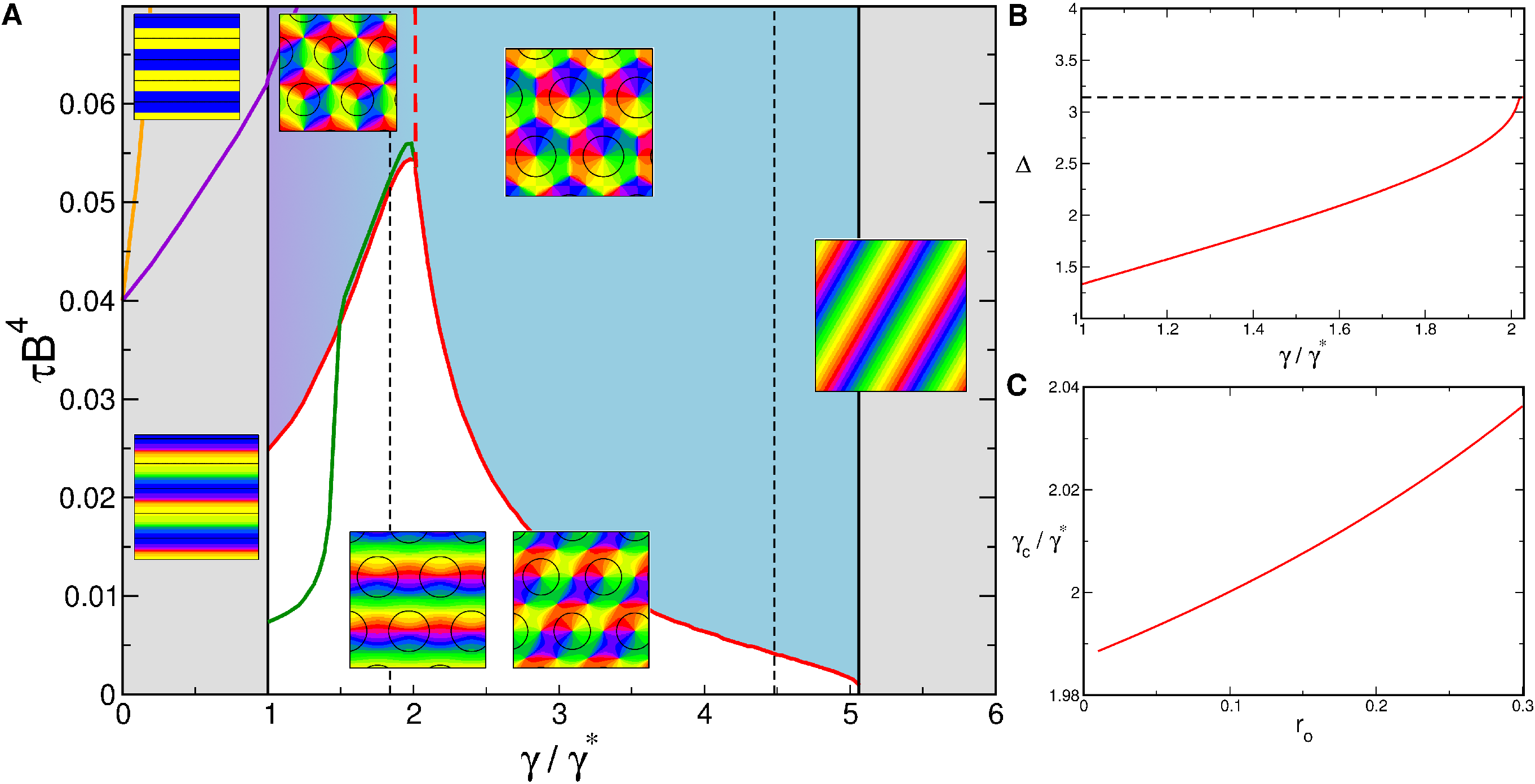

Supplement: Figure S2 — A Phase diagram with coupling energy , . Vertical black lines: stability range of OD stripes, hexagons, and constant solutions. Magenta (orange) line: Stability border of orientation scotoma stripes. Green solid line: Stability border of rhombic solutions. Red solid line: Stability border of PWC solutions, red dashed line: , B Course of Eq. (27), dashed line: . C Stability border between Eq. (27) solution and the solution as a function of (vertical red line in A). (TIF) [file pcbi.1002466.s002.tif]

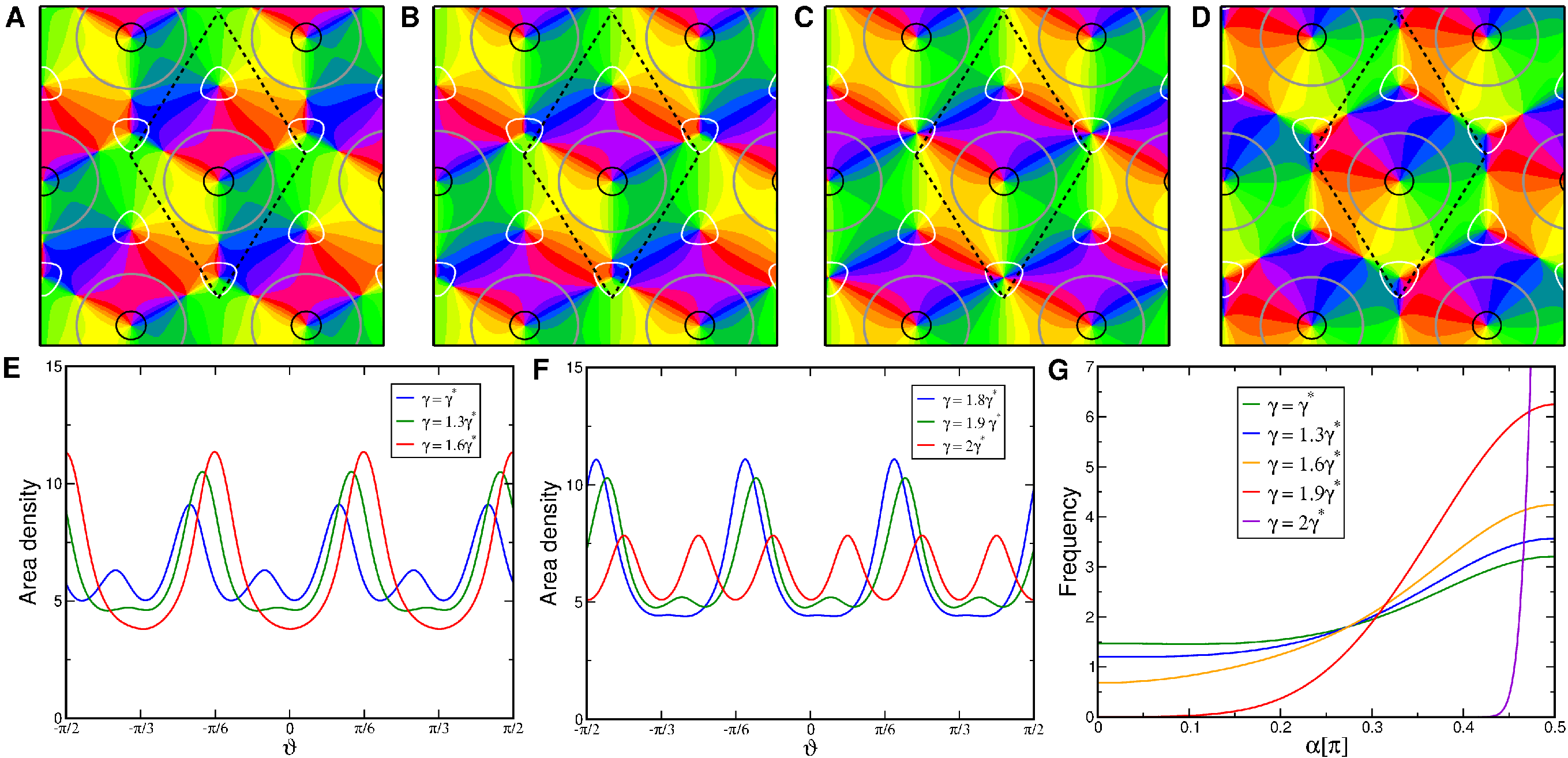

Supplement: Figure S3 — Bias dependent pinwheel crystals, Eq. (27) A , B , C , D . OP map, superimposed are the OD borders (gray), 90% ipsilateral eye dominance (black), and 90% contralateral eye dominance (white), . Dashed lines mark the unit cell of the regular pattern. E,F Distribution of orientation preference. G Intersection angles between iso-orientation lines and OD borders. (TIF) [file pcbi.1002466.s003.tif]

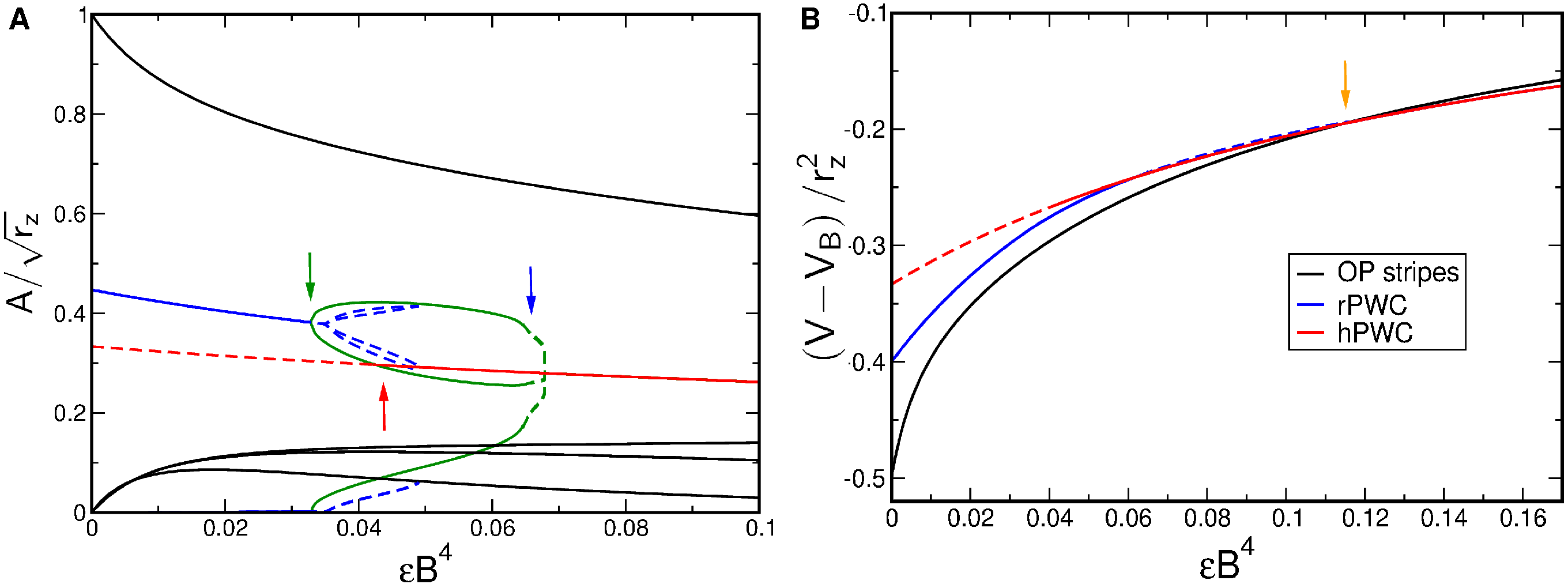

Supplement: Figure S4 — Stationary amplitudes with coupling energy , A Solid (dashed) lines: Stable (unstable) solutions. Blue: rPWC, green: distorted rPWC, red: hPWC. Black lines: stripe-like solutions. B Potential, Eq. (16), of OP stripes (black), OP rhombs (blue), and hPWC solutions (red). Arrows indicate corresponding lines in the phase diagram, Fig. (S5). (TIF) [file pcbi.1002466.s004.tif]

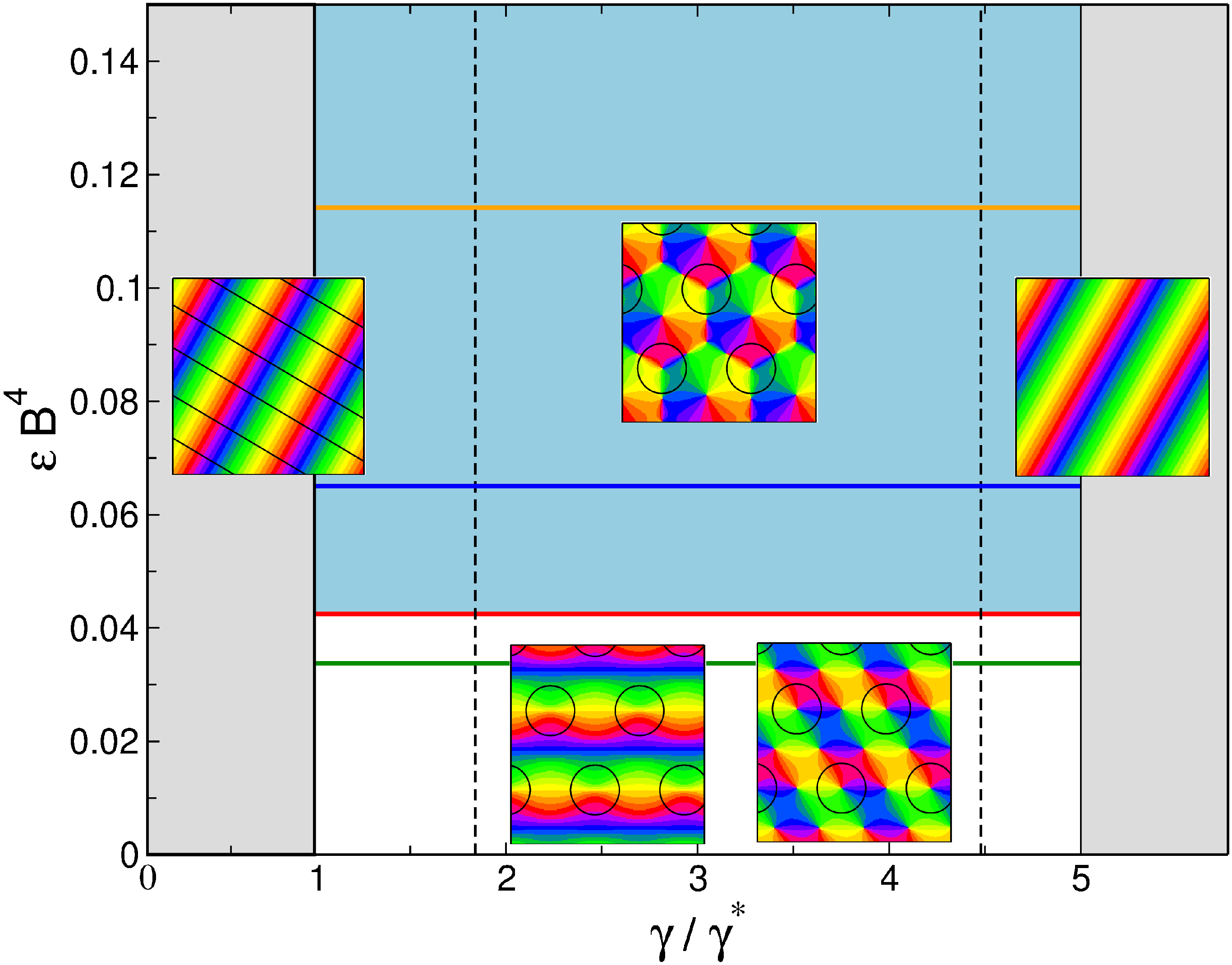

Supplement: Figure S5 — Phase diagram with coupling energy , for . Vertical lines: stability range of OD hexagons, green line: transition from rPWC to distorted rPWC, red line: stability border of hPWC, blue line: stability border of distorted rPWC. Above orange line: hPWC corresponds to ground state of energy. (TIF) [file pcbi.1002466.s005.tif]

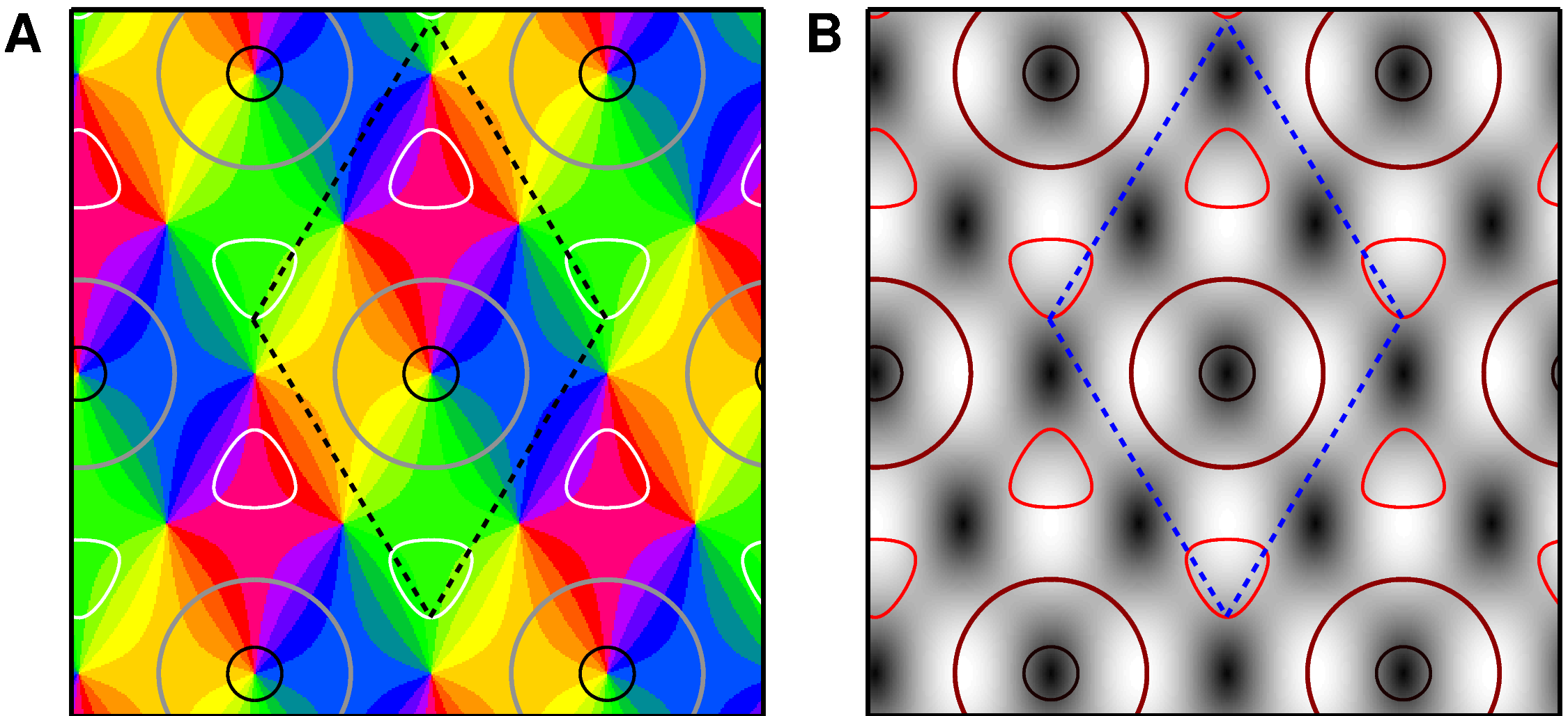

Supplement: Figure S6 — Rhombic pinwheel crystals. A OP map with superimposed OD borders (gray), 90% ipsilateral eye dominance (black), and 90% contralateral eye dominance (white), . B Selectivity , white: high selectivity, black: low selectivity. (TIF) [file pcbi.1002466.s006.tif]

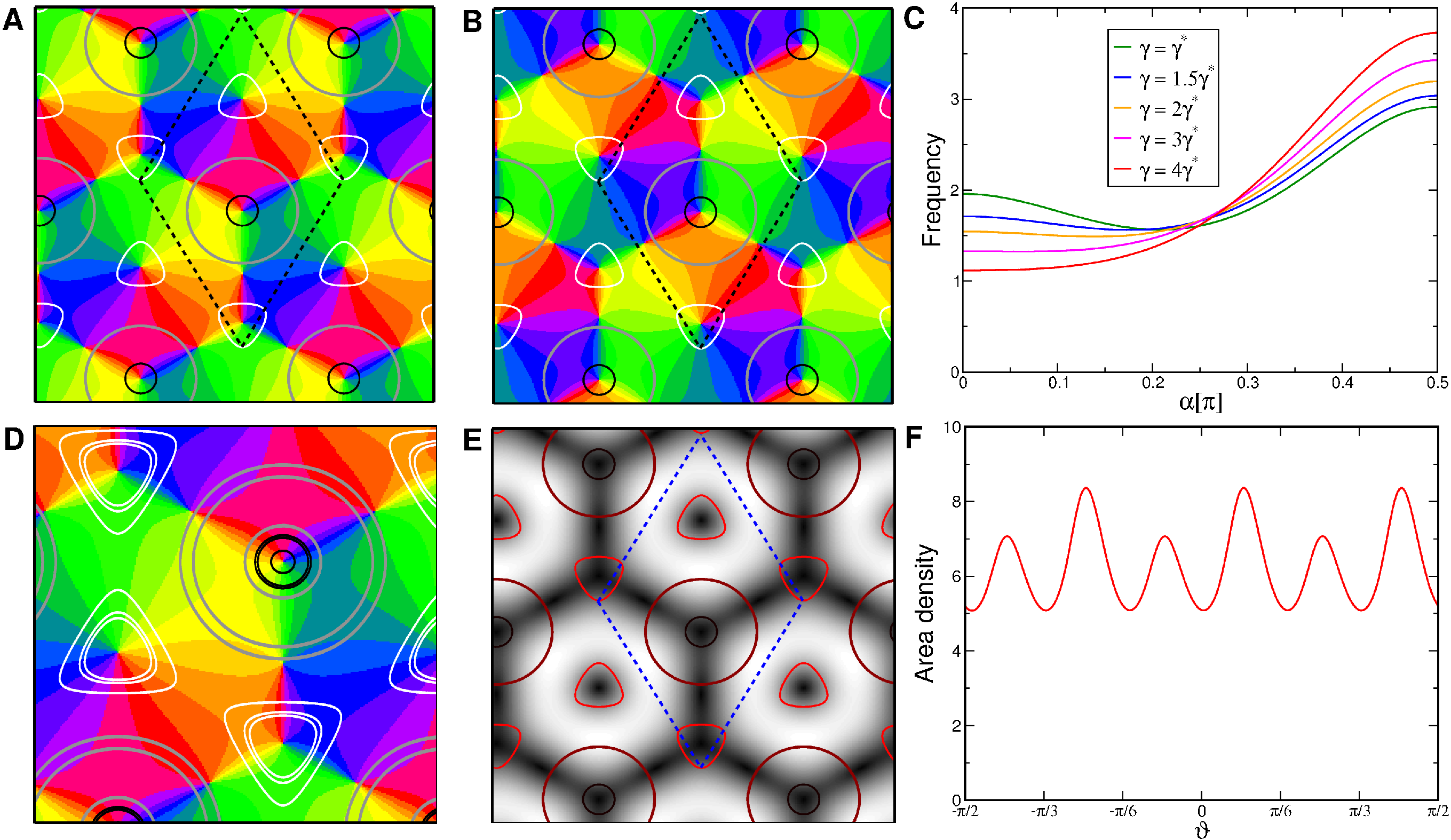

Supplement: Figure S7 — Contra-center pinwheel crystals. A,B OP map, superimposed are the OD borders (gray), 90% ipsilateral eye dominance (black), and 90% contralateral eye dominance (white), . A , B . C Distribution of orientation preference. D OP map with superimposed OD map for three different values () of the OD bias. E Selectivity , white: high selectivity, black: low selectivity. F Distribution of intersection angles. (TIF) [file pcbi.1002466.s007.tif]

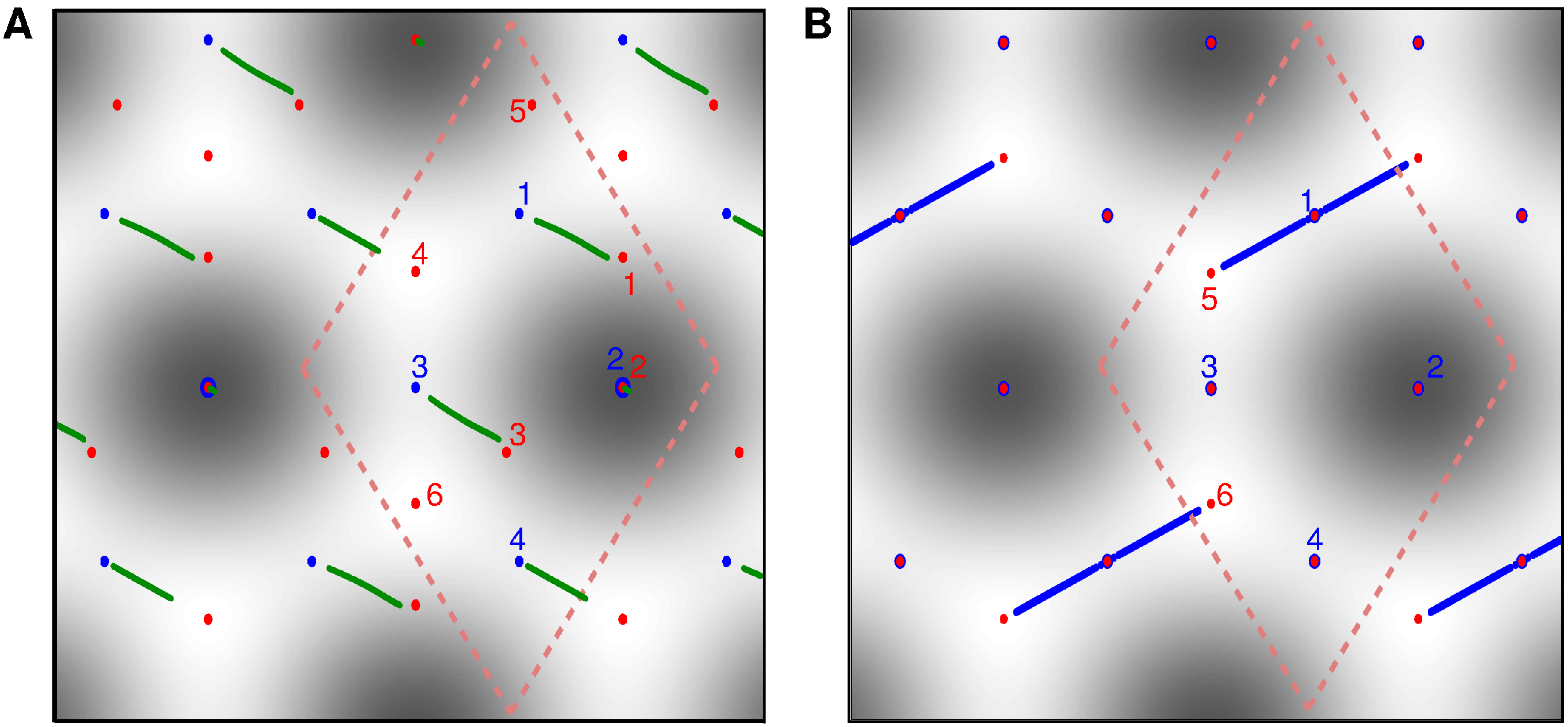

Supplement: Figure S8 — Inter-map coupling strength dependent pinwheel positions. OD map, superimposed pinwheel positions (points) for different inter-map coupling strengths, . Numbers label pinwheels within the unit cell (dashed lines). Blue (green, red) points: pinwheel positions for rPWC (distorted rPWC, hPWC) solutions. A , using stationary amplitudes from Fig. (S4)(a). Positions of distorted rPWCs move continuously (pinwheel 1,3,4). B , using stationary amplitudes from Fig. (S1). D Positions of rPWCs move continuously (pinwheel 5,6). (TIF) [file pcbi.1002466.s008.tif]
