# Supplementary material for: Coordinated Optimization of Visual Cortical Maps (I) Symmetry-based Analysis
Source: PLoS Comput Biol. 2012 Nov 8;8(11):e1002466. doi: 10.1371/journal.pcbi.1002466 (PMC3493482; doi:10.1371/journal.pcbi.1002466)
Supplement: Text S1 — Derivation of higher order amplitude equations and the analysis of optima for the higher order gradient-type and product-type inter-map coupling energies. (PDF) [file pcbi.1002466.s009.pdf]

Text S1: Supporting Information for

Coordinated optimization of visual cortical maps (I) Symmetry-based analysis

Introduction

In case of the low order inter-map coupling energies strong inter-map coupling leads to a suppression of OP selectivity. This suppressive effect can be avoided by restricting coupling strengths. One aim of this article is to test different optimization principles and potentially rule out some optimization principles. When comparing our results from different optimization principles to biological data such parameter tuning reduces the practicability. In this supporting information we complement our study using the high order inter-map coupling energies. We show that in this case a suppression of OP selectivity cannot occur. We derive coupled amplitude equations which, however, involve several mathematical assumptions. A systematic treatment as it is shown in the main article would imply that low order and higher order inter-map coupling energies are in general non-zero. Low order energy terms would enter at third order in the expansion and higher order corrections could potentially alter the stability properties. In addition, higher order inter-map coupling energies can affect the stability of patterns. In the following, we assume all low order inter-map coupling energies to be zero and that contributions entering the amplitude equations at higher orders can be neglected. The obtained results are confirmed numerically in part (II) of this study.

Coupled amplitude equations: Higher order terms

We studied the coupled Swift-Hohenberg equations

$$\begin{aligned} \partial_t\, z(\mathbf{x},t) &= r_z z(\mathbf{x},t) - \hat{L}_z^0\, z(\mathbf{x},t) - N_{3,u}[z,z,\overline{z}] - N_{7,c}[z,z,\overline{z},o,o,o,o] \\ \partial_t\, o(\mathbf{x},t) &= r_o o(\mathbf{x},t) - \hat{L}_o^0\, o(\mathbf{x},t) + N_{2,u}[o,o] - N_{3,u}[o,o,o] - \tilde{N}_{7,c}[o,o,o,z,z,\overline{z},\overline{z}]\,, \end{aligned} \qquad (1)$$

with the higher order inter-map coupling energies

$$U = \tau\, o^4 |z|^4 + \epsilon\, |\nabla z \cdot \nabla o|^4\,, \qquad (2)$$

using weakly nonlinear analysis. We study Eq. (1) close to the pattern forming bifurcation where r_z and r_o are small. We therefore expand both control parameters in powers of the small expansion parameter μ

$$\begin{aligned} r_z &= \mu r_{z1} + \mu^2 r_{z2} + \mu^3 r_{z3} + \ldots \\ r_o &= \mu r_{o1} + \mu^2 r_{o2} + \mu^3 r_{o3} + \ldots . \end{aligned} \tag{3}$$

Close to the bifurcation the fields are small and thus nonlinearities are weak. We therefore expand both fields as

$$\begin{aligned} o(\mathbf{x},t) = \mu o_1(\mathbf{x},t) + \mu^2 o_2(\mathbf{x},t) + \mu^3 o_3(\mathbf{x},t) + \ldots \\ z(\mathbf{x},t) = \mu z_1(\mathbf{x},t) + \mu^2 z_2(\mathbf{x},t) + \mu^3 z_3(\mathbf{x},t) + \ldots \end{aligned} \tag{4}$$

We further introduced a common slow timescale $T = r_z t$ and insert the expansions in Eq. (1) and get

$$\begin{aligned} 0 &= \mu \hat{L}^0 z_1 \\ &+\mu^2 \left(-\hat{L}^0 z_2 + r_{z1} z_1 - r_{z1} \partial_T z_1 \right) \\ &+\mu^3 \left(-r_{z2} \partial_T z_1 + r_{z2} z_1 + r_{z1} z_2 - r_{z1} \partial_T z_2 - \hat{L}^0 z_3 - N_{3,u}[z_1, z_1, \overline{z}_1] \right) \\ &\vdots \\ &+\mu^7 \left(-\hat{L}^0 z_7 + r_{z2} z_5 + r_{z4} z3 + r_{z6} z_1 + \cdots + N_{3,u}[z_5, z_1, \overline{z}_1] \right) \\ &+\mu^7 \left(-N_{7,c}[z_1, z_1, \overline{z}_1, o_1, o_1, o_1, o_1] \right) \\ &\vdots \end{aligned} \tag{5}$$

and

$$
\begin{aligned}
0 &= \mu\hat{L}^0 o_1 \\
&+\mu^2\left(-\hat{L}^0 o_2 + r_{o1}o_1 - r_{z1}\partial_T o_1 + \sqrt{\mu r_{o1} + \mu^2 r_{o2} + \ldots}\tilde{N}_{2,u}[o_1,o_1]\right) \\
&+\mu^3\left(-r_{z2}\partial_T o_1 + r_{o2}o_1 + r_{o1}o_2 - r_{z1}\partial_T o_2 - \hat{L}^0 o_3 - \tilde{N}_{3,u}[o_1,o_1,o_1]\right) \\
&\vdots \\
&+\mu^7\left(-\hat{L}^0 o_7 + r_{o2}o_5 + r_{o4}o_3 + r_{o6}o_1 + \cdots - \tilde{N}_{3,u}[o_5,o_1,o_1] - \tilde{N}_{2,u}[o_1,o_5] - \ldots\right) \\
&+\mu^7\left(-\tilde{N}_{7,c}[o_1,o_1,o_1,z_1,z_1,\overline{z}_1,\overline{z}_1]\right) \\
&\vdots
\end{aligned}
\tag{6}
$$

We consider amplitude equations up to seventh order as this is the order where the nonlinearity of the higher order coupling energy enters first. For Eq. (5) and Eq. (6) to be fulfilled each individual order in μ has to be zero. At linear order in μ we get the two homogeneous equations

$$
\hat{L}_z^0 z_1 = 0\,, \quad \hat{L}_o^0 o_1 = 0\,. \tag{7}
$$

Thus z_1 and o_1 are elements of the kernel of $\hat{L}_z^0$ and $\hat{L}_o^0$. Both kernels contain linear combinations of modes with a wavevector on the critical circle i.e.

$$
\begin{aligned}
z_1(\mathbf{x},T) &= \sum_j^n \left(A_j^{(1)}(T)e^{\imath\vec{k}_j\cdot\vec{x}} + A_{j^-}^{(1)}(T)e^{-\imath\vec{k}_j\cdot\vec{x}}\right) \\
o_1(\mathbf{x},T) &= \sum_j^n \left(B_j^{(1)}(T)e^{\imath\vec{k}'_j\cdot\vec{x}} + \overline{B}_j^{(1)}(T)e^{-\imath\vec{k}'_j\cdot\vec{x}}\right),
\end{aligned}
\tag{8}
$$

with the complex amplitudes $A_j^{(1)} = \mathcal{A}_j e^{\imath\phi_j}$, $B_j^{(1)} = \mathcal{B}_j e^{\imath\psi_j}$ and $\vec{k}_j = k_{c,z}\left(\cos(j\pi/n), \sin(j\pi/n)\right)$, $\vec{k}'_j = k_{c,o}\left(\cos(j\pi/n), \sin(j\pi/n)\right)$. In view of the hexagonal or stripe layout of the OD pattern shown in Fig. 1, $n = 3$ is an appropriate choice. In the following sections we assume $k_{c,o} = k_{c,z} = k_c$ i.e. the Fourier components of the emerging pattern are located on a common circle. To account for species differences we also analyzed models with detuned OP and OD wavelengths in part (II) of this study.

At second order in μ we get

$$\begin{aligned} \hat{L}^0 z_2 + r_{z1} z_1 - r_{z1} \partial_T z_1 &= 0 \\ \hat{L}^0 o_2 + r_{o1} o_1 - r_{z1} \partial_T o_1 &= 0\,. \end{aligned} \tag{9}$$

As z_1 and o_1 are elements of the kernel $r_{z1} = r_{o1} = 0$. At third order, when applying the solvability condition (see Methods), we get

$$\begin{aligned} r_{z2} \partial_T\, z_1 &= r_{z2} z_1 - \hat{P}_c N_{3,u}[z_1, z_1, \overline{z}_1] \\ r_{z2} \partial_T\, o_1 &= r_{o2} o_1 - \sqrt{r_{o2}}\, \hat{P}_c \tilde{N}_{2,u}[o_1, o_1] - \hat{P}_c \tilde{N}_{3,u}[o_1, o_1, o_1]\,. \end{aligned} \tag{10}$$

We insert the leading order fields Eq. (8) and obtain the amplitude equations

$$\begin{aligned} r_{z2} \partial_T A_i^{(1)} &= r_{z2} A_i^{(1)} - \sum_j g_{ij} |A_j^{(1)}|^2 A_i^{(1)} - \sum_j f_{ij} A_j^{(1)} A_{j^-}^{(1)} \overline{A}_{i^-}^{(1)} \\ r_{z2} \partial_T B_i^{(1)} &= r_{o2} B_i^{(1)} - 2\sqrt{r_{o2}}\, \overline{B}_{i+1}^{(1)} \overline{B}_{i+2}^{(1)} - \sum_j \widetilde{g}_{ij} |B_j^{(1)}|^2 B_i^{(1)}\,. \end{aligned} \tag{11}$$

These uncoupled amplitude equations obtain corrections at higher order. There are fifth order, seventh order and even higher order corrections to the uncoupled amplitude equations. In addition, at seventh order enters the nonlinearity of the higher order inter-map coupling energies. The amplitude equations up to seventh order are thus derived from

$$\begin{aligned} r_{z2} \partial_T\, z_1 &= r_{z2} z_1 - \hat{P}_c N_{3,u}[z_1, z_1, \overline{z}_1] \\ r_{z2} \partial_T\, z_3 &= r_{z2} z_3 - \cdots - \hat{P} N_{3,u}[z_1, z_1, \overline{z_3}] \\ r_{z2} \partial_T\, z_5 &= r_{z2} z_5 - \cdots - \hat{P}_c N_{3,u}[z_3, z_1, \overline{z_3}] - \hat{P}_c N_{7,c}[z_1, z_1, \overline{z}_1, o_1, o_1, o_1, o_1]\,, \end{aligned} \tag{12}$$

and corresponding equations for the fields o_1, o_3, and o_5. The field z_1 is given in Eq. (8) and its amplitudes $A^{(1)}$ and $B^{(1)}$ are determined at third order. The field z_3 contains contributions from modes off the critical circle $z_{3,off}$, $|\vec{k}_{off}| \neq k_c$ and on the critical circle i.e. $z_3 = z_{3,off} + \sum_j^n \left(A_j^{(3)}(T) e^{\imath \vec{k}_j \cdot \vec{x}} + A_{j^-}^{(3)}(T) e^{-\imath \vec{k}_j \cdot \vec{x}} \right)$. Its amplitude $A^{(3)}$ are determined at fifth order. The field z_5 also contains contributions from modes off the critical circle $z_{5,off}$ and on the critical circle i.e. $z_5 = z_{5,off} + \sum_j^n \left(A_j^{(5)}(T) e^{\imath \vec{k}_j \cdot \vec{x}} + A_{j^-}^{(5)}(T) e^{-\imath \vec{k}_j \cdot \vec{x}} \right)$.

Its amplitude $A^{(5)}$ are determined at seventh order. This leads to a series of amplitude equations

$$\begin{aligned}
r_{z2}\partial_T A_i^{(1)} &= r_{z2}A_i^{(1)} - \sum_j g_{ij}|A_j^{(1)}|^2 A_i^{(1)} - \sum_j f_{ij} A_j^{(1)} A_{j^-}^{(1)} \overline{A}_{i^-}^{(1)} \\
r_{z2}\partial_T A_i^{(3)} &= r_{z2}A_i^{(3)} - \cdots - \sum_j g_{ij}|A_j^{(1)}|^2 \overline{A}_i^{(3)} \\
r_{z2}\partial_T A_i^{(5)} &= r_{z2}A_i^{(5)} - \cdots - \sum_j g_{ij}|A_j^{(3)}|^2 A_i^{(1)} - \sum_{jlk} h_{ijlk}|A_j^{(1)}|^2|B_l^{(1)}|^2|B_k^{(1)}|^2 A_i^{(1)}\,,
\end{aligned} \tag{13}$$

which are solved order by order. We set $r_{z2} = r_z$ and $r_{o2} = r_o$ and rescale to the fast time. This leads to

$$\begin{aligned}
\partial_t A_i^{(1)} &= r_z A_i^{(1)} - \sum_j g_{ij}|A_j^{(1)}|^2 A_i^{(1)} - \sum_j f_{ij} A_j^{(1)} A_{j^-}^{(1)} \overline{A}_{i^-}^{(1)} \\
\partial_t A_i^{(3)} &= r_z A_i^{(3)} - \cdots - \sum_j g_{ij}|A_j^{(1)}|^2 \overline{A}_i^{(3)} \\
\partial_t A_i^{(5)} &= r_z A_i^{(5)} - \cdots - \sum_j g_{ij}|A_j^{(3)}|^2 A_i^{(1)} - \sum_{jlk} h_{ijlk}|A_j^{(1)}|^2|B_l^{(1)}|^2|B_k^{(1)}|^2 A_i^{(1)}\,.
\end{aligned} \tag{14}$$

We can combine the amplitude equations up to seventh order by introducing the amplitudes $A_j = A_j^{(1)} + A_j^{(3)} + A_j^{(5)}$ and $B_j = B_j^{(1)} + B_j^{(3)} + B_j^{(5)}$. This leads to the amplitude equations

$$\begin{aligned}
\partial_t\, A_i &= r_z A_i - \sum_j g_{ij}|A_j|^2 A_i - \sum_j f_{ij} A_j A_{j^-} \overline{A}_{i^-} \\
&\quad - \sum_{jlk} h_{ijlk}|A_j|^2|B_l|^2|B_k|^2 A_i - \ldots \\
\partial_t\, B_i &= r_o B_i - 2\overline{B}_{i+1}\overline{B}_{i+2} - \sum_j \tilde{g}_{ij}|B_j|^2 A B_i \\
&\quad - \sum_{jlk} h_{ijlk}|B_j|^2|A_l|^2|A_k|^2 B_i - \ldots\,.
\end{aligned} \tag{15}$$

For simplicity we have written only the simplest inter-map coupling terms. Depending on the configuration of active modes additional contributions may enter the amplitude equations. In addition, for the product-type coupling energy, there are coupling terms which contain the constant δ, see Methods. In case of $A \ll B \ll 1$ the inter-map coupling terms in dynamics of the modes B are small. In this limit the dynamics of the modes B decouples from the modes A and we can use the uncoupled OD dynamics, see Methods. In the following, we use the effective inter-map coupling strength ϵB^4 (and τB^4).

Higher order inter-map coupling energies

Optima of particular optimization principles: Higher order coupling terms

In this article we demonstrated that the low order coupling terms can lead to a complete suppression of OP selectivity i.e. vanishing magnitude of the order parameter $|z|$. As the coupling terms are effectively linear they not only influence pattern selection but also whether there is a pattern at all. This is in general not the case for higher order coupling energies using the amplitude equations Eq. (15). In this case the coupling is an effective cubic interaction term and complete selectivity suppression is impossible. Moreover, as in the low-order energy case, we could identify the limit $r_z \ll r_o$ in which the backreaction onto the OD map formally becomes negligible. The potential is of the form

$$\begin{aligned} V &= V_A + V_B + \\ &+\sum_{j,l,k}\sum_{u,v,w,p} h^{ijlk}_{uvwp}\, A_j A_l \overline{A}_k \overline{A}_i B_u B_v B_w B_p\, \delta_{\vec{k}_j+\vec{k}_l-\vec{k}_k-\vec{k}_i+\vec{k}_u+\vec{k}_v+\vec{k}_w+\vec{k}_p,0} \\ &+\delta\sum_{j,l,k}\sum_{u,v,w} h^{ijlk}_{uvp}\, A_j A_l \overline{A}_k \overline{A}_i B_u B_v B_w\, \delta_{\vec{k}_j+\vec{k}_l-\vec{k}_k-\vec{k}_i+\vec{k}_u+\vec{k}_v+\vec{k}_w,0} \\ &+\delta^2\sum_{j,l,k}\sum_{u,v} h^{ijlk}_{uvp}\, A_j A_l \overline{A}_k \overline{A}_i B_u B_v\, \delta_{\vec{k}_j+\vec{k}_l-\vec{k}_k-\vec{k}_i+\vec{k}_u+\vec{k}_v,0} \\ &+\delta^3\sum_{j,l,k}\sum_{u} h^{ijlk}_{uvp}\, A_j A_l \overline{A}_k \overline{A}_i B_u\, \delta_{\vec{k}_j+\vec{k}_l-\vec{k}_k-\vec{k}_i+\vec{k}_u,0} \\ &+\delta^4\sum_{j,l,k} h^{ijlk}\, A_j A_l \overline{A}_k \overline{A}_i\, \delta_{\vec{k}_j+\vec{k}_l-\vec{k}_k-\vec{k}_i,0}\,, \end{aligned} \tag{16}$$

where $\delta_{i,j}$ denotes the Kronecker delta and the uncoupled contributions

$$\begin{aligned} V_A &= -r_z\sum_{j}^{3}|A_j|^2+\frac{1}{2}\sum_{i,j}^{3} g_{ij}|A_i|^2|A_j|^2+\frac{1}{2}\sum_{i,j}^{3} f_{ij}A_iA_{i^-}\overline{A}_j\overline{A}_{j^-} \\ V_B &= -r_o\sum_{j}^{3}|B_j|^2+\frac{1}{2}\sum_{i,j}^{3}\tilde{g}_{ij}|B_i|^2|B_j|^2\,. \end{aligned} \tag{17}$$

Amplitude equations can be derived from the potential by $\partial_t A_i = -\delta V/\delta \overline{A}_i$. We have not written terms involving the modes A_{j^-} or $\overline{B}_j$. The complete amplitude equations involving all modes and the corresponding coupling coefficients are given in Text S2. As for the low order coupling energies terms involving the constant δ depend only on the coupling coefficient of the product-type energy τ. In the fol-

lowing we specify the amplitude equations for negligible backreaction where $\mathcal{B} = \mathcal{B}_{hex}$, $\mathcal{B} = \mathcal{B}_{st}$, or $\mathcal{B} = 0$.

Product-type energy $U = \tau\, o^4 |z|^4$

First, we studied the higher order product-type inter-map coupling energy $U = \tau\, o^4 |z|^4$. As for the lower order version of this coupling energy the shift $\delta(\gamma)$ explicitly enters the amplitude equations resulting in a rather complex parameter dependence, see Eq. (68) in the Methods section.

Stationary solutions and their stability

In the case of OD stripes the amplitude equations of OP modes read

$$\begin{aligned}
\partial_t A_1 &= r_z A_1 - \sum_j \left(g^{(1)}_{1j} |A_j|^2 A_1 + g^{(2)}_{1j} |A_j|^2 A_{1^-} + g^{(3)}_{1j} A_j A_{j^-} \overline{A}_{1^-} + g^{(4)}_{1j} A_j A_{j^-} \overline{A}_1 \right) \\
&\quad - B^4 A_{1^-}^2 \overline{A}_1 - \sum_{u \neq v \neq w} A_u A_v \overline{A}_w \left(\left(8\delta^3 B + 24\delta B^2 \overline{B} \right) \delta_{\vec{k}_u + \vec{k}_v - \vec{k}_w, 0} \right. \qquad (18) \\
&\quad \left. + \left(8\delta^3 \overline{B} + 24\delta B \overline{B}^2 \right) \delta_{\vec{k}_u + \vec{k}_v - \vec{k}_w, 2\vec{k}_1} + 8\delta B^3 \delta_{\vec{k}_u + \vec{k}_v - \vec{k}_w, -2\vec{k}_1} \right) \\
\partial_t A_2 &= r_z A_2 - \sum_j \left(g^{(1)}_{2j} |A_j|^2 A_2 + g^{(3)}_{2j} A_j A_{j^-} \overline{A}_{2^-} \right) \\
&\quad - g^{(2)}_{ii} A_2 A_{1^-} \overline{A}_1 - g^{(5)} A_2 A_1 \overline{A}_{1^-} - 1/2 g^{(2)}_{ii} A_{1^-}^2 \overline{A}_{2^-} - 1/2 g^{(5)} A_1^2 \overline{A}_{2^-} \\
&\quad - \sum_{u,v,w} A_u A_v \overline{A}_w \left(g^{(6)}_{uv} \delta_{\vec{k}_u + \vec{k}_v - \vec{k}_w, \vec{k}_2} + g^{(7)}_{ij} \delta_{\vec{k}_u + \vec{k}_v - \vec{k}_w, \vec{k}_1 + \vec{k}_2} \right. \\
&\quad \left. + g^{(8)}_{ij} \delta_{\vec{k}_u + \vec{k}_v - \vec{k}_w, -\vec{k}_1 + \vec{k}_2} + g^{(9)}_{ij} \delta_{\vec{k}_u + \vec{k}_v - \vec{k}_w, 2\vec{k}_1 + \vec{k}_2} + g^{(10)}_{ij} \delta_{\vec{k}_u + \vec{k}_v - \vec{k}_w, -2\vec{k}_1 + \vec{k}_2} \right),
\end{aligned}$$

where $\delta_{i,j}$ denotes the Kronecker delta and

$$\begin{aligned}
g_{ii}^{(1)} &= 1+\delta^4+12\delta^2|B|^2+6|B|^4, & g_{ij\neq i}^{(1)} &= 2g_{ii}^{(1)},\\
g_{ii}^{(2)} &= g_{ij\neq i}^{(2)} = 12\delta^2 B^2+8B^3\overline{B}, & &\\
g_{ii}^{(3)} &= 0, & g_{ij\neq i}^{(3)} &= 2+12|B|^4+24\delta^2|B|^2+2\delta^4,\\
g_{ii}^{(4)} &= 0, & g_{ij\neq i}^{(4)} &= 12\delta^2 B^2+8B^3\overline{B},\\
g^{(5)} &= 12\delta^2\overline{B}^2+8B\overline{B}^3, & g_{uu}^{(6)} &= 6|B|^4+6\delta|B|^2,\; g_{uv\neq u}^{(6)} = 2g_{uu}^{(6)},\\
g_{uu}^{(7)} &= 4\overline{B}\delta^3+1B\overline{B}^2\delta, & g_{uv\neq u}^{(7)} &= 2g_{uu}^{(7)},\\
g_{uu}^{(8)} &= 4B\delta^3+1B^2\overline{B}\delta, & g_{uv\neq u}^{(8)} &= 2g_{uu}^{(8)},\\
g_{uu}^{(9)} &= 6\overline{B}^2\delta^2, & g_{uv\neq u}^{(9)} &= 2g_{uu}^{(9)},\\
g_{uu}^{(10)} &= 6B^2\delta^2, & g_{uv\neq u}^{(10)} &= 2g_{uu}^{(10)}.
\end{aligned}$$

The equation for the mode A_3 is given by interchanging the modes A_2 and A_3 in Eq. (18). The equations for the modes A_{i^-} are given by interchanging the modes A_i and A_{i^-} and interchanging the modes B_i and $\overline{B}_i$.

In this case, at low inter-map coupling the OP stripes given by

$$z = \mathcal{A}_1 e^{\imath\left(\vec{k}_1\cdot\vec{x}+\phi_1\right)} - \mathcal{A}_{1^-} e^{-\imath\left(\vec{k}_1\cdot\vec{x}+\phi_{1^-}\right)}\,, \tag{19}$$

with $\phi_1-\phi_{1^-} = 2\psi_1+\pi$ run parallel to the OD stripes. Their stationary amplitudes are given by

$$\begin{aligned}
A_1^2 &= \left(u-v-\sqrt{u^2-2uv+v^2-16w^2}\right)^2 x/32w\\
A_{1^-} &= x/2\,,
\end{aligned}\tag{20}$$

with $x = r_z\left(u-v+\sqrt{u^2-2uv+v^2-16w^2}\right)/(uv-v^2-8w^2)$, $u = 2+13\mathcal{B}^4\tau+24\mathcal{B}^2\delta^2\tau+2\delta^4\tau$, $v = (6\mathcal{B}^4+12\mathcal{B}^2\delta^2+\delta^4)\tau$, $w = (2\mathcal{B}^2+3\delta^2)\tau\mathcal{B}^2$. The parameter dependence of these stripe solutions is shown in Fig. S1**A**.

At large inter-map coupling the attractor states of the OP map consist of a stripe pattern containing only two preferred orientations, namely $\vartheta = \phi_1$ and $\vartheta = \phi_1+\pi/2$. The zero contour lines of the OD map are along the maximum amplitude of orientation preference minimizing the energy term.

In addition there are rhombic solutions

$$z = \mathcal{A}_1 e^{\imath(\vec{k}_1\cdot\vec{x}+\psi_1)} + \mathcal{A}_{1^-} e^{-\imath(\vec{k}_1\cdot\vec{x}-\psi_1+\pi)} + \mathcal{A}_2 e^{\imath(\vec{k}_2\cdot\vec{x}+\psi_1)} + \mathcal{A}_{2^-} e^{-\imath(\vec{k}_2\cdot\vec{x}-\psi_1)}\,, \tag{21}$$

which exist also in the uncoupled case, see Fig. S1**B**. However, these rhombic solutions are energetically not favored compared to stripe solutions, see Fig. S1**C**. The inclusion of the inter-map coupling makes these rhombic solution even more stripe-like.

In case of a OD constant solution the amplitude equations read

$$\partial_t A_i = r_z A_i - \sum_j g_{ij}|A_j|^2 A_i - \sum_j f_{ij} A_j A_{j^-} \overline{A}_{i^-}\,, \tag{22}$$

with $g_{ii} = 1 + \delta^4\tau$, $g_{ij} = 2 + 2\delta^4\tau$ and $f_{ij} = 2 + 2\delta^4\tau$. Inter-map coupling thus leads to a renormalization of the uncoupled interaction terms. Stationary solutions are stripes with the amplitude

$$\mathcal{A} = \sqrt{\frac{r_z}{1+\delta^4\tau}}\,, \tag{23}$$

and rhombic solutions with the stationary phases $\phi_1 + \phi_{1^-} - \phi_2 - \phi_{2^-} = \pi$ and the stationary amplitudes

$$\mathcal{A}_1 = \mathcal{A}_{1^-} = \mathcal{A}_2 = \mathcal{A}_{2^-} = \sqrt{r_z/(5+5\delta^4\tau)}\,. \tag{24}$$

In the case of OD hexagons we identify, in addition to stripe-like and rhombic solutions, uniform solutions $\mathcal{A}_i = \mathcal{A}$. When solving the amplitude equations numerically we have seen that the phase relations vary with the inter-map coupling strength τ for non-uniform solutions. But for the uniform solution the phase relations are independent of the inter-map coupling strength. We use the ansatz for uniform solutions

$$\begin{aligned} \mathcal{A}_j &= \mathcal{A}_{j^-} = \mathcal{A}, \quad j = 1,2,3 \\ \phi_j &= \psi_j + (j-1)2\pi/3 + \Delta\,\delta_{j,2} \\ \phi_{j^-} &= -\psi_j + (j-1)2\pi/3 + \Delta\,(\delta_{j,1} + \delta_{j,3})\,, \end{aligned} \tag{25}$$

where $\delta_{i,j}$ is the Kronecker delta and Δ a constant parameter. This leads to the stationarity condition

$$6\mathcal{A}^2\mathcal{B}\left[4\left(-4B^3+7B^2\delta-B\delta^2+\delta^3\right)+\mathcal{B}\cos\Delta\left(13B^2-8B\delta+6\delta^2\right)\right]\sin\Delta=0\,. \tag{26}$$

Four types of stationary solutions exist namely the $\Delta = 0, \Delta = \pi$, which we already observed in case of the low order energies, and the solutions

$$\Delta=\Delta(\gamma)=\pm\arccos\left(\frac{4(4\mathcal{B}^3-7\mathcal{B}^2\delta+\mathcal{B}\delta^2-\delta^3)}{\mathcal{B}(13\mathcal{B}^2-8\mathcal{B}\delta+6\delta^2)}\right)\,, \tag{27}$$

which depends on $\mathcal{B}$ and δ and thus on the bias γ. The course of Eq. (27) as a function of γ is shown in Fig. S2**B**. Stationary amplitudes for these solutions are given by

$$\begin{aligned}
\mathcal{A}^2_{\Delta=0} &= \frac{r_z}{3\tau\left(3/\tau+33\mathcal{B}^4+56\mathcal{B}^3\delta+50\mathcal{B}^2\delta^2+16\mathcal{B}\delta^3+3\delta^4\right)}\\
\mathcal{A}^2_{\Delta=\pi} &= \frac{r_z}{\tau\left(9/\tau+483\mathcal{B}^4-504\mathcal{B}^3\delta+246\mathcal{B}^2\delta^2-48\mathcal{B}\delta^3+9\delta^4\right)}\\
\mathcal{A}^2_{\Delta(\gamma)} &= \frac{r_z(13\mathcal{B}^2-8\mathcal{B}\delta+6\delta^2)}{3\tau\left(411\mathcal{B}^6\tau+704\mathcal{B}^5\delta\tau-376\mathcal{B}^4\delta^2\tau+32\mathcal{B}^3\delta^3\tau+2\delta^2(9-7\delta^4\tau)-39\mathcal{B}^2(3\delta^4\tau-1)+8\mathcal{B}\delta(5\delta^4\tau-3)\right)}
\end{aligned} \tag{28}$$

We study the stability properties of OP stripe-like, rhombic and uniform solutions using linear stability analysis. The eigenvalues of the stability matrix , see also Text S3, are calculated numerically. Linear stability analysis shows that for $\tau \geq 0$ the $\Delta = 0$ solution is unstable for all bias values. The stability region of the $\Delta = \pi$ solution and the solution Eq. (27) is bias dependent. The bias dependent solution Eq. (27) is stable for $\gamma > \gamma^*$ and $\gamma < \gamma_c$ for which $\Delta = \pi$, see Fig. S2**B**. For larger bias $\gamma > \gamma_c$ only the $d = \pi$ uniform solution is stable.

Bifurcation diagram

For increasing inter-map coupling strength the amplitudes of the OP solutions are shown in Fig. S1. In case of inter-map coupling strength dependent stationary phases, stationary solutions are calculated numerically using a Newton method and initial conditions close to these solutions. We followed the unstable solutions (dashed lines in Fig. S1) until this method did not converge anymore. Not shown are solutions which are unstable in general. The parameter dependence of OP solutions when interacting with OD stripes is shown in Fig. S1**A,B**. Similar to the low order variant of this coupling energy the

amplitude of the stripes pattern A_1 is suppressed while the amplitude of the opposite mode A_{1^-} grows. Finally both amplitudes collapse, leading to an orientation scotoma solution. In contrast to the low order variant this stripe pattern is stable for arbitrary large inter-map coupling. In case of OP rhombic solutions inter-map coupling transforms this solution by reducing the amplitudes $A_2 = A_{2^-}$ while increasing the amplitudes $A_3 = A_{3^-}$. Without OD bias this solution is then transformed into the orientation scotoma stripe pattern, similar to the low order variant of this energy. In contrast to the low order energy, for non-zero bias the amplitudes A_2 and A_3 stay small but non-zero.

The parameter dependence of OP solutions when interacting with OD hexagons is shown in Fig. S1**C,D**. For a small OD bias ($\gamma = \gamma^*$) OP rhombic solutions decay into OP stripe-like patterns. These stripe-like patterns stay stable also for large-inter map coupling. In case of a larger OD bias ($\gamma = 3\gamma^*$), both the OP stripe and the OP rhombic solutions decay into the uniform PWC solution. Thus for small bias there is a bistability between stripe-like and uniform PWC solutions while for larger OD bias the uniform PWC solution is the only stable solution. The potential of OP stripe and OP rhombic solutions is shown in Fig. S1**E,F**. In the uncoupled case as well as for small inter-map coupling strength OP stripe solutions are for all bias values the energetic ground state. For large inter-map coupling and a small bias ($\gamma \approx \gamma^*$) rhombic solutions are unstable and the stripe-like solutions are energetically preferred compared to PWC solutions. For larger bias, however, PWC solutions are the only stable solutions for large inter-map coupling.

Phase diagram

The stability properties of all stationary solutions are summarized in the phase diagram Fig. S2. Compared to the gradient-type interaction energy we cannot scale out the dependence on r_o. The phase diagram is thus plotted for $r_o = 0.2$. We rescale the inter-map coupling strength as $\tau\mathcal{B}^4$ where $\mathcal{B}$ is the stationary amplitude of the OD hexagons. In the regime of stable OD stripes there is a transition from OP stripes towards the orientation scotoma stripe solution. In the regime of stable OD hexagons there is a transition from OP stripes towards PWC solutions (red line). The stability border of PWC solutions is strongly OD bias dependent and has a peak at $\gamma \approx 2\gamma^*$. For small OD bias γ the uniform solution Eq. (27) is stable. With increasing bias there is a smooth transition of this solution until at $\gamma = \gamma_c$ the $d = \pi$ uniform solution becomes stable. In Fig. S2**C** the stability border γ_c between the two types of uniform solutions is plotted as a function of r_o. We observe that there is only a weak dependence on the

control parameter and $\gamma_c \approx 2\gamma^*$.

Interaction induced pinwheel crystals

Figure S3 illustrates the uniform solutions Eq. (27) for different values of the OD bias γ. For small bias, the OP pattern has six pinwheels per unit cell. Two of them are located at OD maxima while one is located at an OD minimum. The remaining three pinwheels are located near the OD border. With increasing bias, these three pinwheels are pushed further away from the OD border, being attracted to the OD maxima. With further increasing bias three shifted pinwheels merge with the one at the OD maximum building a single charge 1 pinwheel centered on a contralateral peak. The remaining two pinwheels are located at an ispi and contra peak, respectively. Note, compared to the Braitenberg PWC of the $\Delta = 0$ uniform solution the charge 1 pinwheel here is located at the contralateral OD peak. Finally, the charge 1 pinwheels split up again into four pinwheels. With increasing bias the solution more and more resembles the Ipsi-center PWC ($\Delta = \pi$ solution) which is stable also in the lower order version of the coupling energy. Finally, at $\gamma/\gamma^* \approx 2$ the Ipsi-center PWC becomes stable and fixed for $\gamma > 2\gamma^*$. The distribution of preferred orientations for different values of the bias γ is shown in Fig. S3**E,F**, reflecting the symmetry of each pattern. The distribution of intersection angles is shown in Fig. S3**G**. Remarkably, all solutions show a tendency towards perpendicular intersection angles. This tendency is more pronounced with increasing OD bias. At about $\gamma/\gamma^* \approx 1.9$ parallel intersection angles are completely absent and at $\gamma/\gamma^* \approx 2$ there are exclusively perpendicular intersection angles.

Gradient-type energy $U = \epsilon \, |\nabla o \cdot \nabla z|^4$

Finally, we examine the higher order version of the gradient-type inter-map coupling. The interaction terms are independent of the OD shift δ. In this case the coupling strength can be rescaled as $\beta\mathcal{B}^4$ and is therefore independent of the bias γ. The bias in this case only determines the stability of OD stripes, hexagons or the constant solution.

Stationary solutions and their stability

As for its lower order pendant a coupling to OD stripes is relatively easy to analyze. The energetic ground state corresponds to OP stripes with the direction perpendicular to the OD stripes for which $U = 0$. In addition, there are rhombic solutions with the stationary amplitudes $\mathcal{A}_1 = \mathcal{A}_{1^-} = \mathcal{A}_2 = \mathcal{A}_{2^-} =$

$\sqrt{r_z/(5+80\epsilon\mathcal{B}^4)}$. In case the OD map is a constant, $o(\mathbf{x}) = \delta$, the gradient-type inter-map coupling leaves the OP unaffected. As for its lower order pendant the stationary states are therefore OP stripes running in an arbitrary direction and the uncoupled rhombic solutions.

In case of OD hexagons we identified three types of non-uniform solutions. Besides stripe-like solutions of $z(\mathbf{x})$ with one dominant mode we find rPWCs $\mathcal{A}_j = \mathcal{A}_{j^-} = (\mathcal{A}, a, \mathcal{A})$ with $a \ll \mathcal{A}$ and distorted rPWCs $\mathcal{A}_j = (\mathcal{A}_1, \mathcal{A}_2, \mathcal{A}_3)\,, \mathcal{A}_{j^-} = (\mathcal{A}_3, \mathcal{A}_2, \mathcal{A}_1)$ with $\mathcal{A}_1 \neq \mathcal{A}_2 \neq \mathcal{A}_3$. Note, that distorted rPWCs are not stable in case of the product-type coupling energy or the analyzed low-order coupling energies. For these non-uniform solutions the stationary phases are inter-map coupling strength dependent.

In case of OD hexagons there are further uniform solutions $A_j = A_{j^-} = \mathcal{A}, B_j = \mathcal{B}$ and $\psi_1 = \psi_3 = 0, \psi_2 = \pi$. The imaginary part of the amplitude equations, see Text S2, leads to equations for the phases ϕ_j. The ansatz Eq. (25) leads to the stationarity condition

$$(13\cos\Delta - 5)\sin\Delta = 0\,. \tag{29}$$

The solutions are $\Delta = 0, \Delta = \pi$, and $\Delta = \pm\arccos\left(\frac{5}{13}\right) \approx \pm 1.176$ where the stationary amplitude are given by

$$\mathcal{A} = \sqrt{r_z/\left(9 + \epsilon\mathcal{B}^4\left(61.875 - 7.5\cos\Delta + 4.875\cos(2\Delta)\right)\right)}\,. \tag{30}$$

We calculated the stability properties of all stationary solutions by linear stability analysis considering perturbations of the amplitudes $\mathcal{A}_j \rightarrow \mathcal{A} + a_j$, $\mathcal{A}_{j^-} \rightarrow \mathcal{A} + a_{j^-}$ and of the phases $\phi_j \rightarrow \phi_j + \varphi_j$, $\phi_{j^-} \rightarrow \phi_{j^-} + \varphi_{j^-}$. This leads to a perturbation matrix M. In general amplitude and phase perturbations do not decouple. We therefore calculate the eigenvalues of the perturbation M matrix numerically. It turns out that for this type of coupling energy only the uniform solutions with $\Delta = \pm\arccos\left(\frac{5}{13}\right)$ are stable while the $\Delta = 0$ and $\Delta = \pi$ solutions are unstable in general.

Bifurcation diagram

For increasing inter-map coupling strength the amplitudes of the OP stripe and OP rhombic solutions are shown in Fig. S4**A**. In case of inter-map coupling strength dependent stationary phases, stationary solutions are calculated numerically using a Newton method and initial conditions close to these solutions. We followed the unstable solutions (dashed lines in Fig. S4) until this method did not converge anymore. Not shown are solutions which are unstable in general. In case of stable OD hexagons there is a transition

from rPWC (blue) towards distorted rPWC (green). The distorted rPWCs then decay into the hPWC (red). In case of OP stripes (black dashed lines) inter-map coupling leads to a slight suppression of the dominant mode and a growth of the remaining modes. This growth saturates at small amplitudes and thus the solution stays stripe-like. This stripe-like solution remains stable for arbitrary large inter-map coupling. Therefore there is a bistability between hPWC solutions and stripe-like solutions for large inter-map coupling.

The stability borders for the rPWC and distorted rPWC solutions were obtained by calculating their bifurcation diagram numerically from the amplitude equations, see Text S2. With increasing map coupling we observe a transition from a rPWC towards a distorted rPWC at $\epsilon\mathcal{B}^4 \approx 0.033$ (blue dashed line in Fig. S5**A**), see also Fig. S8**A**. The distorted rPWC loses its stability at $\epsilon\mathcal{B}^4 \approx 0.065$ (blue solid line in Fig. S5**A**) and from thereon all amplitudes are equal corresponding to the hPWC. There is a bistability between hPWC, rPWC, and stripe-like solutions. To calculate the inter-map coupling needed for the hexagonal solution to become the energetic ground state we calculated the potential Eq. (16) for the three solutions. In case of the uniform solution Eq. (25) the potential is given by

$$\begin{aligned} V &= -6\mathcal{A}^2 r_z - 3\mathcal{B}^2 r_o + 27\mathcal{A}^4 + \frac{45}{2}\mathcal{B}^4 \\ &\quad + \frac{1}{16}\mathcal{A}^4\mathcal{B}^4\epsilon\left(3210 - 456\cos\Delta + 90\cos(2\Delta)\right) . \end{aligned} \tag{31}$$

Above $\epsilon\mathcal{B}^4 \approx 0.12$ the hPWC is energetically preferred compared to stripe-like solutions (red dashed line in in Fig. S5) and thus corresponds to the energetic ground state for large inter-map coupling, see Fig. (S4)**B**.

Phase diagram

We calculated the phase diagram of the coupled system in the limit $r_z \ll r_o$, shown in Fig. S5. The phase diagram contains the stability borders of the uncoupled OD solutions $\gamma^*, \gamma_2^*, \gamma_3^*, \gamma_4^*$. They correspond to vertical lines, as they are independent of the inter-map coupling in the limit $r_z \ll r_o$. At $\gamma = \gamma^*$ hexagons become stable. Stripe solutions become unstable at $\gamma = \gamma_2^*$. At $\gamma = \gamma_3^*$ the homogeneous solution becomes stable while at $\gamma = \gamma_4^*$ hexagons loose their stability. In units γ/γ^* the borders $\gamma_2^*, \gamma_3^*, \gamma_4^*$ vary slightly with r_o , see Figure 9, and are drawn here for $r_o = 0.2$. We rescale the inter-map coupling strength as $\epsilon\mathcal{B}^4$ where $\mathcal{B}$ is the stationary amplitude of the OD hexagons. The stability borders of OP solutions are then

horizontal lines. For $\gamma < \gamma^*$ or for $\gamma > \gamma_4^*$ pinwheel free orientation stripes are dynamically selected. For $\gamma^* < \gamma < \gamma_4^*$ and above a critical effective coupling strength $\epsilon\mathcal{B}^4 \approx 0.042$ hPWC solutions are stable and become the energetic ground state of Eq. (16) above $\epsilon\mathcal{B}^4 \approx 0.117$. Below $\epsilon\mathcal{B}^4 \approx 0.065$, rPWC solutions are stable leading to a bistability region between rPWC and hPWC solutions. We find in this region that rhombic solutions transform into distorted rhombic solutions above an effective coupling strength of $\epsilon\mathcal{B}^4 \approx 0.033$.

Interaction induced pinwheel crystals

First, we studied the spatial layout of the rhombic solutions which is illustrated in Fig. S6. The rPWC solutions are symmetric under rotation by 180 degree. The rhombic solution has 4 pinwheels per unit cell and its pinwheel density is thus $\rho = 4\,\cos(\pi/6) \approx 3.5$. One may expect that the energy term Eq. (2) favors pinwheels to co-localize with OD extrema. In case of the rhombic layout there is only one pinwheel at an OD extremum while the other three pinwheels are located at OD saddle-points which are also energetically favorable positions with respect to U. The orientation selectivity $|z(\mathbf{x})|$ for the rPWC is shown in Fig. S6**B**. The pattern of selectivity is arranged in small patches of highly selective regions.
The hexagonal layout of the two stable uniform solutions is shown in Fig. S7. The $\Delta = \pm\,\text{arccos}(5/13)$ solutions have six pinwheels per unit cell. Their pinwheel density is therefore $\rho = 6\cos\pi/6 \approx 5.2$. Three pinwheels of the same topological charge are located at the extrema of the OD map. Two of these are located at the OD maximum while one is located at the OD minimum. The remaining three pinwheels are not at an OD extremum but near the OD border. The distance to the OD border depends on the OD bias, see Fig. S7**D**. For a small bias ($\gamma \approx \gamma^*$) these three pinwheels are close to the OD borders and with increasing bias the OD border moves away from the pinwheels. The pinwheel in the center of the OP hexagon is at the contralateral OD peak. Because these pinwheels organize most of the map while the others essentially only match one OP hexagon to its neighbors we refer to this pinwheel crystal as the *Contra-center pinwheel crystal.* Note, that some pinwheels are located at the vertices of the hexagonal pattern. Pinwheels located between these vertices (on the edge) are not in the middle of this edge. Solutions with $\Delta = \pm\,\text{arccos}(5/13)$ are therefore not symmetric under a rotation by 60 degree but symmetric under a rotation by 120 degree. Therefore the solution $\Delta = +\,\text{arccos}(5/13)$ cannot be transformed into the solution $\Delta = -\,\text{arccos}(5/13)$ by a rotation of the OD and OP pattern by 180 degrees. This symmetry is also reflected by the distribution of preferred orientations, see Fig. S7**F**. Six

orientations are slightly overrepresented. Compared to the Ipsi-center PWC, which have a 60° symmetry, this distribution illustrates the 120° symmetry of the pattern. The distribution of intersection angles is continuous, see Fig. S7**C**. Although there is a fixed uniform solution with varying OD bias the distribution of intersection angles changes. The reason for this is the bias dependent change in the OD borders, see Fig. S7**D**. For all bias values there is a tendency towards perpendicular intersection angles, although for low OD bias there is an additional small peak at parallel intersection angles. The orientation selectivity $|z(\mathbf{x})|$ for the hPWC is shown in Fig. S7**E**. The pattern shows hexagonal bands of high selectivity.
Finally, we study changes in pinwheel positions during the transition from a rPWC towards a hPWC i.e. with increasing inter-map coupling strength. In case of the higher order gradient-type coupling energy there is a transition towards a contra-center PWC, see Fig. S8**A**. In the regime where the distorted rPWC is stable, three of the four pinwheels of the rPWC are moving either from an OD saddle-point to a position near an OD border (pinwheel 1 and 3) or from an OD saddle-point to an OD extremum (pinwheel 4). One pinwheel (pinwheel 2) is fixed in space. At the transition to the hPWC two additional pinwheels are created, one near an OD border (pinwheel 5) and one at an OD extremum (pinwheel 6). We compare the inter-map coupling strength dependent pinwheel positions of the gradient-type coupling energy with those of the product type coupling energy, see Fig. (S8)**B**. In this case three (pinwheel 2,3,4) of the four rPWC pinwheels have a inter-map coupling strength independent position. The remaining pinwheel (pinwheel 1) with increasing inter-map coupling strength splits up into three pinwheels. While one of these three pinwheels (pinwheel 1) is fixed in space the remaining two pinwheels (pinwheel 5,6) move towards the extrema of OD. Thus for large inter-map coupling, where hPWC solutions are stable, all six pinwheels are located at OD extrema.

Summary

We derived amplitude equations and analyzed ground states of the higher order inter-map coupling energies. We calculated local and global optima and derived corresponding phase diagrams. A main difference between phase diagrams for low order and high order coupling energies consists in the collapse of orientation selectivity above a critical coupling strength that occurs only in the low order models. In contrast, for the high order versions, orientation selectivity is preserved for arbitrarily strong inter-map coupling. In order to neglect the backreaction on the dynamics of the modes B we assumed $A \ll B \ll 1$.

Our results, however, show that for the stability of pinwheel crystals a finite amplitude B is necessary. A decrease in B cannot be compensated by another parameter (as it would be r_z in case of the low order inter-map coupling energies). For a finite B even higher order corrections to the amplitude equations than those presented here can thus become significant. Such terms are we neglected in the present treatment. In part (II) of this study we numerically confirm our main results for the higher order inter-map coupling energies.

From a practical point of view, the analyzed phase diagrams and pattern properties indicate that the higher order gradient-type coupling energy is the simplest and most convenient choice for constructing models that reflect the correlations of map layouts in the visual cortex. For this coupling, intersection angle statistics are reproduced well, pinwheels can be stabilized, and pattern collapse cannot occur.

Figure Legends

Figure S1. Stationary amplitudes with coupling energy $\mathbf{U} = \tau|\mathbf{z}|^4\mathbf{o}^4$. Solid (dashed) lines: stable (unstable) solutions. **A,B** OD stripes, $\gamma = 0$ (blue), $\gamma = \gamma^*$ (green), $\gamma = 1.4\gamma^*$ (red). **C,D** OD hexagons, $\gamma = \gamma^*$ (blue), $\gamma = 3\gamma^*$ (red). **A,C** Transition from OP stripe solutions, **B,D** Transition from OP rhombic solutions. **E** Potential, Eq. (16), of OP stripes and OP rhombs interacting with OD stripes. **F** Potential, Eq. (16), of OP stripes, OP rhombs, and hPWC interacting with OD hexagons. Arrows indicate corresponding lines in the phase diagram, Fig. (S2).

Figure S2. A Phase diagram with coupling energy $\mathbf{U} = \tau\mathbf{o}^4|\mathbf{z}|^4$, $r_o = 0.2, r_z \ll r_o$. Vertical black lines: stability range of OD stripes, hexagons, and constant solutions. Magenta (orange) line: Stability border of orientation scotoma stripes. Green solid line: Stability border of rhombic solutions. Red solid line: Stability border of PWC solutions, red dashed line: γ_c, **B** Course of Eq. (27), dashed line: $\Delta = \pi$. **C** Stability border between Eq. (27) solution and the $\Delta = \pi$ solution as a function of r_o (vertical red line in **A**).

Figure S3. Bias dependent pinwheel crystals, Eq. (27) **A** $\gamma = \gamma^*$, **B** $\gamma = 1.3\,\gamma^*$, **C** $\gamma = 1.6\,\gamma^*$, **D** $\gamma = 2\,\gamma^*$. OP map, superimposed are the OD borders (gray), 90% ipsilateral eye dominance (black), and 90% contralateral eye dominance (white), $r_o = 0.2$. Dashed lines mark the unit cell of the regular pattern. **E,F** Distribution of orientation preference. **G** Intersection angles between iso-orientation lines and OD borders.

Figure S4. Stationary amplitudes with coupling energy $\mathbf{U} = \epsilon\,|\nabla\mathbf{z}\cdot\nabla\mathbf{o}|^4$, **A** Solid (dashed) lines: Stable (unstable) solutions. Blue: rPWC, green: distorted rPWC, red: hPWC. Black lines: stripe-like solutions. **B** Potential, Eq. (16), of OP stripes (black), OP rhombs (blue), and hPWC solutions (red). Arrows indicate corresponding lines in the phase diagram, Fig. (S5).

Figure S5. Phase diagram with coupling energy $\mathbf{U} = \epsilon\,|\nabla\mathbf{z}\cdot\nabla\mathbf{o}|^4$, for $r_z \ll r_o$. Vertical lines: stability range of OD hexagons, green line: transition from rPWC to distorted rPWC, red line: stability border of hPWC, blue line: stability border of distorted rPWC. Above orange line: hPWC corresponds to ground state of energy.

Figure S6. Rhombic pinwheel crystals. A OP map with superimposed OD borders (gray), 90% ipsilateral eye dominance (black), and 90% contralateral eye dominance (white), $\gamma = 3\gamma^*, r_o = 0.2$. **B** Selectivity $|z(\mathbf{x})|$, white: high selectivity, black: low selectivity.

Figure S7. Contra-center pinwheel crystals. **A,B** OP map, superimposed are the OD borders (gray), 90% ipsilateral eye dominance (black), and 90% contralateral eye dominance (white), $r_o = 0.2, \gamma = 3\gamma^*$. **A** $\Delta = \arccos(5/13)$, **B** $\Delta = -\arccos(5/13)$. **C** Distribution of orientation preference. **D** OP map with superimposed OD map for three different values $(\gamma = \gamma^*, \gamma = (\gamma_4^* - \gamma^*)/2 + \gamma^*, \gamma = \gamma_4^*)$ of the OD bias. **E** Selectivity $|z(\mathbf{x})|$, white: high selectivity, black: low selectivity. **F** Distribution of intersection angles.

Figure S8. Inter-map coupling strength dependent pinwheel positions. OD map, superimposed pinwheel positions (points) for different inter-map coupling strengths, $\gamma/\gamma^* = 3$. Numbers label pinwheels within the unit cell (dashed lines). Blue (green, red) points: pinwheel positions for rPWC (distorted rPWC, hPWC) solutions. **A** $\mathbf{U} = \epsilon\,|\nabla\mathbf{z}\cdot\nabla\mathbf{o}|^\mathbf{4}$, using stationary amplitudes from Fig. (S4)(a). Positions of distorted rPWCs move continuously (pinwheel 1,3,4). **B** $\mathbf{U} = \tau\,|\mathbf{z}|^\mathbf{4}\mathbf{o}^\mathbf{4}$, using stationary amplitudes from Fig. (S1). **D** Positions of rPWCs move continuously (pinwheel 5,6).
