# Supplementary material for: Coordinated Optimization of Visual Cortical Maps (I) Symmetry-based Analysis
Source: PLoS Comput Biol. 2012 Nov 8;8(11):e1002466. doi: 10.1371/journal.pcbi.1002466 (PMC3493482; doi:10.1371/journal.pcbi.1002466)
Supplement: Text S2 — Amplitude equations for the OP dynamics in case of the high order inter-map coupling energies and . (PDF) [file pcbi.1002466.s010.pdf]

Text S2: Supporting Information for

Coordinated optimization of visual cortical maps (I) Symmetry-based analysis

1 Amplitude equations for higher order coupling energies

Here, we list the amplitude equations for the OP dynamics in case of the high order inter-map coupling energies $U = \epsilon|\nabla z \cdot \nabla o|^4$ and $U = \tau|z|^4 o^4$.

$$
\begin{aligned}
\partial_t A_i \;=\;& r_z A_i - \sum_j |A_j|^2 A_i \Bigg(g_{ij} + \delta^4 g'_{ij} + \delta^2 \sum_u g_{iju}|B_u|^2 \\
&+ \delta g''_{ij} \left(B_1 B_2 B_3 + \overline{B}_1 \overline{B}_2 \overline{B}_3\right) + \sum_{u,v} g_{ijuv}|B_u|^2|B_v|^2 \Bigg) \\
&- \sum_j |A_{j^-}|^2 A_i \Bigg(g_{ij^-} + \delta^4 g'_{ij^-} + \delta^2 \sum_u g_{ij^-u}|B_u|^2 \\
&+ \delta g''_{ij^-} \left(B_1 B_2 B_3 + \overline{B}_1 \overline{B}_2 \overline{B}_3\right) + \sum_{u,v}^{3} g_{ij^-uv}|B_u|^2|B_v|^2 \Bigg) \\
&- \sum_j A_j A_{j^-} \overline{A}_{i^-} \Bigg(f_{ij} + \delta^4 f'_{ij} + \delta^2 \sum_u f_{iju}|B_u|^2 \\
&+ \delta f''_{ij} \left(B_1 B_2 B_3 + \overline{B}_1 \overline{B}_2 \overline{B}_3\right) + \sum_{u,v} f_{ijuv}|B_u|^2|B_v|^2 \Bigg) \\
&- \sum_{j \neq l} A_i A_j \overline{A}_l \left(\sum_u h^{(1)}_{ijlu} \overline{B}_j B_l |B_u|^2 + \delta^2 h^{(1)}_{ijl} \overline{B}_j B_l \right) \\
&- \sum_{j,l} A_i A_{j^-} \overline{A}_l \left(\sum_u h^{(1)}_{ij^-lu} B_j B_l |B_u|^2 + \delta^2 h^{(1)}_{ij^-l} B_j B_l \right) \\
&- \sum_{j,l} A_i A_j \overline{A}_{l^-} \left(\sum_u h^{(1)}_{ijl^-u} \overline{B}_j \overline{B}_l |B_u|^2 + \delta^2 h^{(1)}_{ijl^-} \overline{B}_j \overline{B}_l \right) \\
&- \sum_{j \neq l} A_i A_{j^-} \overline{A}_{l^-} \left(\sum_u h^{(1)}_{ij^-l^-u} B_j \overline{B}_l |B_u|^2 + \delta^2 h^{(1)}_{ij^-l^-} B_j \overline{B}_l \right) \\
&- \sum_{l,j \neq k} A_j A_l \overline{A}_k h^{(2)}_{ijlk} \overline{B}_j \overline{B}_l B_k B_i - \sum_{l,j,k} A_{j^-} A_l \overline{A}_k h^{(2)}_{ij^-lk} B_j \overline{B}_l B_k B_i \\
&- \sum_{l,j \neq k} A_j A_l \overline{A}_{k^-} h^{(2)}_{ijlk^-} \overline{B}_j \overline{B}_l \overline{B}_k B_i - \sum_{l,j \neq k} A_{j^-} A_{l^-} \overline{A}_k h^{(2)}_{ij^-l^-k} B_j B_l B_k B_i
\end{aligned}
$$

$$
\begin{aligned}
&-\sum_{l,j\neq k} A_{j^-}A_l\overline{A}_{k^-}h^{(2)}_{ij^-lk^-}B_j\overline{B}_l\overline{B}_kB_i-\sum_{l,j\neq k} A_{j^-}A_{l^-}\overline{A}_{k^-}h^{(2)}_{ij^-l^-k^-}B_jB_l\overline{B}_kB_i\\
&-\sum_{j,l}|A_j|^2A_l\left(\sum_u h^{(3)}_{ijlu}|B_u|^2\overline{B}_lB_i+\delta^2h^{(3)}_{ijl}\overline{B}_lB_i\right)\\
&-\sum_{j,l}|A_{j^-}|^2A_l\left(\sum_u h^{(3)}_{ij^-lu}|B_u|^2\overline{B}_lB_i+\delta^2h^{(3)}_{ij^-l}\overline{B}_lB_i\right)\\
&-\sum_{j,l}|A_j|^2A_{l^-}\left(\sum_u h^{(3)}_{ijl^-u}|B_u|^2B_lB_i+\delta^2h^{(3)}_{ijl^-}B_lB_i\right)\\
&-\sum_{j,l}|A_{j^-}|^2A_{l^-}\left(\sum_u h^{(3)}_{ij^-l^-u}|B_u|^2B_lB_i+\delta^2h^{(3)}_{ij^-l^-}B_lB_i\right)\\
&-\sum_{j,u}\delta h^{(4)}_{iju}A_i\overline{A}_jA_uB_{u+1}B_{u+2}B_j-\sum_{j,u}\delta h^{(4)}_{iuj}A_iA_j\overline{A}_u\overline{B}_{u+1}\overline{B}_{u+2}\overline{B}_j\\
&-\sum_{j,u}\delta h^{(4)}_{ij^-u}A_i\overline{A}_{j^-}A_uB_{u+1}B_{u+2}\overline{B}_j-\sum_{j,u}\delta h^{(4)}_{ij^-u}A_iA_{j^-}\overline{A}_u\overline{B}_{u+1}\overline{B}_{u+2}\overline{B}_j\\
&-\sum_{j,u}\delta h^{(4)}_{iju^-}A_i\overline{A}_jA_{u^-}\overline{B}_{u+1}\overline{B}_{u+2}B_j-\sum_{j,u}\delta h^{(4)}_{iju^-}A_iA_j\overline{A}_{u^-}B_{u+1}B_{u+2}B_j\\
&-\sum_{j,u}\delta h^{(4)}_{ij^-u^-}A_i\overline{A}_{j^-}A_{u^-}\overline{B}_{u+1}\overline{B}_{u+2}\overline{B}_j-\sum_{j,u}\delta h^{(4)}_{ij^-u^-}A_iA_{j^-}\overline{A}_{u^-}B_{u+1}B_{u+2}\overline{B}_j\\
&-\sum_u h^{(5)}_{iu}A_iA_u\overline{A}_{(u+1)^-}B_{u+2}B_1B_2B_3-\sum_u h^{(5)}_{iu^-}A_iA_{u^-}\overline{A}_{(u+1)}\overline{B}_{u+2}B_1B_2B_3\\
&-\sum_u \tilde{h}^{(5)}_{iu}A_iA_u\overline{A}_{(u+1)^-}B_{u+2}\overline{B}_1\overline{B}_2\overline{B}_3-\sum_u \tilde{h}^{(5)}_{iu^-}A_iA_{u^-}\overline{A}_{(u+1)}\overline{B}_{u+2}\overline{B}_1\overline{B}_2\overline{B}_3\\
&-\sum_u \delta^3h^{(5)}A_iA_u\overline{A}_{(u+1)^-}B_{u+2}-\sum_u \delta^3h^{(5)}A_iA_{u^-}\overline{A}_{(u+1)}\overline{B}_{u+2}\\
&-\sum_{j,u}\delta h^{(6)}_{iju}\overline{A}_jA_uA_{u+1}B_{u+2}B_jB_i-\sum_{j,u}\delta h^{(6)}_{ij^-u}\overline{A}_{j^-}A_uA_{u+1}B_{u+2}\overline{B}_jB_i\\
&-\sum_{j,u}\delta h^{(6)}_{iju^-}\overline{A}_jA_{u^-}A_{(u+1)^-}\overline{B}_{u+2}B_jB_i-\sum_{j,u}\delta h^{(6)}_{ij^-u^-}\overline{A}_{j^-}A_{u^-}A_{(u+1)^-}\overline{B}_{u+2}\overline{B}_jB_i\\
&-\sum\delta h^{(7)}_{iju}|A_j|^2A_uB_{u+1}B_{u+2}B_i-\sum\delta h^{(7)}_{ij^-u}|A_{j^-}|^2A_uB_{u+1}B_{u+2}B_i\\
&-\sum\delta h^{(7)}_{iju^-}|A_j|^2A_{u^-}\overline{B}_{u+1}\overline{B}_{u+2}B_i-\sum\delta h^{(7)}_{ij^-u^-}|A_{j^-}|^2A_{u^-}\overline{B}_{u+1}\overline{B}_{u+2}B_i\\
&-\sum\delta h^{(8)}_{ij}A_j^2\overline{A}_{i+2}\overline{B}_j^2\overline{B}_{i+1}-\sum\delta h^{(8)}_{ij^-}A_{j^-}^2\overline{A}_{i+2}B_j^2\overline{B}_{i+1}\\
&-\sum\delta^3h^{(9)}_{ij}|A_j|^2A_{(i+2)^-}\overline{B}_{i+1}-\sum\delta^3h^{(9)}_{ij^-}|A_{j^-}|^2A_{(i+2)^-}\overline{B}_{i+1}
\end{aligned}
$$

where the indices are considered to be cyclic i.e. $j+3=j$. All sums are considered to run from 1 to 3. In the article, these amplitude equations are specified in case of OD stripes, Eq. (109), OD hexagons, Eq. (110), or a constant OD solution, Eq (111).

2 Coupling coefficients

In the following, we list the non-zero elements of the coupling coefficients.

$g_{ii} = 1$	$g_{ij} = 2$	$g'_{ii} = \tau$	$g'_{ij} = 2\tau$
$g_{iji} = g_{ijj} = g_{iju} = 24\tau$	$g_{iii} = g_{iij} = 12\tau$		
$g''_{ij} = 48\tau$	$g''_{ii} = 24\tau$		
$g_{ijii} = 6\epsilon + 12\tau$	$g_{ijij} = 33\epsilon + 48\tau$	$g_{ijjj} = 6\epsilon + 12\tau$	$g_{ijiu} = 3\epsilon + 48\tau$
$g_{ijju} = -3\epsilon + 48\tau$	$g_{ijuu} = 1.5\epsilon + 12\tau$		
$f_{ij} = 2$	$f'_{ij} = 2\tau$		
$f_{iju} = f_{ijj} = f_{iji} = 24\tau$	$f''_{ij} = 48\tau$		
$f_{ijii} = 12\tau$	$f_{ijij} = 48\tau + 33\epsilon$	$f_{ijjj} = 12\tau + 6\epsilon$	$f_{ijiu} = 48\tau + 3\epsilon$
$f_{ijju} = 48\tau - 3\epsilon$	$f_{ijuu} = 12\tau + 1.5\epsilon$		
$h^{(1)}_{ijli} = 15\epsilon + 48\tau$	$h^{(1)}_{ijlj} = 0.75\epsilon + 24\tau$	$h^{(1)}_{ijll} = 0.75\epsilon + 24\tau$	
$h^{(1)}_{ijii} = 21\epsilon + 24\tau$	$h^{(1)}_{ijij} = 9.75\epsilon + 24\tau$	$h^{(1)}_{ijil} = 1.5\epsilon + 48\tau$	
$h^{(1)}_{iiji} = 10.5\epsilon + 12\tau$	$h^{(1)}_{iijj} = 4.875\epsilon + 12\tau$	$h^{(1)}_{iiju} = 0.75\epsilon + 24\tau$	
$h^{(1)}_{ij-li} = 15\epsilon + 48\tau$	$h^{(1)}_{ij-lj} = h^{(1)}_{ij-ll} = 0.75\epsilon + 24\tau$		
$h^{(1)}_{ijl-i} = 15\epsilon + 48\tau$	$h^{(1)}_{ijl-j} = h^{(1)}_{ijl-l} = 0.75\epsilon + 24\tau$		
$h^{(1)}_{ij-l-i} = 15\epsilon + 48\tau$	$h^{(1)}_{ij-l-j} = h^{(1)}_{ij-l-l} = 0.75\epsilon + 24\tau$		
$h^{(1)}_{ijl} = 24\tau$	$h^{(1)}_{iij} = 12\tau$	$h^{(1)}_{ij-l} = h^{(1)}_{ii-j} = 24\tau$	$h^{(1)}_{ij-j} = 12\tau$
$h^{(1)}_{ijl-} = 24\tau$	$h^{(1)}_{iij-} = 12\tau$	$h^{(1)}_{iii-} = 6\tau$	$h^{(1)}_{iji-} = 24\tau$
$h^{(1)}_{ij-l-} = 24\tau$	$h^{(1)}_{ii-j-} = 24\tau$	$h^{(1)}_{ij-i-} = 24\tau$	
$h^{(2)}_{ijli} = 7.5\epsilon + 24\tau$	$h^{(2)}_{iiij} = 10.5\epsilon + 12\tau$	$h^{(2)}_{ij-li} = 7.5\epsilon + 24\tau$	$h^{(2)}_{ii-ij} = 21\epsilon + 24\tau$
$h^{(2)}_{ijli-} = 15\epsilon + 48\tau$	$h^{(2)}_{ijji-} = 8.25\epsilon + 12\tau$	$h^{(2)}_{ijjl-} = 3.75\epsilon + 12\tau$	
$h^{(2)}_{ij-l-i} = 7.5\epsilon + 24\tau$	$h^{(2)}_{ij-l-j} = 7.5\epsilon + 24\tau$	$h^{(2)}_{ij-l-l} = 7.5\epsilon + 24\tau$	
$h^{(2)}_{ij-ll-} = 7.5\epsilon + 24\tau$	$h^{(2)}_{ij-li-} = 15\epsilon + 48\tau$		
$h^{(2)}_{ij-l-i-} = 15\epsilon + 48\tau$	$h^{(2)}_{ij-j-i-} = 8.25\epsilon + 12\tau$	$h^{(2)}_{ij-j-l-} = 3.75\epsilon + 12\tau$	
$h^{(3)}_{ijli} = 0.75\epsilon + 24\tau$	$h^{(3)}_{ijlj} = 15\epsilon + 48\tau$	$h^{(3)}_{ijll} = 0.75\epsilon + 24\tau$	$h^{(3)}_{ijji} = 4.875\epsilon + 12\tau$
$h^{(3)}_{ijjj} = 10.5\epsilon + 12\tau$	$h^{(3)}_{ijjl} = 0.75\epsilon + 24\tau$	$h^{(3)}_{ijl} = 24\tau$	$h^{(3)}_{ijj} = 12\tau$
$h^{(3)}_{iiji} = 12\epsilon + 24\tau$	$h^{(3)}_{iijj} = 9.75\epsilon + 24\tau$	$h^{(3)}_{iijl} = 1.5\epsilon + 48\tau$	$h^{(3)}_{iij} = 24\tau$
$h^{(3)}_{ii-ji} = 21\epsilon + 24\tau$	$h^{(3)}_{ii-jj} = 9.75\epsilon + 24\tau$	$h^{(3)}_{ii-jl} = 1.5\epsilon + 48\tau$	
$h^{(3)}_{ij-ji} = 9.75\epsilon + 24\tau$	$h^{(3)}_{ij-jj} = 21\epsilon + 24\tau$	$h^{(3)}_{ij-jl} = 15\epsilon + 48\tau$	
$h^{(3)}_{ij-li} = 0.75\epsilon + 24\tau$	$h^{(3)}_{ij-lj} = 0.75\epsilon + 24\tau$	$h^{(3)}_{ij-ll} = 15\epsilon + 48\tau$	

$$h^{(3)}_{ii^{-}j} = 24\tau \qquad h^{(3)}_{ij^{-}j} = 24\tau \qquad h^{(3)}_{ij^{-}l} = 24\tau$$

$$h^{(3)}_{iii^{-}i} = 16\epsilon + 8\tau \qquad h^{(3)}_{iii^{-}j} = 12\epsilon + 24\tau \qquad h^{(3)}_{iji^{-}i} = 4\epsilon + 8\tau \qquad h^{(3)}_{iji^{-}j} = 16.5\epsilon + 24\tau$$

$$h^{(3)}_{iji^{-}l} = 1.5\epsilon + 24\tau \qquad h^{(3)}_{iij^{-}i} = 21\epsilon + 24\tau \qquad h^{(3)}_{iij^{-}j} = 9.75\epsilon + 24\tau \qquad h^{(3)}_{iij^{-}l} = 1.5\epsilon + 48\tau$$

$$h^{(3)}_{ijj^{-}i} = 9.75\epsilon + 24\tau \qquad h^{(3)}_{ijj^{-}j} = 21\epsilon + 24\tau \qquad h^{(3)}_{ijj^{-}l} = 1.5\epsilon + 48\tau \qquad h^{(3)}_{ijl^{-}i} = 0.75\epsilon + 24\tau$$

$$h^{(3)}_{ijl^{-}j} = 15\epsilon + 48\tau \qquad h^{(3)}_{ijl^{-}l} = 0.75\epsilon + 24\tau \qquad h^{(3)}_{iii^{-}} = 12\tau \qquad h^{(3)}_{iji^{-}} = 12\tau$$

$$h^{(3)}_{iij^{-}} = 24\tau \qquad h^{(3)}_{ijj^{-}} = 24\tau \qquad h^{(3)}_{ijl^{-}} = 24\tau$$

$$h^{(3)}_{ii^{-}i^{-}i} = 8\epsilon + 4\tau \qquad h^{(3)}_{ii^{-}i^{-}j} = 6\epsilon + 12\tau \qquad h^{(3)}_{ii^{-}j^{-}i} = 21\epsilon + 24\tau \qquad h^{(3)}_{ii^{-}j^{-}j} = 9.75\epsilon + 24\tau$$

$$h^{(3)}_{ii^{-}j^{-}l} = 1.5\epsilon + 48\tau \qquad h^{(3)}_{ij^{-}i^{-}i} = 4\epsilon + 8\tau \qquad h^{(3)}_{ij^{-}i^{-}j} = 16.5\epsilon + 24\tau \qquad h^{(3)}_{ij^{-}i^{-}l} = 1.5\epsilon + 24\tau$$

$$h^{(3)}_{ij^{-}j^{-}i} = 4.875\epsilon + 24\tau \qquad h^{(3)}_{ij^{-}j^{-}j} = 10.5\epsilon + 12\tau \qquad h^{(3)}_{ij^{-}j^{-}l} = 0.75\epsilon + 24\tau \qquad h^{(3)}_{ij^{-}l^{-}i} = 0.75\epsilon + 24\tau$$

$$h^{(3)}_{ij^{-}l^{-}j} = 15\epsilon + 48\tau \qquad h^{(3)}_{ij^{-}l^{-}l} = 0.75\epsilon + 24\tau \qquad h^{(3)}_{ii^{-}i^{-}} = 6\tau \qquad h^{(3)}_{ij^{-}j^{-}} = 12\tau$$

$$h^{(3)}_{ii^{-}j^{-}} = 24\tau \qquad h^{(3)}_{ij^{-}l^{-}} = 24\tau \qquad h^{(3)}_{ii^{-}i^{-}} = 12\tau$$

$$h^{(4)}_{iji} = 12\tau \qquad h^{(4)}_{ijl} = 24\tau \qquad h^{(4)}_{ii^{-}i} = h^{(4)}_{ij^{-}i} = 24\tau \qquad h^{(4)}_{ii^{-}j} = 48\tau$$

$$h^{(4)}_{ijj^{-}j} = h^{(4)}_{ij^{-}l} = 48\tau \qquad h^{(4)}_{iji^{-}} = h^{(4)}_{ijj^{-}} = h^{(4)}_{ijl^{-}} = 48\tau \qquad h^{(4)}_{ij^{-}i^{-}} = h^{(4)}_{ij^{-}l^{-}} = 24\tau$$

$$h^{(5)}_{ii} = 0.375\epsilon + 12\tau \qquad \tilde{h}^{(5)}_{ii} = 2h^{(5)}_{ii} \qquad h^{(5)}_{ii+1} = 7.5\epsilon + 48\tau \qquad \tilde{h}^{(5)}_{ii+1} = 2h^{(5)}_{ii+1}$$

$$h^{(5)}_{ii+2} = 0.75\epsilon + 24\tau \qquad \tilde{h}^{(5)}_{ii+2} = 2h^{(5)}_{ii+2} \qquad h^{(5)}_{ii^{-}} = 1.5\epsilon + 48\tau \qquad \tilde{h}^{(5)}_{ii^{-}} = 1/2h^{(5)}_{ii^{-}}$$

$$h^{(5)}_{i(i+1)^{-}} = 15\epsilon + 48\tau \qquad \tilde{h}^{(5)}_{i(i+1)^{-}} = 1/2h^{(5)}_{i(i+1)^{-}}$$

$$h^{(5)} = 8\tau$$

$$h^{(6)}_{ijj} = h^{(6)}_{iju} = 24\tau \qquad h^{(6)}_{iij} = 8\tau \qquad h^{(6)}_{ii^{-}i} = h^{(6)}_{ij^{-}i} = 48\tau \qquad h^{(6)}_{ii^{-}j} = h^{(6)}_{ij^{-}j} = 24\tau$$

$$h^{(6)}_{iii^{-}} = h^{(6)}_{iij^{-}} = 24\tau \qquad h^{(6)}_{iji^{-}} = h^{(6)}_{ijj^{-}} = h^{(6)}_{iju^{-}} = 48\tau$$

$$h^{(6)}_{ij^{-}j^{-}} = h^{(6)}_{ii^{-}i^{-}} = 48\tau \qquad h^{(6)}_{ij^{-}u^{-}} = h^{(6)}_{ii^{-}j^{-}} = 24\tau \qquad h^{(6)}_{ij^{-}i^{-}} = 48\tau$$

$$h^{(7)}_{ijj} = 12\tau \qquad h^{(7)}_{iij} = h^{(7)}_{ijl} = 24\tau \qquad h^{(7)}_{ii^{-}j} = h^{(7)}_{ij^{-}j} = h^{(7)}_{ij^{-}l} = 24\tau$$

$$h^{(7)}_{iii^{-}} = h^{(7)}_{iij^{-}} = h^{(7)}_{iji^{-}} = 48\tau \qquad h^{(7)}_{ijj^{-}} = h^{(7)}_{ijl^{-}} = 48\tau$$

$$h^{(7)}_{ii^{-}j^{-}} = h^{(7)}_{ij^{-}i^{-}} = 48\tau \qquad h^{(7)}_{ii^{-}i^{-}} = h^{(7)}_{ij^{-}j^{-}} = 24\tau \qquad h^{(7)}_{ij^{-}l^{-}} = 48\tau$$

$$h^{(8)}_{ii} = h^{(8)}_{ij} = 12\tau \qquad h^{(8)}_{ii^{-}} = h^{(8)}_{ij^{-}} = 12\tau$$

$$h^{(9)}_{ij} = h^{(9)}_{ii} = 8\tau$$

Note, that coupling coefficients involving the constant shift δ only occur in case of the product-type inter-map coupling energy.
