# Supplementary material for: Coordinated Optimization of Visual Cortical Maps (I) Symmetry-based Analysis
Source: PLoS Comput Biol. 2012 Nov 8;8(11):e1002466. doi: 10.1371/journal.pcbi.1002466 (PMC3493482; doi:10.1371/journal.pcbi.1002466)
Supplement: Text S3 — Stability matrices for the low order inter-map coupling energies. (PDF) [file pcbi.1002466.s011.pdf]

Text S3: Supporting Information for

Coordinated optimization of visual cortical maps (I) Symmetry-based analysis

Stability matrix

Here, we state the stability matrices M used in the linear stability analysis of the coupled amplitude equations. The stability matrix is defined by

$$\partial_t \begin{pmatrix} \mathrm{Re}A_i \\ \mathrm{Re}A_{i^-} \\ \mathrm{Im}A_i \\ \mathrm{Im}A_{i^-} \end{pmatrix} = M \begin{pmatrix} \mathrm{Re}A_i \\ \mathrm{Re}A_{i^-} \\ \mathrm{Im}A_i \\ \mathrm{Im}A_{i^-} \end{pmatrix} = \begin{pmatrix} M1 & M2 \\ M3 & M4 \end{pmatrix} \begin{pmatrix} \mathrm{Re}A_i \\ \mathrm{Re}A_{i^-} \\ \mathrm{Im}A_i \\ \mathrm{Im}A_{i^-} \end{pmatrix}$$

for the uniform solutions Eq. (60). We separate the uncoupled contributions from the inter-map coupling contributions i.e. $M1 = M1^{(u)} + M1^{(\alpha)}$. The uncoupled contributions are given by

$$M1^{(u)} = r_z\mathbf{I}+\mathcal{A}^2 \begin{pmatrix} -13 & -3 & 3 & -2c & -3c-\sqrt{3}s & 3c-\sqrt{3}s \\ -3 & -23/2 & 0 & -3c-\sqrt{3}s & -2c & 0 \\ 3 & 0 & -23/2 & 3c-\sqrt{3}s & 0 & -2c \\ -2c & -3c-\sqrt{3}s & 3c-\sqrt{3}s & -12-\cos 2\Delta & -2-2u & 2+2v \\ -3d+\sqrt{3}s & -2d & 0 & -2-2u & -12-\cos(2\Delta+10\pi/3) & -4d^2 \\ 3c-\sqrt{3}s & 0 & -2c & -2-2v & -4s^2 & -12+u \end{pmatrix}$$

$$M2^{(u)} = \mathcal{A}^2 \begin{pmatrix} 0 & \sqrt{3} & \sqrt{3} & 2s & 2\cos(\Delta-\pi/6) & 2\cos(\Delta+\pi/6) \\ \sqrt{3} & \sqrt{3}/2 & 0 & 2\cos(\Delta-\pi/6) & 2s & 4s \\ \sqrt{3} & 0 & -\sqrt{3}/2 & 2\cos(\Delta+\pi/6) & 4s & 2s \\ -2s & \sqrt{3}d-3s & \sqrt{3}d+3s & -\sin 2\Delta & 2x & 2y \\ \sqrt{3}d-2s & -2d & 0 & 2x & y & 2\sin 2\Delta \\ \sqrt{3}d+3s & 0 & -2s & 2y & 2\sin 2\Delta & -x \end{pmatrix}$$

$$M3^{(u)} = \mathcal{A}^2 \begin{pmatrix} 0 & \sqrt{3} & \sqrt{3} & 2s & 2\cos(\Delta-\pi/6) & 2\cos(\Delta+\pi/6) \\ \sqrt{3} & \sqrt{3}/2 & 0 & 2\cos(\Delta-\pi/6) & 2s & 4s \\ \sqrt{3} & 0 & -\sqrt{3}/2 & 2\cos(\Delta+\pi/6) & 4s & 2s \\ -2s & \sqrt{3}c-3s & \sqrt{3}c+3s & -\sin 2\Delta & 2x & 2y \\ \sqrt{3}d-3s & -2s & 0 & 2x & y & 2\sin 2\Delta \\ \sqrt{3}c+3s & 0 & -2s & 2y & 2\sin 2\Delta & -x \end{pmatrix}$$

$$M4^{(u)} = r_z\mathbf{I}+\mathcal{A}^2 \begin{pmatrix} -11 & -1 & 1 & -2c & 2w & 2z \\ -1 & -25/2 & -4 & 2w & -2c & -4c \\ 1 & -4 & -25/2 & 2z & -4c & -2c \\ -2c & 2w & 2z & -12+\cos 2\Delta & 2(u-1) & 2(1+v) \\ 2w & -2c & -4c & 2(u-1) & -12+\cos(2\Delta+10\pi/3) & -4c^2 \\ 2z & -4c & -2c & 2(v+1) & -4c^2 & -12+\cos(2\Delta+8\pi/3)\,, \end{pmatrix}$$

where $\mathbf{I}$ denotes the 6×6 identity matrix and $s=\sin\Delta, c=\cos\Delta, u=\sin(2\Delta+,\pi/6), v=\sin(2\Delta-\pi/6), x=\cos(2\Delta+\pi/6), y=\cos(2\Delta-\pi/6), w=\sin(\Delta-\pi/6), z=\sin(\Delta+\pi/6)$.

The coupling part in case of the low order product-type inter-map coupling energy is given by

$$M1^{(\alpha)} = \alpha \begin{pmatrix} -(6\mathcal{B}^2+\delta^2) & 2\mathcal{B}^2 & -2\mathcal{B}^2 & -\mathcal{B}^2 & 2\mathcal{B}(\mathcal{B}-\delta) & -2\mathcal{B}(\mathcal{B}-\delta) \\ 2\mathcal{B}^2 & -(6\mathcal{B}^2+\delta^2) & 2\mathcal{B}^2 & 2\mathcal{B}(\mathcal{B}-\delta) & -\mathcal{B}^2 & 2\mathcal{B}(\mathcal{B}-\delta) \\ -2\mathcal{B}^2 & 2\mathcal{B}^2 & -(6\mathcal{B}^2+\delta^2) & 2\mathcal{B}(-\mathcal{B}+\delta) & 2\mathcal{B}(\mathcal{B}-\delta) & -\mathcal{B}^2 \\ -\mathcal{B}^2 & 2\mathcal{B}(\mathcal{B}-\delta) & -2\mathcal{B}(\mathcal{B}-\delta) & -(6\mathcal{B}^2+\delta^2) & 2\mathcal{B}^2 & -2\mathcal{B}^2 \\ 2\mathcal{B}^2(\mathcal{B}-\delta) & -\mathcal{B}^2 & 2\mathcal{B}(\mathcal{B}-\delta) & 2\mathcal{B}^2 & -(6\mathcal{B}^2+\delta^2) & 2\mathcal{B}^2 \\ 2\mathcal{B}(-\mathcal{B}+\delta) & 2\mathcal{B}(\mathcal{B}-\delta) & -\mathcal{B}^2 & -2\mathcal{B}^2 & 2\mathcal{B}^2 & -(6\mathcal{B}^2+\delta^2) \end{pmatrix}$$

$M4^{(\alpha)} = M1^{(\alpha)}, M2^{(\alpha)} = M3^{(\alpha)} = 0.$

The coupling part in case of the low order gradient-type inter-map coupling energy is given by

$$M1^{(\beta)} = \beta\mathcal{B}^2 \begin{pmatrix} 3 & -5/4 & 5/4 & 1 & -5/4 & 5/4 \\ -5/4 & 3 & -5/4 & -5/4 & 1 & -5/4 \\ 5/4 & -5/4 & 3 & 5/4 & -5/4 & 1 \\ 1 & -5/4 & 5/4 & 3 & -5/4 & 5/4 \\ -5/4 & 1 & -5/4 & -5/4 & 3 & -5/4 \\ 5/4 & -5/4 & 1 & 5/4 & -5/4 & 3 \end{pmatrix}$$

$M4^{(\beta)} = M1^{(\beta)}, M2^{(\beta)} = M3^{(\beta)} = 0.$

The stationary amplitudes $\mathcal{A}$ are given in Eq. (63), Eq. (68), Eq. (81), and Eq. (83). The stationary amplitudes $\mathcal{B}$ and the constant δ are given by Eq. (94) and Eq. (116), respectively.
